# A network meta-analysis of the dose–response effects of lurasidone on acute schizophrenia

**DOI:** 10.1038/s41598-021-84836-z

**Published:** 2021-03-10

**Authors:** Manit Srisurapanont, Sirijit Suttajit, Surinporn Likhitsathian, Benchalak Maneeton, Narong Maneeton

**Affiliations:** Department of Psychiatry, Faculty of Medicine, Chiang Mai University, Si Phum, 110 Inthawarorot Road, Mueang, 50200 Chiang Mai Thailand

**Keywords:** Schizophrenia, Clinical pharmacology

## Abstract

We compared the efficacy, safety, and acceptability of lurasidone at different doses to establish the dose–response relationships of lurasidone therapeutic and adverse effects in acute schizophrenia. Included trials were 4- to 16-week, fixed-dose, randomized controlled trials of lurasidone in adults with acute schizophrenia. Different doses of lurasidone, other antipsychotics, and placebo were considered as independent treatments. Apart from all-cause dropout rates, four therapeutic and four adverse outcomes were included in the frequentist network meta-analysis (NMA). Lurasidone 160, 120, 80, 40, and 20 mg/day were studied in ten trials of 3,366 adults with schizophrenia exacerbation. Lurasidone 160 mg/day reduced Positive and Negative Syndrome Scale (PANSS) total scores significantly more than lurasidone 120, 80, 40, and 20 mg/day (mean differences = − 7.63, − 7.04, − 8.83, and − 12.25, respectively). All-cause dropout rates were significantly lower in participants receiving lurasidone 160 mg/day and 80 mg/day compared with those taking placebo. The half-maximal effective doses of lurasidone for PANSS total, PANSS positive, and MADRS score reductions were higher than 80 mg/day. The confidence of all NMA estimates was low or very low. Lurasidone 160 mg/day is currently the most efficacious and acceptable dose for acute schizophrenia. Its maximal effective doses may be higher than 160 mg/day.

## Introduction

Behavioral symptoms of schizophrenia are complex, and each syndrome may respond to different doses of an antipsychotic. Among the heterogeneous symptoms of schizophrenia, common syndromes include positive, negative, and depression syndromes^1^. The doses of quetiapine and aripiprazole effective for negative symptoms are more narrow than those effective for overall psychotic and positive symptoms^2,3^. These findings suggest that the dose–response relationships of antipsychotics may vary among schizophrenia syndromes. The findings of two pairwise meta-analyses (PMAs) suggested that the approved doses of 40–160 mg/day are effective for various syndromes of schizophrenia, as effective as most antipsychotics, well-tolerated, and less likely to cause weight gain^4,5^.

The evidence so far suggests that lurasidone doses and effects for acute schizophrenia may be inconsistent. Lurasidone 80 mg/day was not effective for acute schizophrenia in some randomized trials^6–8^ but outperformed both placebo and lurasidone 120 mg/day in another trial^9^. A recent network meta-analysis (NMA) found that lurasidone 40 mg/day increased body weight, but lurasidone 80 mg/day was associated with weight loss^10^. The product monograph of lurasidone informed that many adverse effects of lurasidone 120 mg/day are more common than those of lurasidone 160 mg/day, e.g., somnolence (26% vs. 8%), akathisia (22% vs. 7%)^11^. These findings suggest an inconsistent ordering of lurasidone doses and effects. The dose adjustment of lurasidone for treating acute schizophrenia is, therefore, challenging.

Little has been known about the dose–response relationships of lurasidone for schizophrenia syndromes and its common side effects. One NMA compared the clinical effects of only three treatments of lurasidone 80 mg/day, lurasidone 40 mg/day, and placebo. This NMA found that lurasidone 80 and 40 mg/day reduced most psychopathology to a similar extent^10^. A PMA compared the effects of the minimal effective dose (MED) of lurasidone with its 2- and threefold MEDs^12^. The 2- and threefold MEDs of lurasidone were superior to its MED in reducing overall and positive psychotic symptoms but not negative symptoms. Only one dose–response meta-analysis of lurasidone has been conducted. This meta-analysis used a multivariate statistical technique to synthesize the data, construct the dose–response curve, and estimate the effective dose of lurasidone for overall psychotic symptoms only^13^. It found that the maximal effective dose of lurasidone might be higher than 160 mg/day. The findings of these meta-analyses suggest that the dose–response relationships of lurasidone for many treatment effects remain unknown.

As an antipsychotic with variable treatment effects, the dose–response information of lurasidone treatment effects may guide its dose adjustment in managing schizophrenia syndromes and common adverse effects. This study aimed to compare the efficacy, safety, and acceptability of lurasidone at different doses to establish the dose–response relationships of lurasidone therapeutic and adverse effects in acute schizophrenia.

## Methods

### Protocol and registration

The protocol of this systematic review was prospectively registered at PROSPERO (CRD42020201337). The report of this NMA was based on the PRISMA 2015 Network Meta-Analysis Checklist (see STable 1)^14^. MS and SS independently screened the titles and abstracts, evaluated the full-text publications, selected the trials, extracted the data, and assessed the trial quality. If there was any discrepancy, these two reviewers resolved it using a consensus discussion.

### Eligible criteria

Included trials were 4- to 16-week, fixed-dose, randomized controlled trials (RCTs) of lurasidone in adults with acute schizophrenia that reported at least one outcome of interest. Trials or trial arms with a cross-over study design or examining the flexible doses of lurasidone were excluded. The data from trials or the trial arms that titrated the study medications in the first week could be included.

Age, sex, and severity of overall psychotic symptoms were considered as effect modifiers^15^. The medians and interquartile ranges of these modifiers were plotted to explore the transitivity among treatment groups.

### Information sources, searches, and study selection

We searched Pubmed, Scopus, Web of Science, Cochrane Library, and ClinicalTrials.gov from the inception. Key search terms included “lurasidone” AND “schizophrenia”. An additional limitation was applied by adding the term “random*” or limiting to “randomized-controlled trial”. The details of database searches can be found in STable 2. After the removal of duplicate records, we screened the titles/abstracts, evaluated the full-text publications, and selected the trials. No language restriction was applied for the study selection.

### Data collection process and data extraction

We extracted the trial data using a data record form. Trial characteristics of interest included: i) study ID (first author, year); ii) participant characteristics, including age, diagnosis, percentage of male participants, the severity of overall psychotic symptoms, and study duration; iii) each fixed dose of lurasidone, other antipsychotics, and placebo; iv) the measures of psychotic and depression syndromes; v) adverse outcomes; and vi) dropout rates.

### Data items

The primary outcomes of efficacy and acceptability were overall psychotic symptom reduction and all-cause dropout rates, respectively. Three secondary outcomes of efficacy included positive, negative, and depression symptom reduction. Four safety outcomes were weight gain, the incidence rates of somnolence, the incidence of extrapyramidal side effects (EPS), and dropout rates due to adverse events (or adverse dropout rates). The term “adverse dropout” used in this review referred to a participant who discontinued his/her assigned treatment, including placebo because he/she could not tolerate an adverse effect of that treatment. For each trial, we extracted only the last outcomes reported between 4 and 16 weeks.

### Geometry of the network

All fixed doses of lurasidone, other antipsychotics, and placebo were considered as independent treatments. For a network plot, a treatment was drawn by a node, and a comparison between the treatments was shown by an edge. The edge thickness indicated the number of comparisons.

### Risk of bias within individual trials

We assessed the trial quality using the revised tool to assess the risk of bias in randomized trials (RoB 2)^16^. This tool evaluated five domains of bias, including randomization processes, adherence to the assigned interventions, missing outcome data, the bias of measurement, and the bias of the reported results. Each domain was rated as low risk-of-bias, some concerns, or high risk-of-bias. The worst risk of bias in any of the domains was used for rating the overall risk of bias.

### Summary measures, data analysis, and assessment of inconsistency

We computed a mean difference (MD) if the continuous outcome was measured using the same scale. Otherwise, that outcome would be computed as a standardized mean difference (SMD). We aggregated the dichotomous outcomes using relative risks (RRs).

For each pairwise comparison, the negative MD (or SMD) indicated the superiority of a lurasidone dose against the other lurasidone dose, the other antipsychotic, or placebo. We compared the efficacy, safety, and acceptability between treatments using a pairwise meta-analysis and among treatments using a frequentist NMA. All analyses were performed on a random-effect model. For each comparison, we reported direct, indirect, and NMA estimates. We described the treatment ranks using league tables.

We assessed the global heterogeneity (incoherence) of a dataset using the Cochran chi-square (Cochran Q) statistic incoherence tests^17^. The analysis of Separate Indirect from Direct Evidence (SIDE) was performed using the back-calculation method to identify the local inconsistency between a pair of direct and indirect estimates^18^. The inconsistency with the p-value between 0.05 and 0.10 or less than 0.05 was considered as some concern or major concern, respectively.

The data were analyzed and visualized using the *netmeta* version 1.2–1 package under the *R Program* version 3.6 via the *Rstudio* software version 1.2.5^19–21^.

### Risks of bias across studies and additional analysis

We weighed the risks of bias using the sample sizes and plotted the risks of bias across trials. The publication bias was visualized using the funnel plots and quantified using the Egger’s test^22,23^.

### Confidence in NMA estimates

Although this NMA included the data of other antipsychotics, their treatment effects and ranks were disregarded. This decision was made because we did not comprehensively search and include all RCT data of other antipsychotics.

We used a semiautomated software that assessed the confidence of NMA estimates based on the Confidence in NMA approach (CINeMA)^24,25^. Four grades of confidence used for rating an NMA estimate included high, moderate, low, or very low levels. The confidence of NMA estimates derived from RCTs was rated as high and was downgraded based on the following concerns: (1) within-study bias, (2) reporting bias, (3) indirectness, (4) imprecision, (5) heterogeneity, and (6) incoherence. Suspected reporting bias or major concern on any dimension were rated down by two levels. Some concern on a dimension resulted in the downgrading of confidence by one level. We summarized the risk of bias and indirectness using the majority.

### Dose–response relationships

We performed graphic and quantitative exploration of dose–response relationships using the NMA estimates of lurasidone effects compared with those of placebo. Four therapeutic responses of interest included overall psychotic, positive, negative, and depression symptoms. Four adverse responses being examined were weight gain, somnolence, EPS, and adverse dropouts. Because all-cause dropout rates were the composite outcome of therapeutic and adverse effects, its dose–response relationship was not considered.

We plotted the doses and responses on the x- and y-axes, respectively. The best-fit curve was plotted using the Hill equation, and the half-maximal effective dose (ED50) was calculated. The dose–response relationships were analyzed and visualized using *Dr Fit* software version 1.042^26^.

## Results

### Study selection

**Figure 1:**
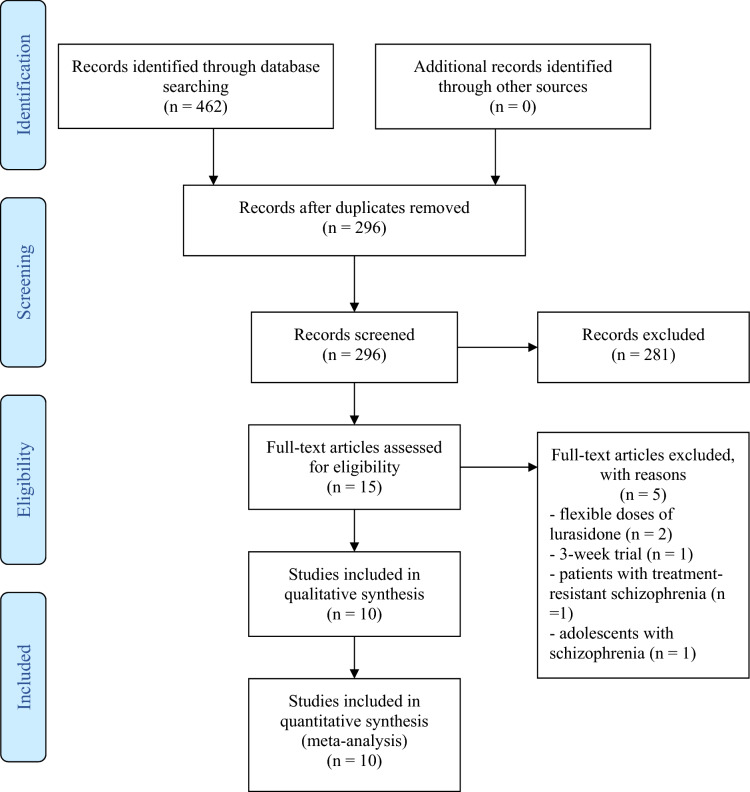
PRISMA flow diagram: records retrieved from database searches and trial inclusion in the systematic review and network meta-analysis of lurasidone doses for schizophrenia.

We searched the databases on July 29, 2020, and found 462 items (see Fig. 1). After the duplicate removal, 296 records remained for the title and abstract screening. We further assessed 15 full-text publications and excluded five trials, including two flexible-dose trials^27,28^, one 3-week trial^29^, one trial in adolescent patients^30^, and one trial in adults with treatment-resistant schizophrenia^31^. Finally, ten RCTs were included for network meta-analysis^6–9,32–37^.

### Network structure and geometry

**Figure 2:**
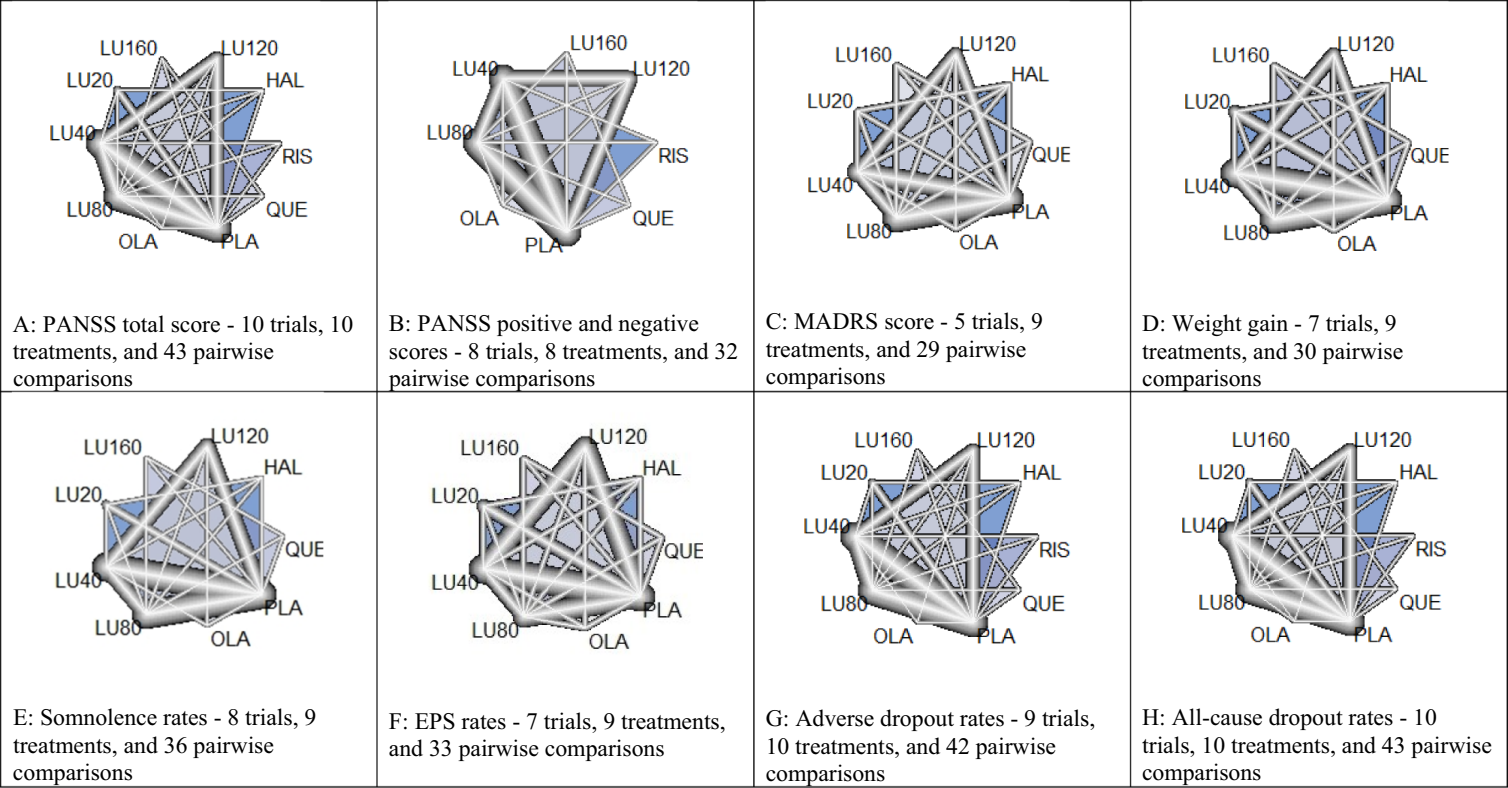
Network plots of five different doses of lurasidone, other antipsychotics, and placebo for acute schizophrenia. The nodes in the graph layout correspond to lurasidone at various doses, other antipsychotics, and placebo. Lines display the observed treatment comparisons. The thickness of edge indicates the number of comparisons. Gray/blue areas indicate the availability of trials with three doses or more. PANSS: Positive and Negative Syndrome Scale; MADRS: Montgomery-Asberg Depression Rating Scale; EPS: extrapyramidal symptoms. HAL: Haloperidol; LU20: Lurasidone 20 mg/day; LU40: Lurasidone 40 mg/day; LU80: Lurasidone 80 mg/day; Lurasidone 120 mg/day; Lurasidone 160 mg/day; OLA: Olanzapine; PLA: Placebo; QUE: Quetiapine; RIS: Risperidone.

The NMA compared the efficacy, acceptability, and safety among ten independent treatments, including five doses of lurasidone (160, 120, 80, 40, and 20 mg/day), four antipsychotics (i.e., haloperidol, olanzapine, quetiapine, and risperidone), and placebo. Figure 2A–H show the network graphs of nine outcomes. The largest network was the dataset of Positive and Negative Syndrome Scale (PANSS) total score used for assessing the severity of overall psychotic symptoms (10 trials, 10 treatments, and 43 pairwise comparisons). The smallest network was the dataset of Montgomery-Asberg Depression Rating Scale (MADRS) scores used for assessing the severity of depression symptoms (5 trials, 9 treatments, and 29 pairwise comparisons). The networks of PANSS positive and negative scores were the same and included only four doses of lurasidone (160, 120, 80, and 40 mg/day). All five doses of lurasidone (160, 120, 80, 40, and 20 mg/day) were involved in the rest networks.

### Study characteristics

After excluding two arms of lurasidone flexible doses^33^, 3,366 adults with acute schizophrenia of ten trials were included in this NMA (see Table 1). All were six-week trials. Nine trials had a placebo-controlled arm. Other antipsychotics and their doses (number of participants) were as follows: haloperidol 10 mg/day (n = 72), olanzapine 10 (n = 51) and 15 mg/day (n = 123), quetiapine 600 mg/day (n = 120), and risperidone 4 mg/day (n = 64). Three age ranges of the participants included 18–75 years in six trials, 18–65 years in three trials, and 18–45 years in one trial^32^.

**Table 1:** Characteristics and key results of the included trials^a^.

Trial (year)	Duration (weeks)	Lurasidone dose (mg/day)	Control/comparator (mg/day)	Patient population	Available outcomes of interest (measures)—results^b,c^
Nakamura 2009	6	80 mg/day (*n* = 90)	Placebo (*n* = 90)	Schizophrenia, acute exacerbation of symptoms; age 18–64 years	Overall psychotic symptoms (PANSS total score): LU80 > PLA Positive symptoms (PANSS positive score): LU80 > PLA Negative symptoms (PANSS negative score): LU80 > PLA Depression (MADRS score): LU80 > PLA Weight gain: N/A Somnolence rates: N/A Adverse dropout rates: LU80 ≈ PLA All-cause dropout rates: LU80 ≈ PLA
Meltzer 2011	6	40 mg/day (*n* = 120) 120 mg/day (*n* = 119)	Placebo (*n* = 116) Olanzapine 15 mg/day (*n* = 123)	Schizophrenia, acute exacerbation of symptoms; age 18–75 years	Overall psychotic symptoms (PANSS total score): LU40, LU120, OLA > PLA Positive symptoms (PANSS positive score): LU40, LU120, OLA > PLA Negative symptoms (PANSS negative score): LU40, LU120, OLA > PLA Depression (MADRS score): OLA > PLA Weight gain: LU40, LU120 ≈ PLA; OLA > PLA Somnolence rates: N/A EPS rates: N/A Adverse dropout rates: N/A All-cause dropout rates: N/A
Loebel 2013	6	80 mg/day (*n* = 125) 160 mg/day (*n* = 121)	Placebo (*n* = 122) Quetiapine 600 mg/day (*n* = 120)	Schizophrenia, acute exacerbation of symptoms; age 18–75 years;	Overall psychotic symptoms (PANSS total score): LU80, LU160, QUE > PLA Positive symptoms (PANSS positive score): LU80, LU160, QUE > PLA Negative symptoms (PANSS negative score): LU80, LU160, QUE > PLA Depression (MADRS score): LU80, LU160, QUE > PLA Weight gain: LU80, QUE > PLA Somnolence rates: N/A EPS rates: N/A Adverse dropout rates: N/A All-cause dropout rates: N/A
Nasrallah 2013	6	40 mg/day (*n* = 125) 80 mg/day (*n* = 123) 120 mg/day (*n* = 124)	Placebo (*n* = 128)	Schizophrenia, acute exacerbation of symptoms; age 18–75 years	Overall psychotic symptoms (PANSS total score): LU80 > PLA; LU40, LU120 ≈ PLA Positive symptoms (PANSS positive score): LU80, LU120 > PLA; LU40 ≈ PLA Negative symptoms (PANSS negative score): LU40, LU80, LU120 ≈ PLA Depression (MADRS score): LU40, LU80, LU120 ≈ PLA Somnolence rates: N/A EPS rates: N/A Adverse dropout rates: N/A All-cause dropout rates: N/A
Ogasa 2012	6	40 mg/day (*n* = 50 120 mg/day (*n* = 49)	Placebo (*n* = 50)	Schizophrenia, acute exacerbation of symptoms; age 18–64 years	Overall psychotic symptoms (PANSS total score): LU120 > PLA; LU40 ≈ PLA Positive symptoms (PANSS positive score): LU40, LU 120 > PLA Negative symptoms (PANSS negative score): LU120 > PLA; LU40 ≈ PLA Weight change: LU40, LU 120 ≈ PLA Somnolence rates: N/A EPS rates: N/A Adverse dropout rates: N/A All-cause dropout rates: N/A
Potkin 2015	6	20 mg/day (n = 71) 40 mg/day (n = 67) 80 mg/day (n = 71)	Placebo (*n* = 72) Haloperidol 10 md/day (*n* = 72)	Schizophrenia, acute exacerbation of symptoms; age 18–64 years	Overall psychotic symptoms (PANSS total score): LU20, LU40, LU80, HAL ≈ PLA Depression (MADRS score): LU20, LU40, LU80, HAL ≈ PLA Weight change: N/A Somnolence rates: N/A EPS rates: N/A Adverse dropout rates: N/A All-cause dropout rates: N/A
Loebel 2016^d^	6	20 mg/day (n = 112	Placebo (*n* = 101)	Schizophrenia, acute exacerbation of symptoms; age 18–75 years	Overall psychotic symptoms (PANSS total score): LU20 ≈ PLA Weight change: LU20 ≈ PLA Somnolence rates: N/A EPS rates: N/A Adverse dropout rates: N/A All-cause dropout rates: N/A
Higuchi 2019a	6	40 mg/day (n = 150) 80 mg/day (n = 155)	Placebo (*n* = 152)	Schizophrenia, acute exacerbation of symptoms; age 18–74 years	Overall psychotic symptoms (PANSS total score): LU40, LU80 ≈ PLA Positive symptoms (PANSS positive score): LU40 PLA; LU80 > PLA Negative symptoms (PANSS negative score): LU40, LU80 ≈ PLA Weight change: LU40, LU80 ≈ PLA Somnolence rates: N/A Adverse dropout rates: N/A All-cause dropout rates: N/A
Higuchi 2019b	6	40 mg/day (n = 125) 80 mg/day (n = 129)	Placebo (*n* = 152) Risperidone 4 mg/day (*n* = 64)	Schizophrenia; age 18–75 years	Overall psychotic symptoms (PANSS total score): LU40, LU80, RIS ≈ PLA Positive symptoms (PANSS positive score): RIS > PLA; LU40, LU80 ≈ PLA Negative symptoms (PANSS negative score): LU40, LU80, RIS ≈ PLA Adverse dropout rates: N/A All-cause dropout rates: N/A
Jena 2019	6	80 mg/day (n = 51)	Olanzapine 10 mg/day (*n* = 50)	Schizophrenia; age 18–45 years	Overall psychotic symptoms (PANSS total score): OLA > LU80 Positive symptoms (PANSS positive score): OLA > LU80 Negative symptoms (PANSS negative score): OLA LU80 All-cause dropout rates: N/A

^a^Only the last results of the study.

^b^The symbols of > and  ≈ indicate significant superiority (*p* < 0.05) and not significant difference (p ≥ 0.05) as being reported by the authors, respectively.

^c^N/A indicates the availability of data but no statistical test being applied.

^d^Participants initiating with lurasidone 80 mg/day were excluded. Those nonresponding to 2-week treatment of lurasidone 80 mg/day were randomized to further received lurasidone 80 mg/day and lurasidone 160 mg/day.

PANSS: Positive and Negative Syndrome Scale; MADRS: Montgomery-Asberg Depression Rating Scale; EPS: extrapyramidal symptoms.

LU20, LU40, LU80, LU120, LU160: Lurasidone 20, 40, 80, 120, 160 mg/day, respectively.

HAL = haloperidol; OLA = olanzapine; PLA = placebo; QUE = quetiapine; and RIS = risperidone.

Mean age, sex, and severity of overall psychotic symptoms (based on PANSS total score) of each treatment were summarized and visualized as median and interquartile range (see SFig. 1A–C). These effect modifiers showed no considerable difference among the treatments. We, therefore, assumed that there was no intransitivity of the data.

### Risk of bias in individual trials

All included trials applied the randomization study design (see SFig. 2A). Except for one trial^32^, nine ones implemented the double-blindness method. Of these, one trial did not describe the randomization process and the concealment of allocation^8^. These two trials were, therefore, rated as trials with a high risk of bias. Two trials had some concerns on the methods used to ensure the allocation concealment, which resulted in the trial rating of a moderate risk of bias^6,7^.

### Results of individual trials

Compared to placebo, the efficacy of lurasidone in reducing overall psychotic symptoms was as follows: (1) lurasidone 20 mg/day was not significantly effective in two trial^8,33^, (2) lurasidone 40 mg/day was significantly effective in one^35^ but not in five trials^6–9,37^, (3) lurasidone 80 mg/day was significantly effective in three^9,34,36^ but not in the other three trials ^6–8^, (4) lurasidone 120 mg/day was significantly effective in two^35,37^ but not in one trial^9^, and (5) lurasidone 160 mg/day was significantly effective in one trial^34^ (see Table 1).

Most trials reported the dichotomous data of somnolence, EPS, adverse dropout, and all-cause dropout rates, but only one trial reported that all-cause dropout rates between lurasidone 80 mg/day and placebo were not significantly different^36^. Other treatment outcomes reported in individual trials can be found in Table 1.

### Synthesis of results

All continuous outcomes were assessed using the same scales and, therefore, aggregated using the (weighted) mean differences (MDs) of scale scores. The scales used were as follows: PANSS total scores for the severity of overall psychotic symptoms, PANSS positive scores for the severity of positive symptoms, PANSS negative scores for the severity of negative symptoms, MADRS scores for the severity of depression symptoms, and kilograms for weight gain.

**Figure 3:**
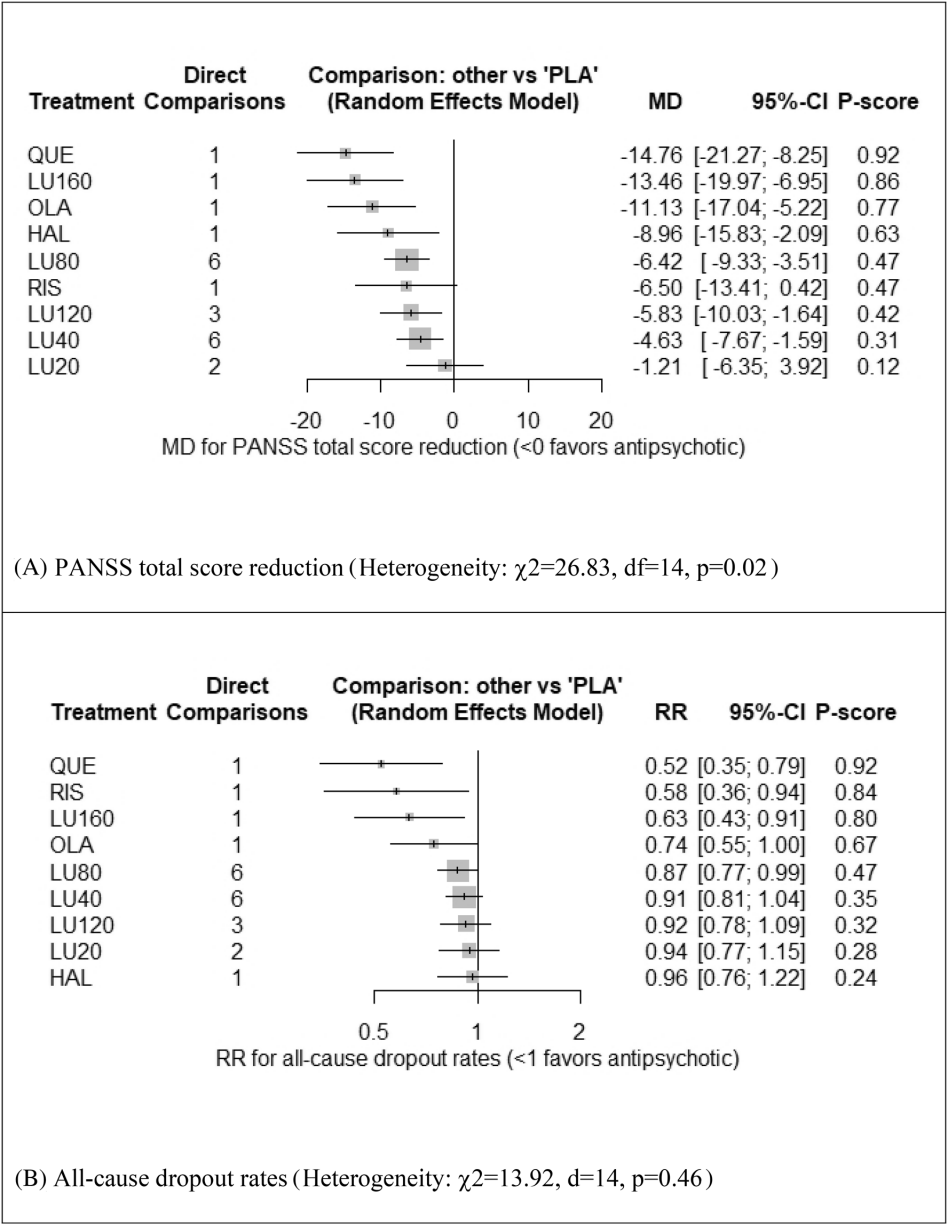
Forest plots: the efficacy and acceptability of lurasidone at different doses and other antipsychotics compared to placebo and the global heterogeneity (inconsistency) of data. MD: mean difference; RR: relative risk; P-score: p-value for treatment ranking. PANSS: Positive and Negative Syndrome Scale; LU20, LU40, LU80, LU120, LU160: Lurasidone 20, 40, 80, 120, 160 mg/day, respectively. HAL = haloperidol; OLA = olanzapine; PLA = placebo; QUE = quetiapine; and RIS = risperidone.

Figure 3A shows the forest plot of pooled MDs comparing PANSS total score reduction between five lurasidone doses and placebo. Except for lurasidone 20 mg/day, lurasidone 160, 120, 80, and 40 mg/day was significantly superior to placebo. Among these four doses, lurasidone 160 mg/day was the most effective (MD = − 13.46, 95%CI: − 19.97 to − 6.95). Table 2A shows that lurasidone 160 mg/day significantly outperformed lurasidone 120, 80, 40, and 20 mg/day. Lurasidone 80 mg/day ranked higher than but was not significantly superior to lurasidone 120 mg/day (MD = − 0.59, 95%CI: − 5.10 to 3.93).

**Table 2:** League tables presenting network meta-analysis estimates: (lower triangle) and direct estimates (upper triangle): primary efficacy and acceptability outcomes of lurasidone at different doses and placebo for schizophrenia.

(A) SMDs for the reduction of PANSS-total score (95% CIs)^a^									
***Quetiapine 600 mg/day***	− 1.30 (− 8.63 to 6.03)	–	–	− 5.60 (− 12.93 to 1.73)	–	–	–	–	− **17.50 (**− **24.83 to **− **10.17)**
− 1.30 (− 8.63 to 6.03)	***Lurasidone 160 mg/day***	–	–	− 4.30 (− 11.63 to 3.03)	–	–	–	–	− **16.20 (**− **23.53 to **− **8.87)**
− 3.63 (− 12.23 to 4.97)	− 2.33 (− 10.93 to 6.27)	***Olanzapine 10–15 mg/day***	–	− 8.00 (− 20.94 to 4.94)	–	− 5.10 (− 12.82 to 2.62)	− 3.00 (− 10.62 to 4.62)	–	− **12.70 (**− **20.42 to **− **4.98)**
− 5.80 (− 15.08 to 3.48)	− 4.50 (− 13.78 to 4.78)	− 2.17 (− 10.98 to 6.64)	***Haloperidol 10 mg/day***	− 2.40 (− 10.73 to 5.93)	–	–	− **8.80 (**− **17.24 to **− **0.36)**	− **8.90 (**− **17.23 to **− **0.57)**	− 3.70 (− 12.03 to 4.63)
− **8.34 (**− **14.85 to **− **1.83)**	− **7.04 (**− **13.55 to **− **0.53)**	− 4.71 (− 10.78 to 1.36)	− 2.54 (− 9.52 to 4.44)	***Lurasidone 80 mg/day***	2.80 (− 5.07 to 10.67)	− 2.90 (− 10.23 to 4.43)	− 1.78 (− 5.51 to 1.95)	− 6.50 (− 14.83 to 1.83)	− **5.74 (**− **8.80 to **− **2.68)**
− 8.26 (− 17.55 to 1.02)	− 6.96 (− 16.25 to 2.32)	− 4.63 (− 13.44 to 4.18)	− 2.46 (− 11.97 to 7.04)	0.08 (− 6.88 to 7.03)	***Risperidone 4 mg/day***	–	− 1.00 (− 8.96 to 6.96)	–	− 4.60 (− 12.47 to 3.27)
− **8.93 (**− **16.48 to **− **1.37)**	− **7.63 (**− **15.18 to **− **0.07)**	− 5.30 (− 11.58 to 0.99)	− 3.13 (− 10.91 to 4.65)	− 0.59 (− 5.10 to 3.93)	− 0.67 (− 8.45 to 7.12)	***Lurasidone 120 mg/day***	− 0.55 (− 5.15 to 4.05)	–	− **6.77 (**− **11.41 to **− **2.12)**
− **10.13 (**− **17.07 to **− **3.18)**	− **8.83 (**− **15.77 to **− **1.88)**	− **6.50 (**− **12.44 to **− **0.56)**	− 4.33 (− 11.34 to 2.68)	− 1.79 (− 5.11 to 1.53)	− 1.87 (− 8.85 to 5.12)	− 1.20 (− 5.42 to 3.02)	***Lurasidone 40 mg/day***	− 0.10 (− 8.54 to 8.34)	− **3.80 (**− **6.96 to **− **0.64)**
− **13.55 (**− **21.70 to **− **5.39)**	− **12.25 (**− **20.40 to **− **4.09)**	− **9.92 (**− **17.56 to **− **2.27)**	− **7.75 (**− **15.37 to **− **0.13)**	− 5.21 (− 10.73 to 0.31)	− 5.28 (− 13.73 to 3.16)	− 4.62 (− 11.05 to 1.81)	− 3.42 (− 8.99 to 2.16)	***Lurasidone 20 mg/day***	0.64 (− 4.95 to 6.22)
− **14.76 (**− **21.27 to **− **8.25)**	− **13.46 (**− **19.97 to **− **6.95)**	− **11.13 (**− **17.04 to **− **5.22)**	− **8.96 (**− **15.83 to **− **2.09)**	− **6.42 (**− **9.33 to **− **3.51)**	− 6.50 (− 13.41 to 0.42)	− **5.83 (**− **10.03 to **− **1.64)**	− **4.63 (**− **7.67 to **− **1.59)**	− 1.21 (− 6.35 to 3.92)	***Placebo***

(B) RR for the All-Cause Dropout Rates (95% CIs)^b^									
***Quetiapine 600 mg/day***	–	0.83 (0.51–1.35)	–	0.67 (0.42–1.05)	–	–	–	–	0.49 (0.32–0.75)
0.90 (0.48–1.70)	***Risperidone 4 mg/day***	–	–	0.60 (0.36–1.01)	0.70 (0.41–1.19)	–	–	–	0.59 (0.35–0.98)
0.83 (0.51–1.35)	0.92 (0.50–1.68)	***Lurasidone 160 mg/day***	–	0.80 (0.52–1.23)	–	–	–	–	0.59 (0.40–0.87)
0.70 (0.43–1.16)	0.78 (0.44–1.37)	0.85 (0.53–1.36)	***Olanzapine 10–15 mg/day***	0.78 (0.22–2.75)	0.88 (0.62–1.26)	0.71 (0.51–0.99)	–	–	0.82 (0.58–1.15)
**0.60 (0.40–0.90)**	0.66 (0.41–1.08)	0.72 (0.50–1.05)	0.85 (0.62–1.16)	***Lurasidone 80 mg/day***	0.98 (0.82–1.15)	0.96 (0.66–1.39)	0.91 (0.69–1.20)	0.94 (0.71–1.25)	0.88 (0.77–1.01)
**0.57 (0.37–0.87)**	0.63 (0.39–1.03)	0.69 (0.47–1.01)	0.81 (0.60–1.09)	0.96 (0.82–1.11)	***Lurasidone 40 mg/day***	0.99 (0.82–1.19)	0.94 (0.72–1.23)	0.97 (0.74–1.29)	0.94 (0.83–1.07)
**0.56 (0.36–0.88)**	0.63 (0.38–1.04)	0.68 (0.46–1.02)	0.80 (0.60–1.08)	0.95 (0.78–1.15)	0.99 (0.83–1.17)	***Lurasidone 120 mg/day***	–	–	0.90 (0.75–1.07)
**0.55 (0.35–0.87)**	0.61 (0.36–1.03)	0.67 (0.44–1.01)	0.79 (0.56–1.11)	0.93 (0.75–1.14)	0.97 (0.78–1.19)	0.98 (0.76–1.26)	***Lurasidone 20 mg/day***	1.04 (0.80–1.35)	0.99 (0.79–1.25)
**0.54 (0.34–0.86)**	0.60 (0.35**–**1.02)	0.65 (0.42**–**1.01)	0.77 (0.54**–**1.11)	0.91 (0.71**–**1.15)	0.95 (0.75**–**1.20)	0.96 (0.73**–**1.26)	0.98 (0.76**–**1.26)	***Haloperidol 10 mg/day***	1.13 (0.85**–**1.51)
**0.52 (0.35–0.79)**	**0.58 (0.36–0.94)**	**0.63 (0.43–0.91)**	0.74 (0.55**–**1.00)	**0.87 (0.77–0.99)**	0.91 (0.81**–**1.04)	0.92 (0.78**–**1.09)	0.94 (0.77**–**1.15)	0.96 (0.76**–**1.22)	***Placebo***

^a^A mean difference (MD) less than 0 indicates the superiority of treatment defined in the column over the other treatment defined in the row.

^b^An odd ratio (OR) less than 1 indicates the fewer events of treatment defined in the column over the other treatment defined in the row.

− indicates the nonavailability of direct estimate.

Treatments are reported in order of ranking of efficacy. Comparison treatments should be read from left to right, and the MD or RR is in the cell in common between the column-defining treatment and the row-defining treatment. A bold estimate indicates the significant difference between the treatment pair.

Figure 3B shows the forest plot of pooled RRs comparing all-cause dropout rates associated with five lurasidone doses and placebo. Lurasidone 160 mg/day and 80 mg/day were associated with significantly fewer rates of all-cause dropouts compared to placebo (RR = 0.63, 95%CI = 0.43 to 0.91 and RR = 0.87, 95% CI 0.77 to 0.99, respectively). All-cause dropout rates among five lurasidone doses were not significantly different (see Table 2B).

The forest plots and league tables of other outcomes can be found in SFig. 3 and STable 3. The treatment effects on PANSS positive scores was similar to that of PANSS total scores (see SFig. 3A and STable 3A). For PANSS negative score reduction, lurasidone 160, 120, 80, and 40 mg/day were significantly superior to placebo, but these four doses were not significantly different among each other (see SFig. 3B and STable 3B). Together with lurasidone 160 mg/day, lurasidone 80 mg/day also significantly outperformed lurasidone 120 mg/day (MD = − 1.86, 95%CI: − 2.99 to − 0.73) and placebo in reducing MADRS score (see SFig. 3^3^ and STable 3C). Weight gain associated with lurasidone 80 and 40 mg/day was significantly higher than that with placebo (see SFig. 3D and STable 3D). Somnolence related to lurasidone 160, 120, 80, and 40 mg/day was significantly more common than that to placebo (see SFig. 3E and STable 3E). The EPS rates associated with lurasidone 120 and 40 mg/day were significantly higher than that related to placebo (see SFig. 3F and STable 3F). The adverse dropout rates associated with lurasidone 160, 120, 80, and 40 mg/day were not significantly higher than that related to placebo (see SFig. 3G and STable 3G).

### Heterogeneity and inconsistency

Among the nine outcomes, global heterogeneity (incoherence) was a major concern only for the dataset of PANSS total score reduction (χ^2^ = 26.83, df = 14, p = 0.02) (Fig. 3A). There was no concern about the global heterogeneity of data related to all-cause dropout rates (χ2 = 13.92, d = 14, *p* = 0.46) (Fig. 3B) and the data of the other seven outcomes (*p* > 0.10) (see SFig. 3A–G). Major concerns and some concerns for the local inconsistency can be found in STable 4A–I.

### Risks of bias across studies and additional analysis

SFigure 2B shows the risks of bias across trials weighted by the sample sizes. The high risks of bias in two domains and the overall risk of bias were less than 25%. SFig. 4A–I show the funnel plots for exploring the publication bias of nine outcomes. Of these, significant publication biases were found in the datasets of weight gain and adverse dropout rates (Egger’s test *p* < 0.01 and p = 0.01, respectively).

### Confidence of NMA estimates

Although the literature search retrieved published and unpublished information (e.g., clinictrials.gov), reporting bias of all NMA estimates was suspected because nine of ten trials were industry-funded. This domain, therefore, needed no further suspect related to the publication bias. Indirectness was not an issue of concern because all participants were adults with acute schizophrenia. Moreover, we found no obvious intransitivity of the effect modifiers. For the imprecision, we set the meaningful sizes of effect for the continuous outcomes as follows: (1) a 15-point reduction of PANSS total scale^38^, (2) a 3.5-point reduction of 7-item PANSS positive or negative subscale, (3) 2-point reduction of MADRS^39^ and (4) 0.9 kg of weight gain. The 15-point reduction of 30-item PANSS total scale was used to proportionally set the 3.5-point reduction of 7-item PANSS positive or negative subscale. In a large RCT, olanzapine increased the body weight of patients with schizophrenia by an average of 0.9 kg/month^40^. These cut-offs were used to set the imprecision of NMA estimates. STable 5A–I shows the risk-of-bias charts of all outcomes.

After implementing the judgments and calculations on the issues mentioned above, all NMA estimates were rated as low and very low confident (see STable 5A–I). The low and very low confidence levels were mainly caused by the suspect of reporting bias, which resulted in rating down the confidence by two levels from high to low levels. Table 3A,B and STable 6A–G show the confidence rating and effect estimates of each comparison among lurasidone doses and placebo.

**Table 3:** Confidence rating and effect estimates of each comparison: lurasidone at different doses vs. placebo for acute schizophrenia.

Treatment comparisons	Direct estimate: RR (95% CI)	Indirect estimate: RR (95% CI)	NMA estimate: RR (95% CI)	Confidence rating
**(A) PANSS-total score reduction**				
LU160 vs. Placebo	− 16.20 [− 23.53; − 8.87]	− 3.19 [− 17.37; 11.00]	− **13.46 [**− **19.97; **− **6.95]**	Low
LU120 vs. Placebo	− 6.77 [− 11.41; − 2.12]	− 1.72 [− 11.47; 8.03]	− **5.83 [**− **10.03; **− **1.64]**	Low
LU80 vs. Placebo	− 5.74 [− 8.80; − 2.68]	− 12.73 [− 22.06; − 3.40]	− **6.42 **[− **9.33; **− **3.51]**	Low
LU40 vs. Placebo	− 3.80 [− 6.96; − 0.64]	− 14.66 [− 25.63; − 3.68]	− **4.63 **[− **7.67; **− **1.59]**	Very low
LU20 vs. Placebo	0.64 [− 4.95; 6.22]	− 11.22 [− 24.22; 1.78]	− 1.21 [− 6.35; 3.92]	Low
LU160 vs. LU80	− 4.30 [− 11.63; 3.03]	− 17.31 [− 31.50; − 3.13]	− **7.04 [**− **13.55; **− **0.53]**	Very low
LU160 vs. LU40	−	− 8.83 [− 15.77; − 1.88]	− **8.83 [**− **15.77; **− **1.88]**	Very low
LU160 vs. LU20	−	− 12.25 [− 20.40; − 4.09]	− **12.25 [**− **20.40; **− **4.09]**	Very low
LU120 vs. LU160	−	7.63 [0.07; 15.18]	**7.63 **[**0.07; 15.18]**	Very low
LU120 vs. LU80	2.90 [− 4.43; 10.23]	− 0.83 [− 6.57; 4.91]	0.59 [− 3.93; 5.10]	Low
LU120 vs. LU40	− 0.55 [− 5.15; 4.05]	− 4.73 [− 15.42; 5.95]	− 1.20 [− 5.42; 3.02]	Low
LU120 vs. LU20	−	− 4.62 [− 11.05; 1.81]	− 4.62 [− 11.05; 1.81]	Very low
LU40 vs. LU80	1.78 [− 1.95; 5.51]	1.83 [− 5.45; 9.11]	1.79 [− 1.53; 5.11]	Very low
LU20 vs. LU80	6.50 [− 1.83; 14.83]	4.19 [− 3.18; 11.57]	5.21 [− 0.31; 10.73]	Very low
LU20 vs. LU40	0.10 [− 8.34; 8.54]	5.99 [− 1.44; 13.42]	3.42 [− 2.16; 8.99]	Very low

Treatment comparisons	Direct estimate: RR (95% CI)	Indirect estimate: RR (95% CI)	NMA estimate: RR (95% CI)	Confidence rating
**(B) For all-cause dropout rates**				
LU160 vs. Placebo	0.59 [0.40; 0.87]	1.13 [0.36; 3.51]	**0.63 [0.43; 0.91]**	Low
LU120 vs. Placebo	0.90 [0.75; 1.07]	1.13 [0.70; 1.82]	0.92 [0.78; 1.09]	Very low
LU80 vs. Placebo	0.88 [0.77; 1.01]	0.82 [0.53; 1.26]	**0.87 [0.77; 0.99]**	Very low
LU40 vs. Placebo	0.94 [0.83; 1.07]	0.60 [0.37; 0.98]	0.91 [0.81; 1.04]	Very low
LU20 vs. Placebo	0.99 [0.79; 1.25]	0.82 [0.55; 1.21]	0.94 [0.77; 1.15]	Very low
LU160 vs. LU80	0.80 [0.52; 1.23]	0.49 [0.22; 1.10]	0.72 [0.50; 1.05]	Very low
LU160 vs. LU40	−	0.69 [0.47; 1.01]	0.69 [0.47; 1.01]	Very low
LU160 vs. LU20	−	0.67 [0.44; 1.01]	0.67 [0.44; 1.01]	Very low
LU120 vs. LU160	−	1.47 [0.98; 2.20]	1.47 [0.98; 2.20]	Very low
LU120 vs. LU80	1.05 [0.72; 1.52]	1.06 [0.85; 1.33]	1.06 [0.87; 1.28]	Very low
LU120 vs. LU40	1.01 [0.84; 1.22]	1.00 [0.64; 1.57]	1.01 [0.85; 1.20]	Very low
LU120 vs. LU20	−	0.98 [0.76; 1.26]	0.98 [0.76; 1.26]	Very low
LU40 vs. LU80	1.03 [0.87; 1.21]	1.12 [0.83; 1.51]	1.05 [0.90; 1.21]	Very low
LU20 vs. LU80	1.10 [0.84; 1.45]	1.05 [0.75; 1.47]	1.08 [0.87; 1.34]	Very low
LU20 vs. LU40	1.06 [0.81; 1.40]	0.99 [0.71; 1.37]	1.03 [0.84; 1.27]	Very low

PLA: Placebo; LU20, LU40, LU80, LU120, LU160: Lurasidone 20, 40, 80, 120, 160 mg/day, respectively.

### Dose–response relationships

Four doses of 160, 120, 80, and 40 mg/day were included in the dose–response analyses of their interactions to positive and negative symptoms. For the other six outcomes, five doses of 160, 120, 80, 40, and 20 mg/day were involved in the analyses.

**Figure 4:**
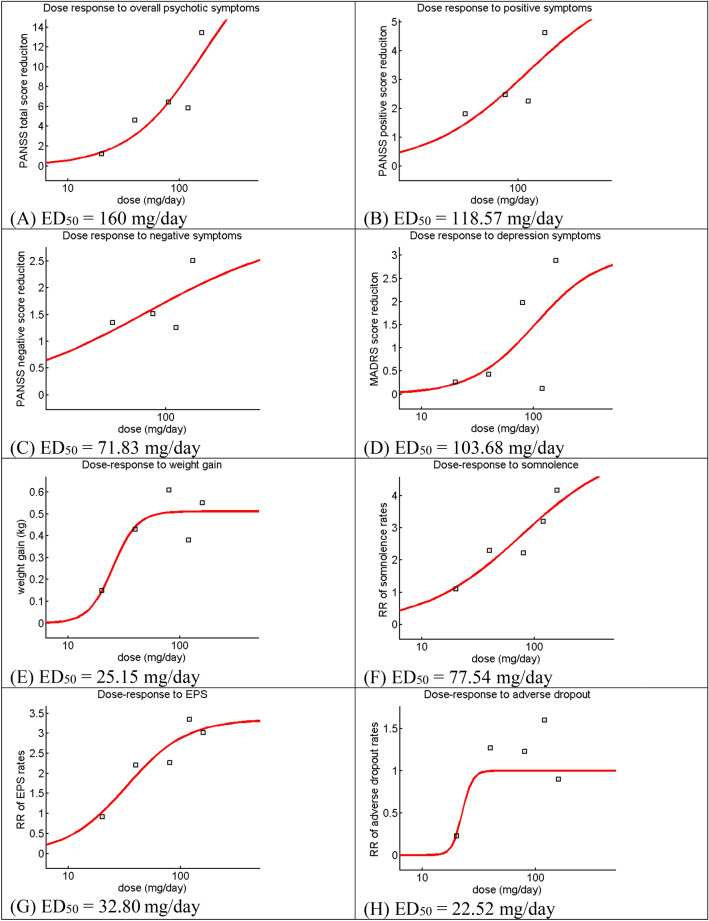
Dose–response curves: therapeutic and adverse effects of lurasidone at different doses for acute schizophrenia. Each curve was plotted with the lurasidone doses on the x-axis and the point NMA estimates of lurasidone against placebo on the y-axis. Figure 4A and 4D–4H include five doses of 160, 120, 80, 40, and 20 mg/day. Figure 4B and 4C include four doses of 160, 120, 80, and 40 mg/day. ED_50_ = half-maximal effective dose, EPS: Extrapyramidal side effects, PANSS: Positive and Negative Syndrome Scale, MADRS: Montgomery-Asberg Depression Rating Scale.

Figure 4 shows the dose–response curves and ED50 of eight outcomes derived from the NMA estimates comparing lurasidone at different doses and placebo. Together with the nonexistence of plateau, the ED50′s of lurasidone for PANSS total, PANSS positive, and MADRS score reductions were higher than 80 mg/day. For the rest outcomes, the plateaus could be observed, and the ED50′s were lower than 80 mg/day.

## Discussion

Lurasidone 160, 120, 80, and 40 mg/day are effective for treating acute schizophrenia, and the dose of 160 mg/day outperforms the other three doses. Only two doses of 160 and 80 mg/day are associated with fewer dropouts and can reduce depression symptoms. The findings of ED50’s larger than 80 mg/day suggest that the maximal effective doses for PANSS total score, PANSS positive score, and MADRS score reductions might be higher than 160 mg/day. However, lurasidone might produce the maximum effects for negative symptoms and the other four adverse outcomes at the doses between 40 and 160 mg/day.

The present findings are in line with those of a PMA^5^. Our NMA estimates confirm the efficacy of lurasidone 40–160 mg/day for mitigating overall psychotic and positive symptoms of schizophrenia. The similar adverse-dropout rates of lurasidone 40–160 mg/day and placebo suggest the mild adverse effects of lurasidone. During the 6-week treatment of lurasidone, the patients may gain weight for 0.61 kg or less. Lurasidone 40–160 mg/day may increase a two- to four-fold risk of somnolence and EPS for two to four fold.

The findings of this study are similar to those of a PMA but not a NMA comparing the different doses of lurasidone. This study confirms previous PMA findings that the higher doses of lurasidone reduce psychotic symptoms to a greater extent and increase the risks of side effects^12^. However, this study also found the plateau of weight gain and adverse dropouts within the dose range of 40–80 mg/day. The present findings did not support the similar efficacy of lurasidone 80 and 40 mg/day reported in a previous NMA^10^.

Despite the uses of different statistical techniques and datasets, the dose–response relationships of lurasidone for overall psychotic symptoms found in a previous meta-analysis and this study were relatively similar^13^. Leucht and colleagues (2020) conducted their meta-analysis using a multivariate statistical technique to plot both meta-analysis estimates and their 95% CIs^41^. However, the present study applied the widely-used NMA techniques to compare the treatment effects of the different doses of lurasidone and fitted the dose–response curves using the NMA estimates of the different doses. Although this study could compute both point estimates and their 95%CIs, only the point estimates from NMA were used for curve fitting. The 95%CI values were disregarded because the superimposing of three dose–response curves of point estimates and their upper and lower bounds may result in a crossing of these curves, which is difficult for interpretation. Despite of the above-mentioned differences, the ED50 of lurasidone for overall psychotic symptoms (160 mg/day) found in this study still confirms the previous finding that the maximal effective dose of lurasidone for overall psychotic symptoms may be higher than 160 mg/day.

Each schizophrenia syndrome and lurasidone adverse effect has its pattern of doses and responses interaction. The increase of lurasidone doses from 40 mg/day to somewhere higher than 160 mg/day may increase lurasidone therapeutic effects for overall psychotic, positive, and depression symptoms, but not negative symptoms. The increase of lurasidone doses between 40 and 160 mg/day may increase somnolence and ESP severity. Comparison to its product monograph^11^, we did find the plateau of weight gain within the dose range of 40–80 mg/day but did not find the peak of EPS rates at the dose of 120 mg/day. The current findings would be helpful to guide the dose adjustment of lurasidone for acute schizophrenia.

Our findings support the use of high-dose lurasidone for acute schizophrenia. Lurasidone 160 mg/day is the most effective dose for overall psychotic symptoms and causes all-cause dropouts less than placebo. The high doses also possess higher efficacy for positive and depression symptoms without more severe weight gain and overall adverse effects than the lower doses. Two drawbacks of the higher doses appear to be the higher risks of somnolence and EPS. In addition, the higher doses give no more benefit for negative symptoms.

Lurasidone doses and effects may not be well correlated. It is not uncommon for a lower dose of an antipsychotic to cause more therapeutic or adverse effects. Despite its non-significance, a dose–response study found that risperidone 6 mg/day was superior to risperidone 10 mg/day in reducing overall psychotic symptoms^42^. Likewise, the present study found that lurasidone 80 mg/day significantly outperformed lurasidone 120 mg/day in reducing MADRS score. Together with the findings that lurasidone dosages was not correlated to D2 receptor occupancy^43^, the inconsistent orders of lurasidone doses and effects may need more investigation.

There were some limitations to this NMA. First, the present results should be viewed with caution because the confidence of all NMA estimates was low or very low. Second, some treatments were studied in a small number of patients. For example, the data of lurasidone 160 mg/day, the most effective and acceptable dose, was obtained from 121 adults with acute schizophrenia in a randomized trial only^34^. A type II error, therefore, could not be excluded in the comparison between this dose and others. Third, because the maximum effective doses of lurasidone may be higher than 160 mg/day, the data of dose–response relationships are still incomplete. A trial of lurasidone at the dose of greater than 160 mg/day will add more important evidence for lurasidone dosing. Last, only a few trials of other antipsychotics were included in this NMA. Therefore, we did not have sufficient evidence to compare the pharmacological effects of lurasidone and other antipsychotics.

In conclusions, each schizophrenia syndrome and lurasidone adverse effect has its pattern of doses and responses interaction. Lurasidone 160 mg/day is currently the most effective and acceptable dose for acute schizophrenia, but its maximal effective doses may be higher than 160 mg/day. A trial comparing lurasidone 80, 120, 160, and more than 160 mg/day is warranted.

## Supplementary Information

Supplementary Information

## Data Availability

The data and r code of this work are available at https://osf.io/qtn28/.
