# Supplementary material for: A network meta-analysis of the dose–response effects of lurasidone on acute schizophrenia
Source: Sci Rep. 2021 Mar 10;11:5571. doi: 10.1038/s41598-021-84836-z (PMC7946927; doi:10.1038/s41598-021-84836-z)
Supplement: Supplementary file 1 — Supplementary Information [file 41598_2021_84836_MOESM1_ESM.pdf]

A Network Meta-Analysis of the Dose-Response Effects of Lurasidone on Acute Schizophrenia

Manit Srisurapanont[1,2*], Sirijit Suttajit[1,2], Surinporn Likhitsathian[1], Benchalak Maneeton[1], Narong Maneeton[1]

[1] Department of Psychiatry, Faculty of Medicine, Chiang Mai University, Chiang Mai, Thailand
[2] These authors contributed equally: Manit Srisurapanont and Sirijit Suttajit

***Corresponding Author:**
Manit Srisurapanont (ORCID ID: 0000-0001-6203-1206)
Department of Psychiatry, Chiang Mai University Faculty of Medicine
110 Inthawarorot Road, Si Phum, Mueang, Chiang Mai 50200 THAILAND
Email: manit.s@cmu.ac.th

**STable 1:** PRISMA NMA checklist	Page 1-5
**STable 2:** Database searches	Page 6
**SFigure 1:** Effect-modifier plots for exploring the intransitivity	Page 7
**SFigure 2:** Risk of bias assessment	Page 8
**SFigure 3:** Forest plots of the treatment effects	Page 9
**STable 3:** League tables presenting network meta-analysis estimates (lower triangle) and direct estimates (upper triangle)	Page 10-13
**STable 4:** The analysis of Separate indirect from direct evidence (SIDE) (back-calculation method)	Page 14-21
**STable 5:** Rating the confidence of NMAs using CINeMA	Page 22-31
**SFigure 4:** Funnel plots for assessing publication bias	Page 32
**STable 6:** Confidence rating and effect estimates	Page 33-35

STable 1: PRISMA NMA Checklist

Section/Topic	Item #	Checklist Item	Reported on Page #
**TITLE**			
Title	1	Identify the report as a systematic review *incorporating a network meta-analysis (or related form of meta-analysis).*	***1***
**ABSTRACT**			
Structured summary	2	Provide a structured summary including, as applicable:<br>**Background:** main objectives<br>**Methods:** data sources; study eligibility criteria, participants, and interventions; study appraisal; and *synthesis methods, such as network meta-analysis.*<br>**Results:** number of studies and participants identified; summary estimates with corresponding confidence/credible intervals; *treatment rankings may also be discussed. Authors may choose to summarize pairwise comparisons against a chosen treatment included in their analyses for brevity.*<br>**Discussion/Conclusions:** limitations; conclusions and implications of findings.<br>**Other:** primary source of funding; systematic review registration number with registry name.	***2***
**INTRODUCTION**			
Rationale	3	Describe the rationale for the review in the context of what is already known, *including mention of why a network meta-analysis has been conducted.*	***3-4***
Objectives	4	Provide an explicit statement of questions being addressed, with reference to participants, interventions, comparisons, outcomes, and study design (PICOS).	***4***
**METHODS**			
Protocol and registration	5	Indicate whether a review protocol exists and if and where it can be accessed (e.g., Web address); and, if available, provide registration information, including registration number.	***4***
Eligibility criteria	6	Specify study characteristics (e.g., PICOS, length of follow-up) and report characteristics (e.g., years considered, language, publication status) used as criteria for eligibility, giving rationale. *Clearly describe eligible treatments included in the treatment network, and note whether any have been clustered or merged into the same node (with justification).*	***5***
Information sources	7	Describe all information sources (e.g., databases with dates of coverage, contact with study authors to identify additional studies) in the search and date last searched.	***5***
Search	8	Present full electronic search strategy for at least one	***5 and STable***

		database, including any limits used, such that it could be repeated.	***2***
Study selection	9	State the process for selecting studies (i.e., screening, eligibility, included in systematic review, and, if applicable, included in the meta-analysis).	***5***
Data collection process	10	Describe method of data extraction from reports (e.g., piloted forms, independently, in duplicate) and any processes for obtaining and confirming data from investigators.	***5***
Data items	11	List and define all variables for which data were sought (e.g., PICOS, funding sources) and any assumptions and simplifications made.	***6***
**Geometry of the network**	**S1**	Describe methods used to explore the geometry of the treatment network under study and potential biases related to it. This should include how the evidence base has been graphically summarized for presentation, and what characteristics were compiled and used to describe the evidence base to readers.	***6***
Risk of bias within individual studies	12	Describe methods used for assessing risk of bias of individual studies (including specification of whether this was done at the study or outcome level), and how this information is to be used in any data synthesis.	***6***
Summary measures	13	State the principal summary measures (e.g., risk ratio, difference in means). *Also describe the use of additional summary measures assessed, such as treatment rankings and surface under the cumulative ranking curve (SUCRA) values, as well as modified approaches used to present summary findings from meta-analyses.*	***7***
Planned methods of analysis	14	Describe the methods of handling data and combining results of studies for each network meta-analysis. This should include, but not be limited to:<br>• *Handling of multi-arm trials;*<br>• *Selection of variance structure;*<br>• *Selection of prior distributions in Bayesian analyses; and*<br>• *Assessment of model fit.*	***7***
**Assessment of Inconsistency**	**S2**	Describe the statistical methods used to evaluate the agreement of direct and indirect evidence in the treatment network(s) studied. Describe efforts taken to address its presence when found.	***7***
Risk of bias across studies	15	Specify any assessment of risk of bias that may affect the cumulative evidence (e.g., publication bias, selective reporting within studies).	***7-8***
Additional analyses	16	Describe methods of additional analyses if done, indicating which were pre-specified. This may include, but not be limited to, the following:<br>• Sensitivity or subgroup analyses;<br>• Meta-regression analyses;<br>• *Alternative formulations of the treatment network; and*<br>• *Use of alternative prior distributions for Bayesian analyses (if applicable).*	***7-8***

RESULTS†

Study selection	17	Give numbers of studies screened, assessed for eligibility, and included in the review, with reasons for exclusions at each stage, ideally with a flow diagram.	***9 and SFigure 1***
**Presentation of network structure**	**S3**	Provide a network graph of the included studies to enable visualization of the geometry of the treatment network.	***SFigure 2***
**Summary of network geometry**	**S4**	Provide a brief overview of characteristics of the treatment network. This may include commentary on the abundance of trials and randomized patients for the different interventions and pairwise comparisons in the network, gaps of evidence in the treatment network, and potential biases reflected by the network structure.	***9-10 and SFigure 2***
Study characteristics	18	For each study, present characteristics for which data were extracted (e.g., study size, PICOS, follow-up period) and provide the citations.	***10, Table 1, and SFigure 3***
Risk of bias within studies	19	Present data on risk of bias of each study and, if available, any outcome level assessment.	***10-11 and SFigure 4A***
Results of individual studies	20	For all outcomes considered (benefits or harms), present, for each study: 1) simple summary data for each intervention group, and 2) effect estimates and confidence intervals. *Modified approaches may be needed to deal with information from larger networks.*	***11 and Table 1***
Synthesis of results	21	Present results of each meta-analysis done, including confidence/credible intervals. *In larger networks, authors may focus on comparisons versus a particular comparator (e.g. placebo or standard care), with full findings presented in an appendix. League tables and forest plots may be considered to summarize pairwise comparisons.* If additional summary measures were explored (such as treatment rankings), these should also be presented.	***11-13 Figure 1, Table 2, and SFigure 5 and STable 3***
**Exploration for inconsistency**	**S5**	Describe results from investigations of inconsistency. This may include such information as measures of model fit to compare consistency and inconsistency models, *P* values from statistical tests, or summary of inconsistency estimates from different parts of the treatment network.	***13, Figure 1, SFigure 5, and STable 4-5***
Risk of bias across studies	22	Present results of any assessment of risk of bias across studies for the evidence base being studied.	***13 and SFigure 4B***
Results of additional analyses	23	Give results of additional analyses, if done (e.g., sensitivity or subgroup analyses, meta-regression analyses, *alternative network geometries studied, alternative choice of prior distributions for Bayesian analyses,* and so forth).	***13 and SFigure 6***

DISCUSSION

Summary of evidence	24	Summarize the main findings, including the strength of evidence for each main outcome; consider their relevance to key groups (e.g., healthcare providers, users, and policy-makers).	***15-18***

Limitations	25	Discuss limitations at study and outcome level (e.g., risk of bias), and at review level (e.g., incomplete retrieval of identified research, reporting bias). *Comment on the validity of the assumptions, such as transitivity and consistency. Comment on any concerns regarding network geometry (e.g., avoidance of certain comparisons).*	***18***
Conclusions	26	Provide a general interpretation of the results in the context of other evidence, and implications for future research.	***19***
**FUNDING**			
Funding	27	Describe sources of funding for the systematic review and other support (e.g., supply of data); role of funders for the systematic review. This should also include information regarding whether funding has been received from manufacturers of treatments in the network and/or whether some of the authors are content experts with professional conflicts of interest that could affect use of treatments in the network.	***24***

PICOS = population, intervention, comparators, outcomes, study design.
* Text in italics indicates wording specific to reporting of network meta-analyses that has been added to guidance from the PRISMA statement.
† Authors may wish to plan for use of appendices to present all relevant information in full detail for items in this section.

STable 2: Database searches and search terms

Pubmed<br>Search: (lurasidone) AND (schizophrenia) Filters: Randomized Controlled Trial
Scopus<br>(lurasidone) AND (schizophrenia) AND (LIMIT-TO (EXACTKEYWORD , "Randomized Controlled Trial"))
Web of Science<br>ALL=((lurasidone) AND (schizophrenia) AND (random*))
Cochrane Central Register of Controlled Trials<br>lurasidone AND schizophrenia AND random*
ClinicalTrials.gov<br>lurasidone | Completed Studies | Studies With Results | Interventional Studies | Schizophrenia | Adult

SFigure 1: Effect-modifier plots for exploring the intransitivity

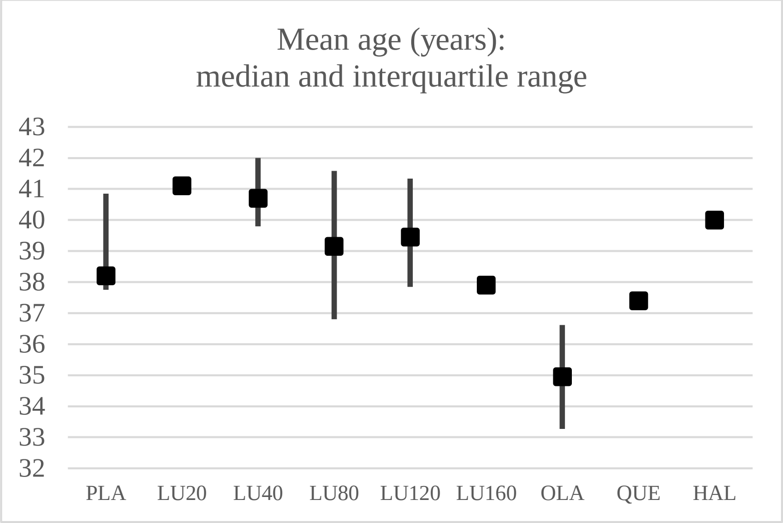

A: Mean ages among treatment groups: box and whisker chart for median and interquartile-range plots (data of risperidone group was not available).

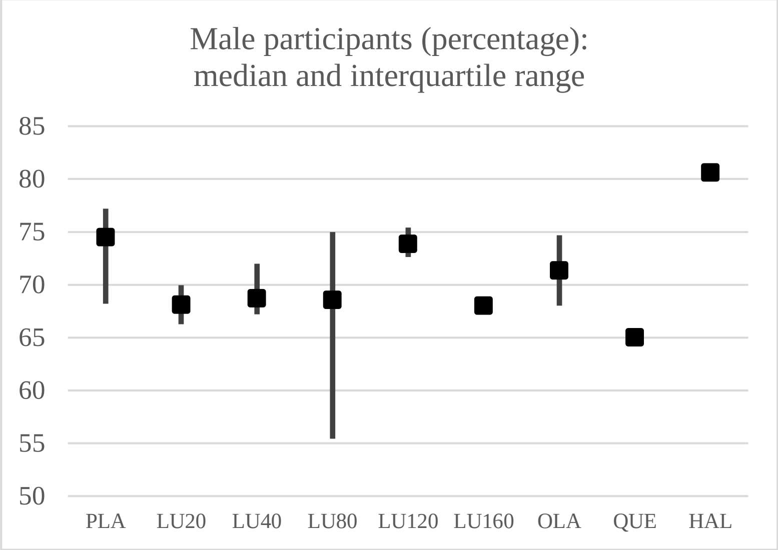

B: Percentages of male participants among treatment groups: box and whisker chart for median interquartile-range plots (data of risperidone group was not available).

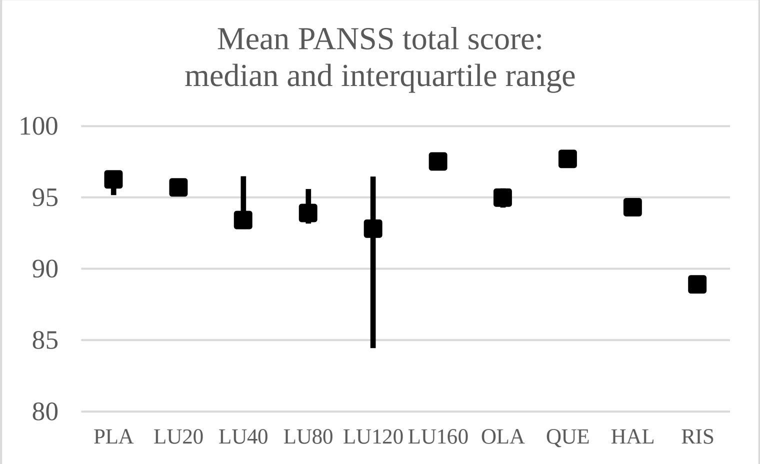

C: Percentages of male participants among treatment groups: box and whisker chart for median interquartile-range plots.

SFigure 2: Risk of bias assessment

A: Risk of bias in individual trials.

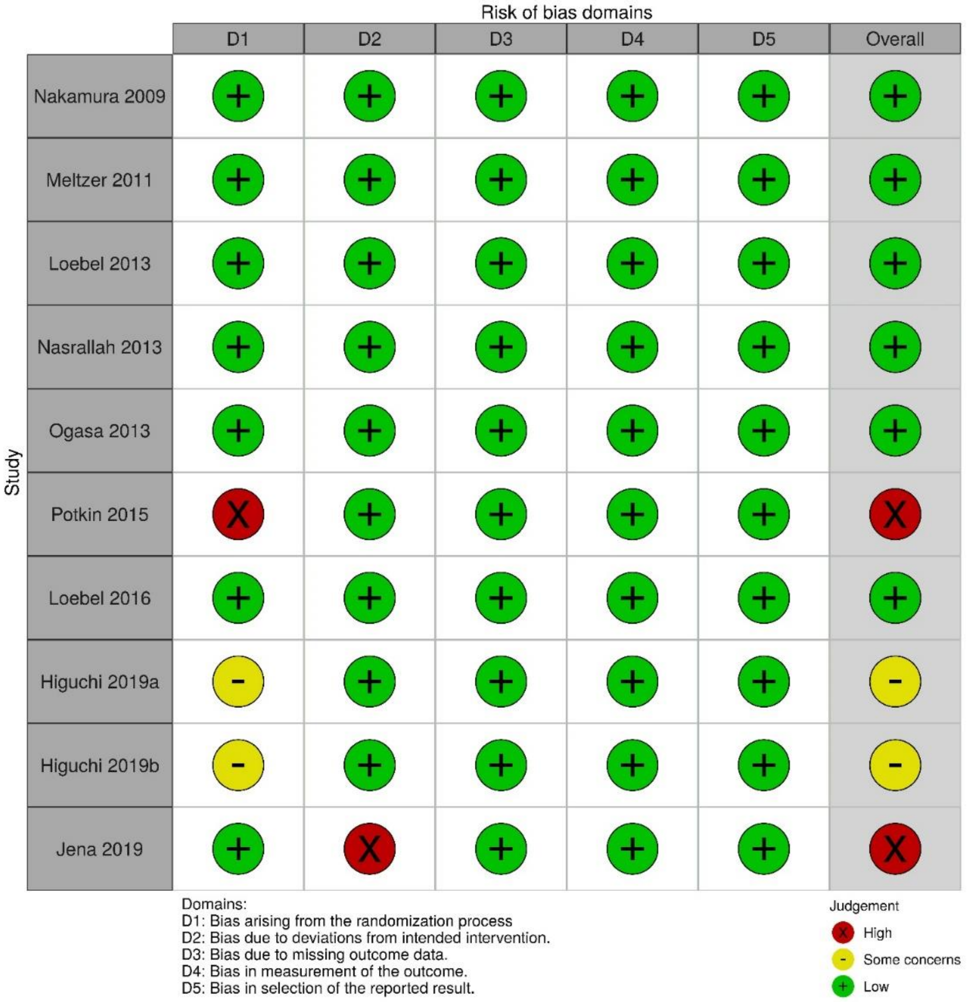

B: Risk of bias across included trials weighted by sample sizes.

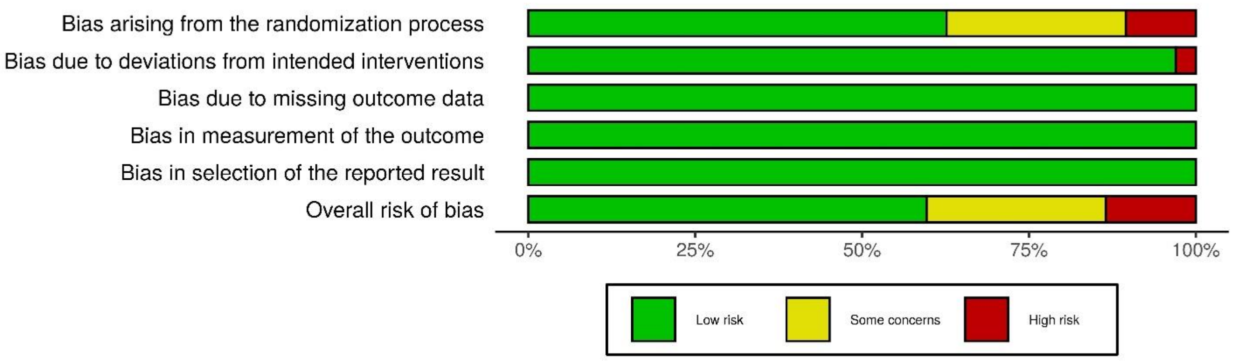

SFigure 3: Forest plots of the treatment effects: five different doses of lurasidone and other antipsychotics compared with placebo

Note: Forest plots of primary outcomes (i.e. PANSS total score reduction and all-cause dropout rates) are parts of the main manuscript.

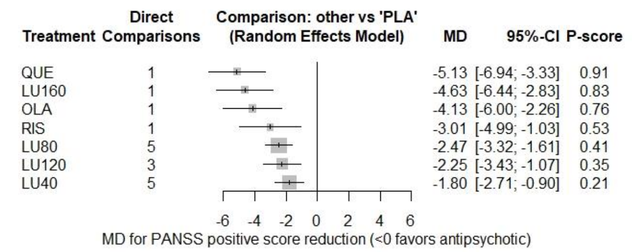

A: PANSS positive score reduction
(Heterogeneity:χ2=15.62, df=11, p=0.16)

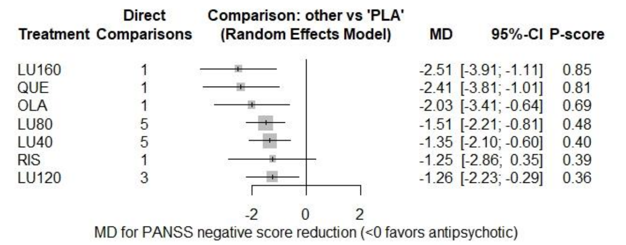

B: PANSS negative score reduction
(Heterogeneity:χ2=16.95, df=11, p=0.11)

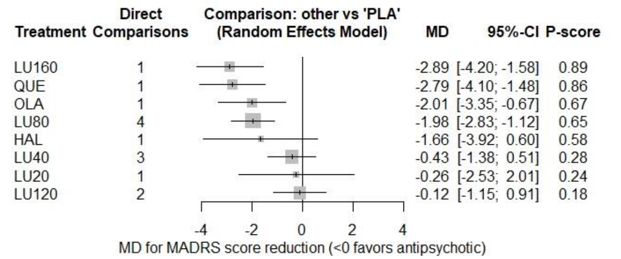

C: MADRS score reduction
(Heterogeneity:χ2=6.25, df=6, p=0.40)

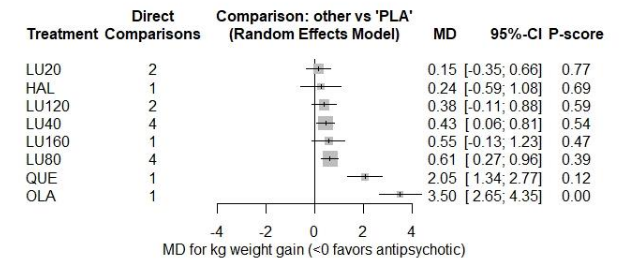

D: Weight gain
(Heterogeneity:χ2=3.68, df=8, p= 0.88)

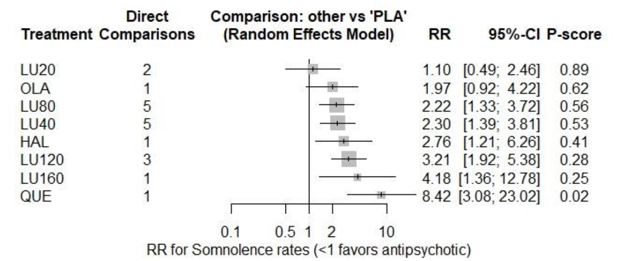

E: Somnolence rates
(Heterogeneity:χ2=6.18, d=11, p=0.86)

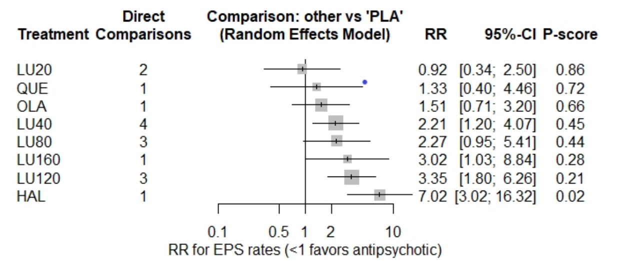

F: EPS rates
(Heterogeneity:χ2=9.28, d=9, p=0.41)

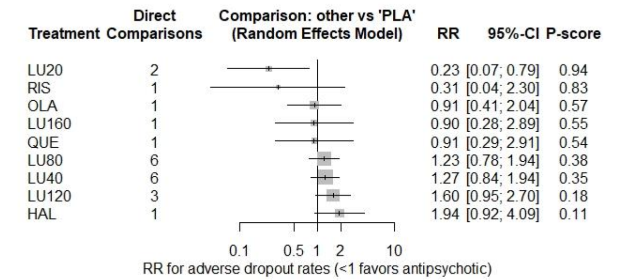

G: Adverse dropout rates
(Heterogeneity: χ2=9.43, d=13, p=0.74).

STable 3: League tables presenting network meta-analysis estimates (lower triangle) and direct estimates (upper triangle): secondary efficacy (A-C) and adverse effects (D-F) of lurasidone and placebo for schizophrenia.

Note: The league tables of primary outcomes (i.e. PANSS total score reduction and all-cause dropout rates) are parts of the main manuscript.

A: MDs for PANSS positive score reduction (95% CIs)[a]

Quetiapine 600 mg/day	-0.50 (-2.53 to 1.53)	-	-	-2.00 (-4.03 to 0.03)	-	-	**-5.80 (-7.83 to -3.77)**
-0.50 (-2.53 to 1.53)	***Lurasidone 160 mg/day***	-	-	-1.50 (-3.53 to 0.53)	-	-	**-5.30 (-7.33 to -3.27)**
-1.00 (-3.56 to 1.56)	-0.50 (-3.06 to 2.06)	***Olanzapine 10-15 mg/day***	-	-6.85 (-13.96 to 0.26)	-1.80 (-4.06 to 0.46)	-1.60 (-3.86 to 0.66)	**-3.90 (-6.16 to -1.64)**
-2.12 (-4.74 to 0.49)	-1.62 (-4.24 to 0.99)	-1.12 (-3.78 to 1.53)	***Risperidone 4 mg/day***	-1.50 (-3.68 to 0.68)	-	-0.90 (-3.18 to 1.38)	**-2.30 (-4.48 to -0.12)**
**-2.67 (-4.47 to -0.86)**	**-2.17 (-3.97 to -0.36)**	-1.67 (-3.62 to 0.29)	-0.54 (-2.54 to 1.45)	***Lurasidone 80 mg/day***	-1.10 (-3.13 to 0.93)	-0.72 (-1.84 to 0.40)	**-2.42 (-3.30 to -1.53)**
**-2.88 (-4.98 to -0.78)**	**-2.38 (-4.48 to -0.28)**	-1.88 (-3.82 to 0.05)	-0.76 (-2.98 to 1.46)	-0.22 (-1.50 to 1.07)	***Lurasidone 120 mg/day***	-0.47 (-1.77 to 0.82)	**-2.46 (-3.75 to -1.17)**
**-3.33 (-5.28 to -1.38)**	**-2.83 (-4.78 to -0.88)**	**-2.33 (-4.21 to -0.45)**	-1.21 (-3.23 to 0.81)	-0.66 (-1.65 to 0.33)	-0.45 (-1.64 to 0.75)	***Lurasidone 40 mg/day***	**-1.68 (-2.61 to -0.75)**
**-5.13 (-6.94 to -3.33)**	**-4.63 (-6.44 to -2.83)**	**-4.13 (-6.00 to -2.26)**	**-3.01 (-4.99 to -1.03)**	**-2.47 (-3.32 to -1.61)**	**-2.25 (-3.43 to -1.07)**	**-1.80 (-2.71 to -0.90)**	***Placebo***

B: MDs for PANSS negative score reduction (95% CIs)[a]

Lurasidone 160 mg/day	-0.10 (-1.62 to 1.42)	-	-0.40 (-1.92 to 1.12)	-	-	-	**-3.30 (-4.93 to -1.67)**
-0.10 (-1.62 to 1.42)	***Quetiapine 600 mg/day***	-	-0.30 (-1.82 to 1.22)	-	-	-	**-3.20 (-4.83 to -1.57)**
-0.49 (-2.40 to 1.43)	-0.39 (-2.30 to 1.53)	***Olanzapine 10-15 mg/day***	0.03 (-3.31 to 3.37)	-0.20 (-1.93 to 1.53)	-	-1.00 (-2.85 to 0.85)	**-2.60 (-4.33 to -0.87)**
-1.01 (-2.38 to 0.37)	-0.91 (-2.28 to 0.47)	-0.52 (-1.95 to 0.91)	***Lurasidone 80 mg/day***	-0.01 (-0.94 to 0.92)	0.00 (-1.85 to 1.85)	-0.40 (-1.92 to 1.12)	**-1.34 (-2.07 to -0.61)**
-1.17 (-2.68 to 0.35)	-1.07 (-2.58 to 0.45)	-0.68 (-2.07 to 0.71)	-0.16 (-0.97 to 0.65)	***Lurasidone 40 mg/day***	0.10 (-1.75 to 1.95)	-0.07 (-1.13 to 0.99)	**-1.18 (-1.96 to -0.40)**
-1.26 (-3.33 to 0.81)	-1.16 (-3.23 to 0.91)	-0.77 (-2.82 to 1.27)	-0.26 (-1.87 to 1.36)	-0.10 (-1.72 to 1.53)	***Risperidone 4 mg/day***	-	-0.80 (-2.65 to 1.05)
-1.25 (-2.90 to 0.39)	-1.15 (-2.80 to 0.49)	-0.77 (-2.24 to 0.71)	-0.25 (-1.29 to 0.79)	-0.09 (-1.07 to 0.90)	0.01 (-1.79 to 1.80)	***Lurasidone 120 mg/day***	**-1.18 (-2.24 to -0.12)**
**-2.51 (-3.91 to -1.11)**	**-2.41 (-3.81 to -1.01)**	**-2.03 (-3.41 to -0.64)**	**-1.51 (-2.21 to -0.81)**	**-1.35 (-2.10 to -0.60)**	-1.25 (-2.86 to 0.35)	**-1.26 (-2.23 to -0.29)**	***Placebo***

C: MDs for MADRS score reduction (95% CI)[a]

Lurasidone 160 mg/day	-0.10 (-1.53 to 1.33)	-	-0.40 (-1.83 to 1.03)	-	-	-	-	-3.40 (-4.83 to -1.97)
-0.10 (-1.53 to 1.33)	***Quetiapine 600*** *mg/day*	-	-0.30 (-1.73 to 1.13)	-	-	-	-	-3.30 (-4.73 to -1.87)
-0.88 (-2.71 to 0.94)	-0.78 (-2.61 to 1.04)	***Olanzapine 10-15 mg/day***	-	-	-1.50 (-2.93 to -0.07)	-	-1.80 (-3.37 to -0.23)	-2.20 (-3.77 to -0.63)
-0.91 (-2.22 to 0.40)	-0.81 (-2.12 to 0.50)	-0.03 (-1.50 to 1.44)	***Lurasidone 80 mg/day***	0.20 (-2.51 to 2.91)	-1.08 (-2.36 to 0.19)	-1.20 (-3.93 to 1.53)	-1.40 (-2.83 to 0.03)	-1.92 (-2.81 to -1.04)
-1.23 (-3.77 to 1.32)	-1.13 (-3.67 to 1.42)	-0.35 (-2.89 to 2.19)	-0.32 (-2.59 to 1.96)	***Haloperidol 10*** **mg/day**	-1.60 (-4.34 to 1.14)	-1.40 (-4.10 to 1.30)	-	-0.80 (-3.50 to 1.90)
**-2.45 (-3.99 to -0.92)**	**-2.35 (-3.89 to -0.82)**	**-1.57 (-2.88 to -0.27)**	**-1.54 (-2.60 to -0.49)**	-1.23 (-3.51 to 1.06)	***Lurasidone 40 mg/day***	0.20 (-2.55 to 2.95)	-0.35 (-1.41 to 0.70)	-0.13 (-1.11 to 0.86)
**-2.63 (-5.19 to -0.07)**	-2.53 (-5.09 to 0.03)	-1.75 (-4.30 to 0.81)	-1.72 (-4.01 to 0.57)	-1.40 (-4.10 to 1.30)	-0.17 (-2.48 to 2.13)	***Lurasidone 20 mg/day***	-	0.60 (-2.11 to 3.31)
**-2.77 (-4.36 to -1.18)**	**-2.67 (-4.26 to -1.08)**	**-1.89 (-3.26 to -0.52)**	**-1.86 (-2.99 to -0.73)**	-1.54 (-3.93 to 0.85)	-0.31 (-1.35 to 0.72)	-0.14 (-2.54 to 2.26)	***Lurasidone 120 mg/day***	0.13 (-0.97 to 1.22)
**-2.89 (-4.20 to -1.58)**	**-2.79 (-4.10 to -1.48)**	**-2.01 (-3.35 to -0.67)**	**-1.98 (-2.83 to -1.12)**	-1.66 (-3.92 to 0.60)	-0.43 (-1.38 to 0.51)	-0.26 (-2.53 to 2.01)	-0.12 (-1.15 to 0.91)	***Placebo***

D: MDs for weight gain (95% CI)[b]

Placebo	-0.16 (-0.70 to 0.37)	-0.00 (-1.00 to 1.00)	-0.35 (-0.90 to 0.20)	**-0.43 (-0.82 to -0.04)**	-0.50 (-1.23 to 0.23)	**-0.61 (-0.96 to -0.26)**	**-2.00 (-2.77 to -1.23)**	**-3.50 (-4.41 to -2.59)**
-0.15 (-0.66 to 0.35)	***Lurasidone 20 mg/day***	-0.30 (-1.24 to 0.64)	-	-0.20 (-1.25 to 0.85)	-	-1.10 (-2.25 to 0.05)	-	-
-0.24 (-1.08 to 0.59)	-0.09 (-0.96 to 0.77)	***Haloperidol 10 mg/day***	-	0.10 (-1.02 to 1.22)	-	-0.80 (-2.01 to 0.41)	-	-
-0.38 (-0.88 to 0.11)	-0.23 (-0.92 to 0.46)	-0.14 (-1.09 to 0.82)	***Lurasidone 120 mg/day***	-0.03 (-0.57 to 0.51)	-	-	-	**-3.10 (-3.96 to -2.24)**
**-0.43 (-0.81 to -0.06)**	-0.28 (-0.87 to 0.31)	-0.19 (-1.06 to 0.68)	-0.05 (-0.54 to 0.44)	***Lurasidone 40 mg/day***	-	-0.22 (-0.74 to 0.31)	-	**-3.10 (-4.02 to -2.18)**
-0.55 (-1.23 to 0.13)	-0.40 (-1.24 to 0.44)	-0.31 (-1.38 to 0.76)	-0.17 (-1.00 to 0.66)	-0.12 (-0.88 to 0.63)	***Lurasidone 160 mg/day***	0.00 (-0.74 to 0.74)	**-1.50 (-2.34 to -0.66)**	-
**-0.61 (-0.96 to -0.27)**	-0.46 (-1.04 to 0.12)	-0.37 (-1.24 to 0.50)	-0.23 (-0.81 to 0.34)	-0.18 (-0.62 to 0.26)	-0.06 (-0.74 to 0.62)	***Lurasidone 80 mg/day***	**-1.50 (-2.28 to -0.72)**	-
**-2.05 (-2.77 to -1.34)**	**-1.90 (-2.77 to -1.04)**	**-1.81 (-2.90 to -0.72)**	**-1.67 (-2.53 to -0.81)**	**-1.62 (-2.41 to -0.83)**	**-1.50 (-2.34 to -0.66)**	**-1.44 (-2.16 to -0.72)**	***Quetiapine 600 mg/day***	-
**-3.50 (-4.35 to -2.65)**	**-3.35 (-4.33 to -2.37)**	**-3.26 (-4.44 to -2.08)**	**-3.12 (-3.97 to -2.27)**	**-3.07 (-3.92 to -2.21)**	**-2.95 (-4.03 to -1.86)**	**-2.89 (-3.79 to -1.99)**	**-1.45 (-2.55 to -0.34)**	***Olanzapine 10-15 mg/day***

E: RR for somnolence rates

Placebo	1.04 (0.43 to 2.50)	0.48 (0.17 to 1.34)	**0.42 (0.24 to 0.74)**	**0.49 (0.28 to 0.84)**	0.44 (0.14 to 1.38)	**0.34 (0.19 to 0.61)**	**0.12 (0.02 to 0.98)**	**0.06 (0.01 to 0.46)**
0.91 (0.41 to 2.03)	***Lurasidone 20 mg/day***	-	0.50 (0.16 to 1.59)	0.94 (0.25 to 3.62)	0.45 (0.15 to 1.40)	-	-	-
0.51 (0.24 to 1.08)	0.56 (0.19 to 1.61)	***Olanzapine 10-15 mg/day***	-	0.89 (0.41 to 1.95)	-	0.59 (0.29 to 1.20)	-	-
**0.45 (0.27 to 0.75)**	0.50 (0.21 to 1.16)	0.89 (0.40 to 1.94)	***Lurasidone 80 mg/day***	0.92 (0.53 to 1.60)	0.90 (0.37 to 2.20)	0.67 (0.34 to 1.34)	0.60 (0.20 to 1.80)	**0.30 (0.11 to 0.79)**
**0.44 (0.26 to 0.72)**	0.48 (0.20 to 1.15)	0.86 (0.43 to 1.72)	0.97 (0.59 to 1.59)	***Lurasidone 40 mg/day***	0.48 (0.15 to 1.48)	0.70 (0.45 to 1.10)	-	-
**0.36 (0.16 to 0.82)**	0.40 (0.15 to 1.07)	0.72 (0.25 to 2.01)	0.81 (0.37 to 1.78)	0.83 (0.36 to 1.91)	***Haloperidol 10 mg/day***	-	-	-
**0.31 (0.19 to 0.52)**	**0.34 (0.14 to 0.84)**	0.61 (0.31 to 1.20)	0.69 (0.41 to 1.17)	0.72 (0.47 to 1.10)	0.86 (0.36 to 2.03)	***Lurasidone 120 mg/day***	-	-
**0.24 (0.08 to 0.73)**	**0.26 (0.07 to 0.99)**	0.47 (0.13 to 1.70)	0.53 (0.19 to 1.50)	0.55 (0.18 to 1.70)	0.66 (0.18 to 2.40)	0.77 (0.25 to 2.41)	***Lurasidone 160 mg/day***	0.50 (0.22 to 1.11)
**0.12 (0.04 to 0.32)**	**0.13 (0.04 to 0.45)**	**0.23 (0.07 to 0.77)**	**0.26 (0.11 to 0.66)**	**0.27 (0.10 to 0.76)**	0.33 (0.10 to 1.08)	0.38 (0.14 to 1.07)	0.50 (0.22 to 1.11)	***Quetiapine 600 mg/day***

F: RR for EPS rates[c]

Placebo	1.24 (0.43 to 3.56)	0.14 (0.02 to 1.14)	0.61 (0.25 to 1.44)	**0.50 (0.27 to 0.94)**	0.52 (0.19 to 1.41)	**0.06 (0.01 to 0.47)**	**0.32 (0.16 to 0.64)**	**0.20 (0.06 to 0.68)**
1.09 (0.40 to 2.98)	***Lurasidone 20 mg/day***	-	-	0.47 (0.09 to 2.54)	0.50 (0.09 to 2.69)	-	-	**0.14 (0.03 to 0.58)**
0.75 (0.22 to 2.52)	0.69 (0.16 to 3.03)	***Quetiapine 600 mg/day***	-	-	0.52 (0.21 to 1.29)	0.44 (0.18 to 1.07)	-	-
0.66 (0.31 to 1.41)	0.61 (0.18 to 2.02)	0.88 (0.24 to 3.29)	***Olanzapine 10-15 mg/day***	0.80 (0.40 to 1.63)	0.20 (0.01 to 4.03)	-	**0.44 (0.23 to 0.82)**	-
**0.45 (0.25 to 0.84)**	0.42 (0.14 to 1.25)	0.60 (0.18 to 2.05)	0.68 (0.35 to 1.32)	***Lurasidone 40 mg/day***	1.63 (0.58 to 4.62)	-	0.67 (0.40 to 1.12)	**0.29 (0.10 to 0.85)**
0.44 (0.19 to 1.05)	0.40 (0.12 to 1.36)	0.59 (0.24 to 1.44)	0.66 (0.24 to 1.81)	0.97 (0.41 to 2.34)	***Lurasidone 80 mg/day***	0.85 (0.41 to 1.74)	0.67 (0.11 to 4.02)	**0.27 (0.09 to 0.80)**
**0.33 (0.11 to 0.97)**	0.30 (0.08 to 1.20)	0.44 (0.18 to 1.07)	0.50 (0.15 to 1.64)	0.73 (0.25 to 2.18)	0.75 (0.37 to 1.52)	***Lurasidone 160 mg/day***	-	-
**0.30 (0.16 to 0.56)**	**0.27 (0.09 to 0.84)**	0.40 (0.11 to 1.39)	**0.45 (0.24 to 0.83)**	0.66 (0.40 to 1.09)	0.68 (0.27 to 1.70)	0.90 (0.29 to 2.78)	***Lurasidone 120 mg/day***	-
**0.14 (0.06 to 0.33)**	**0.13 (0.04 to 0.40)**	**0.19 (0.05 to 0.67)**	**0.21 (0.08 to 0.58)**	**0.31 (0.13 to 0.73)**	**0.32 (0.13 to 0.81)**	0.43 (0.14 to 1.34)	0.48 (0.19 to 1.19)	***Haloperidol 10 mg/day***

G: RR for adverse dropout rates (95% CIs)[c]

Lurasidone 20 mg/day	-	-	**0.27 (0.08 to 0.94)**	-	-	0.14 (0.02 to 1.13)	**0.12 (0.02 to 0.92)**	-	**0.09 (0.01 to 0.70)**
0.76 (0.07 to 7.77)	***Risperidone 4 mg/day***	-	0.25 (0.03 to 1.97)	-	-	0.34 (0.04 to 2.73)	0.24 (0.03 to 1.91)	-	-
0.26 (0.06 to 1.09)	0.34 (0.04 to 2.87)	***Olanzapine 10-15 mg/day***	0.75 (0.31 to 1.85)	-	-	-	0.98 (0.38 to 2.52)	0.55 (0.24 to 1.27)	-
**0.23 (0.07 to 0.79)**	0.31 (0.04 to 2.30)	0.91 (0.41 to 2.04)	***Placebo***	1.24 (0.34 to 4.51)	1.23 (0.34 to 4.47)	0.81 (0.50 to 1.33)	0.80 (0.52 to 1.24)	0.57 (0.31 to 1.06)	0.36 (0.12 to 1.09)
0.26 (0.05 to 1.38)	0.34 (0.03 to 3.41)	1.01 (0.25 to 4.07)	1.11 (0.35 to 3.53)	***Lurasidone 160 mg/day***	0.99 (0.25 to 3.87)	0.83 (0.23 to 3.00)	-	-	-
0.26 (0.05 to 1.37)	0.34 (0.03 to 3.38)	1.00 (0.25 to 4.04)	1.10 (0.34 to 3.51)	0.99 (0.25 to 3.87)	***Quetiapine 600 mg/day***	0.83 (0.23 to 3.03)	-	-	-
**0.19 (0.05 to 0.68)**	0.25 (0.03 to 1.89)	0.75 (0.31 to 1.77)	0.82 (0.52 to 1.29)	0.74 (0.23 to 2.36)	0.74 (0.23 to 2.38)	***Lurasidone 80 mg/day***	0.87 (0.52 to 1.45)	1.15 (0.43 to 3.08)	0.65 (0.27 to 1.57)
**0.18 (0.05 to 0.65)**	0.24 (0.03 to 1.80)	0.72 (0.32 to 1.61)	0.79 (0.52 to 1.20)	0.71 (0.21 to 2.36)	0.72 (0.22 to 2.38)	0.96 (0.61 to 1.53)	***Lurasidone 40 mg/day***	0.70 (0.40 to 1.23)	0.78 (0.33 to 1.82)
**0.15 (0.04 to 0.54)**	0.19 (0.03 to 1.49)	0.57 (0.26 to 1.26)	0.63 (0.37 to 1.05)	0.57 (0.16 to 1.96)	0.57 (0.16 to 1.98)	0.77 (0.43 to 1.37)	0.80 (0.48 to 1.32)	***Lurasidone 120 mg/day***	-
**0.12 (0.03 to 0.47)**	0.16 (0.02 to 1.31)	0.47 (0.17 to 1.34)	0.52 (0.24 to 1.09)	0.47 (0.12 to 1.78)	0.47 (0.12 to 1.79)	0.63 (0.30 to 1.32)	0.66 (0.32 to 1.35)	0.82 (0.36 to 1.90)	***Haloperidol 10 mg/day***

[a] A mean difference (MD) less than 0 indicates the superiority of treatment defined in the column over the other treatment defined in the row.
[b] A mean difference (MD) less than 0 indicates the more weight gain of treatment defined in the column over the other treatment defined in the row.
[c] An odd ratio (OR) less than 1 indicates the fewer events of treatment defined in the column over the other treatment defined in the row.
- indicates the nonavilability of direct estimate.

Treatments are reported in order of ranking of efficacy. Comparison treatments should be read from left to right, and the MD or OR is in the cell in common between the column-defining treatment and the row-defining treatment. The bold estimates indicate significant differences between the paired treatments.

ARI=aripiprazole; HAL=haloperidol; OLA=olanzapine; PAL=paliperidone extended-release; QUE=quetiapine; and RIS=risperidone.

STable 4: The analysis of Separate indirect from direct evidence (SIDE) (back-calculation method)

A. PANSS total score

comparison	k	prop	nma	95%-CI	direct	95%-CI	indir.	95%-CI	Diff	95%-CI	z	p-value
HAL:LU120	0	0	-3.13	[-10.91; 4.65]	.	.	-3.13	[-10.91; 4.65]	.	.	.	.
HAL:LU160	0	0	4.50	[-4.78; 13.78]	.	.	4.50	[-4.78; 13.78]	.	.	.	.
HAL:LU20	1	0.84	-7.75	[-15.37; -0.13]	-8.90	[-17.23; -0.57]	-1.86	[-20.69; 16.97]	-7.04	[-27.63; 13.55]	-0.67	0.5028
HAL:LU40	1	0.69	-4.33	[-11.34; 2.68]	-8.80	[-17.24; -0.36]	5.65	[-6.96; 18.25]	-14.45	[-29.61; 0.72]	-1.87	0.0620
HAL:LU80	1	0.70	-2.54	[-9.52; 4.44]	-2.40	[-10.73; 5.93]	-2.87	[-15.64; 9.90]	0.47	[-14.78; 15.72]	0.06	0.9516
HAL:OLA	0	0	2.17	[-6.64; 10.98]	.	.	2.17	[-6.64; 10.98]	.	.	.	.
HAL:PLA	1	0.68	-8.96	[-15.83; -2.09]	-3.70	[-12.03; 4.63]	-20.16	[-32.31; -8.01]	16.46	[1.72; 31.19]	2.19	0.0286
HAL:QUE	0	0	5.80	[-3.48; 15.08]	.	.	5.80	[-3.48; 15.08]	.	.	.	.
HAL:RIS	0	0	-2.46	[-11.97; 7.04]	.	.	-2.46	[-11.97; 7.04]	.	.	.	.
LU120:LU160	0	0	7.63	[0.07; 15.18]	.	.	7.63	[0.07; 15.18]	.	.	.	.
LU120:LU20	0	0	-4.62	[-11.05; 1.81]	.	.	-4.62	[-11.05; 1.81]	.	.	.	.
LU120:LU40	3	0.84	-1.20	[-5.42; 3.02]	-0.55	[-5.15; 4.05]	-4.73	[-15.42; 5.95]	4.19	[-7.45; 15.82]	0.71	0.4807
LU120:LU80	1	0.38	0.59	[-3.93; 5.10]	2.90	[-4.43; 10.23]	-0.83	[-6.57; 4.91]	3.73	[-5.57; 13.04]	0.79	0.4318
LU120:OLA	1	0.66	5.30	[-0.99; 11.58]	5.10	[-2.62; 12.82]	5.69	[-5.15; 16.52]	-0.59	[-13.89; 12.72]	-0.09	0.9313
LU120:PLA	3	0.82	-5.83	[-10.03; -1.64]	-6.77	[-11.41; -2.12]	-1.72	[-11.47; 8.03]	-5.05	[-15.85; 5.75]	-0.92	0.3595
LU120:QUE	0	0	8.93	[1.37; 16.48]	.	.	8.93	[1.37; 16.48]	.	.	.	.
LU120:RIS	0	0	0.67	[-7.12; 8.45]	.	.	0.67	[-7.12; 8.45]	.	.	.	.
LU160:LU20	0	0	-12.25	[-20.40; -4.09]	.	.	-12.25	[-20.40; -4.09]	.	.	.	.
LU160:LU40	0	0	-8.83	[-15.77; -1.88]	.	.	-8.83	[-15.77; -1.88]	.	.	.	.
LU160:LU80	1	0.79	-7.04	[-13.55; -0.53]	-4.30	[-11.63; 3.03]	-17.31	[-31.50; -3.13]	13.01	[-2.95; 28.98]	1.60	0.1101
LU160:OLA	0	0	-2.33	[-10.93; 6.27]	.	.	-2.33	[-10.93; 6.27]	.	.	.	.
LU160:PLA	1	0.79	-13.46	[-19.97; -6.95]	-16.20	[-23.53; -8.87]	-3.19	[-17.37; 11.00]	-13.01	[-28.98; 2.95]	-1.60	0.1101
LU160:QUE	1	1.00	1.30	[-6.03; 8.63]	1.30	[-6.03; 8.63]	.	.	.	.	.	.
LU160:RIS	0	0	-6.96	[-16.25; 2.32]	.	.	-6.96	[-16.25; 2.32]	.	.	.	.
LU20:LU40	1	0.44	3.42	[-2.16; 8.99]	0.10	[-8.34; 8.54]	5.99	[-1.44; 13.42]	-5.89	[-17.13; 5.35]	-1.03	0.3045
LU20:LU80	1	0.44	5.21	[-0.31; 10.73]	6.50	[-1.83; 14.83]	4.19	[-3.18; 11.57]	2.31	[-8.82; 13.43]	0.41	0.6845
LU20:OLA	0	0	9.92	[2.27; 17.56]	.	.	9.92	[2.27; 17.56]	.	.	.	.
LU20:PLA	2	0.84	-1.21	[-6.35; 3.92]	0.64	[-4.95; 6.22]	-11.22	[-24.22; 1.78]	11.86	[-2.30; 26.01]	1.64	0.1006
LU20:QUE	0	0	13.55	[5.39; 21.70]	.	.	13.55	[5.39; 21.70]	.	.	.	.
LU20:RIS	0	0	5.28	[-3.16; 13.73]	.	.	5.28	[-3.16; 13.73]	.	.	.	.
LU40:LU80	4	0.79	1.79	[-1.53; 5.11]	1.78	[-1.95; 5.51]	1.83	[-5.45; 9.11]	-0.05	[-8.23; 8.13]	-0.01	0.9906
LU40:OLA	1	0.61	6.50	[0.56; 12.44]	3.00	[-4.62; 10.62]	11.94	[2.44; 21.43]	-8.94	[-21.11; 3.24]	-1.44	0.1502
LU40:PLA	6	0.92	-4.63	[-7.67; -1.59]	-3.80	[-6.96; -0.64]	-14.66	[-25.63; -3.68]	10.86	[-0.56; 22.28]	1.86	0.0625
LU40:QUE	0	0	10.13	[3.18; 17.07]	.	.	10.13	[3.18; 17.07]	.	.	.	.
LU40:RIS	1	0.77	1.87	[-5.12; 8.85]	1.00	[-6.96; 8.96]	4.78	[-9.82; 19.38]	-3.78	[-20.41; 12.85]	-0.45	0.6558
LU80:OLA	1	0.22	4.71	[-1.36; 10.78]	8.00	[-4.94; 20.94]	3.78	[-3.09; 10.65]	4.22	[-10.43; 18.87]	0.56	0.5726
LU80:PLA	6	0.90	-6.42	[-9.33; -3.51]	-5.74	[-8.80; -2.68]	-12.73	[-22.06; -3.40]	6.99	[-2.83; 16.81]	1.40	0.1630
LU80:QUE	1	0.79	8.34	[1.83; 14.85]	5.60	[-1.73; 12.93]	18.61	[4.43; 32.80]	-13.01	[-28.98; 2.95]	-1.60	0.1101
LU80:RIS	1	0.78	0.08	[-6.88; 7.03]	2.80	[-5.07; 10.67]	-9.60	[-24.44; 5.25]	12.40	[-4.41; 29.20]	1.45	0.1482
OLA:PLA	1	0.59	-11.13	[-17.04; -5.22]	-12.70	[-20.42; -4.98]	-8.91	[-18.09; 0.26]	-3.79	[-15.77; 8.20]	-0.62	0.5359
OLA:QUE	0	0	3.63	[-4.97; 12.23]	.	.	3.63	[-4.97; 12.23]	.	.	.	.
OLA:RIS	0	0	-4.63	[-13.44; 4.18]	.	.	-4.63	[-13.44; 4.18]	.	.	.	.
QUE:PLA	1	0.79	-14.76	[-21.27; -8.25]	-17.50	[-24.83; -10.17]	-4.49	[-18.67; 9.70]	-13.01	[-28.98; 2.95]	-1.60	0.1101
RIS:PLA	1	0.77	-6.50	[-13.41; 0.42]	-4.60	[-12.47; 3.27]	-12.90	[-27.36; 1.56]	8.30	[-8.16; 24.76]	0.99	0.3230
QUE:RIS	0	0	-8.26	[-17.55; 1.02]	.	.	-8.26	[-17.55; 1.02]	.	.	.	.

B. PANSS positive score

comparison	k	prop	nma	95%-CI	direct	95%-CI	indir.	95%-CI	Diff	95%-CI	z	p-value
LU120:LU160	0	0	2.38	[0.28; 4.48]	.	.	2.38	[0.28; 4.48]	.	.	.	.
LU120:LU40	3	0.85	-0.45	[-1.64; 0.75]	-0.47	[-1.77; 0.82]	-0.30	[-3.43; 2.83]	-0.18	[-3.56; 3.21]	-0.10	0.9187
LU120:LU80	1	0.40	0.22	[-1.07; 1.50]	1.10	[-0.93; 3.13]	-0.38	[-2.05; 1.28]	1.48	[-1.14; 4.11]	1.11	0.2679
LU120:OLA	1	0.73	1.88	[-0.05; 3.82]	1.80	[-0.46; 4.06]	2.11	[-1.65; 5.87]	-0.31	[-4.70; 4.08]	-0.14	0.8896
LU120:PLA	3	0.83	-2.25	[-3.43; -1.07]	-2.46	[-3.75; -1.17]	-1.22	[-4.09; 1.65]	-1.24	[-4.39; 1.91]	-0.77	0.4391
LU120:QUE	0	0	2.88	[0.78; 4.98]	.	.	2.88	[0.78; 4.98]	.	.	.	.
LU120:RIS	0	0	0.76	[-1.46; 2.98]	.	.	0.76	[-1.46; 2.98]	.	.	.	.
LU160:LU40	0	0	-2.83	[-4.78; -0.88]	.	.	-2.83	[-4.78; -0.88]	.	.	.	.
LU160:LU80	1	0.79	-2.17	[-3.97; -0.36]	-1.50	[-3.53; 0.53]	-4.74	[-8.72; -0.76]	3.24	[-1.23; 7.71]	1.42	0.1550
LU160:OLA	0	0	-0.50	[-3.06; 2.06]	.	.	-0.50	[-3.06; 2.06]	.	.	.	.
LU160:PLA	1	0.79	-4.63	[-6.44; -2.83]	-5.30	[-7.33; -3.27]	-2.06	[-6.04; 1.92]	-3.24	[-7.71; 1.23]	-1.42	0.1550
LU160:QUE	1	1.00	0.50	[-1.53; 2.53]	0.50	[-1.53; 2.53]	.	.	.	.	.	.
LU160:RIS	0	0	-1.62	[-4.24; 0.99]	.	.	-1.62	[-4.24; 0.99]	.	.	.	.
LU40:LU80	3	0.79	0.66	[-0.33; 1.65]	0.72	[-0.40; 1.84]	0.46	[-1.68; 2.60]	0.26	[-2.15; 2.67]	0.21	0.8307
LU40:OLA	1	0.69	2.33	[0.45; 4.21]	1.60	[-0.66; 3.86]	3.97	[0.59; 7.35]	-2.37	[-6.44; 1.70]	-1.14	0.2534
LU40:PLA	5	0.94	-1.80	[-2.71; -0.90]	-1.68	[-2.61; -0.75]	-3.73	[-7.42; -0.04]	2.05	[-1.76; 5.86]	1.06	0.2912
LU40:QUE	0	0	3.33	[1.38; 5.28]	.	.	3.33	[1.38; 5.28]	.	.	.	.
LU40:RIS	1	0.79	1.21	[-0.81; 3.23]	0.90	[-1.38; 3.18]	2.35	[-2.03; 6.73]	-1.45	[-6.38; 3.48]	-0.58	0.5648
LU80:OLA	1	0.08	1.67	[-0.29; 3.62]	6.85	[-0.26; 13.96]	1.24	[-0.79; 3.28]	5.61	[-1.78; 13.00]	1.49	0.1371
LU80:PLA	5	0.93	-2.47	[-3.32; -1.61]	-2.42	[-3.30; -1.53]	-3.15	[-6.43; 0.12]	0.74	[-2.66; 4.13]	0.43	0.6706
LU80:QUE	1	0.79	2.67	[0.86; 4.47]	2.00	[-0.03; 4.03]	5.24	[1.26; 9.22]	-3.24	[-7.71; 1.23]	-1.42	0.1550
LU80:RIS	1	0.84	0.54	[-1.45; 2.54]	1.50	[-0.68; 3.68]	-4.31	[-9.22; 0.60]	5.81	[0.43; 11.19]	2.12	0.0342
OLA:PLA	1	0.69	-4.13	[-6.00; -2.26]	-3.90	[-6.16; -1.64]	-4.64	[-7.98; -1.31]	0.74	[-3.29; 4.78]	0.36	0.7173
OLA:QUE	0	0	1.00	[-1.56; 3.56]	.	.	1.00	[-1.56; 3.56]	.	.	.	.
OLA:RIS	0	0	-1.12	[-3.78; 1.53]	.	.	-1.12	[-3.78; 1.53]	.	.	.	.
QUE:PLA	1	0.79	-5.13	[-6.94; -3.33]	-5.80	[-7.83; -3.77]	-2.56	[-6.54; 1.42]	-3.24	[-7.71; 1.23]	-1.42	0.1550
RIS:PLA	1	0.83	-3.01	[-4.99; -1.03]	-2.30	[-4.48; -0.12]	-6.38	[-11.12; -1.63]	4.08	[-1.15; 9.30]	1.53	0.1260
QUE:RIS	0	0	-2.12	[-4.74; 0.49]	.	.	-2.12	[-4.74; 0.49]	.	.	.	.

C. PANSS negative score

comparison	k	prop	nma	95%-CI	direct	95%-CI	indir.	95%-CI	Diff	95%-CI	z	p-value
LU120:LU160	0	0	1.25	[-0.39; 2.90]	.	.	1.25	[-0.39; 2.90]	.	.	.	.
LU120:LU40	3	0.87	0.09	[-0.90; 1.07]	0.07	[-0.99; 1.13]	0.22	[-2.48; 2.92]	-0.16	[-3.06; 2.74]	-0.11	0.9155
LU120:LU80	1	0.47	0.25	[-0.79; 1.29]	0.40	[-1.12; 1.92]	0.11	[-1.32; 1.55]	0.29	[-1.80; 2.37]	0.27	0.7883
LU120:OLA	1	0.64	0.77	[-0.71; 2.24]	1.00	[-0.85; 2.85]	0.35	[-2.11; 2.81]	0.65	[-2.43; 3.72]	0.41	0.6804
LU120:PLA	3	0.84	-1.26	[-2.23; -0.29]	-1.18	[-2.24; -0.12]	-1.66	[-4.06; 0.75]	0.48	[-2.15; 3.11]	0.36	0.7216
LU120:QUE	0	0	1.15	[-0.49; 2.80]	.	.	1.15	[-0.49; 2.80]	.	.	.	.
LU120:RIS	0	0	-0.01	[-1.80; 1.79]	.	.	-0.01	[-1.80; 1.79]	.	.	.	.
LU160:LU40	0	0	-1.17	[-2.68; 0.35]	.	.	-1.17	[-2.68; 0.35]	.	.	.	.
LU160:LU80	1	0.82	-1.01	[-2.38; 0.37]	-0.40	[-1.92; 1.12]	-3.82	[-7.10; -0.55]	3.42	[-0.18; 7.03]	1.86	0.0629
LU160:OLA	0	0	-0.49	[-2.40; 1.43]	.	.	-0.49	[-2.40; 1.43]	.	.	.	.
LU160:PLA	1	0.74	-2.51	[-3.91; -1.11]	-3.30	[-4.93; -1.67]	-0.27	[-3.02; 2.47]	-3.03	[-6.22; 0.16]	-1.86	0.0629
LU160:QUE	1	1.00	-0.10	[-1.62; 1.42]	-0.10	[-1.62; 1.42]	.	.	.	.	.	.
LU160:RIS	0	0	-1.26	[-3.33; 0.81]	.	.	-1.26	[-3.33; 0.81]	.	.	.	.
LU40:LU80	3	0.77	0.16	[-0.65; 0.97]	0.01	[-0.92; 0.94]	0.66	[-1.03; 2.34]	-0.65	[-2.57; 1.27]	-0.66	0.5089
LU40:OLA	1	0.65	0.68	[-0.71; 2.07]	0.20	[-1.53; 1.93]	1.56	[-0.79; 3.91]	-1.36	[-4.28; 1.56]	-0.91	0.3610
LU40:PLA	5	0.93	-1.35	[-2.10; -0.60]	-1.18	[-1.96; -0.40]	-3.59	[-6.43; -0.75]	2.41	[-0.53; 5.35]	1.61	0.1082
LU40:QUE	0	0	1.07	[-0.45; 2.58]	.	.	1.07	[-0.45; 2.58]	.	.	.	.
LU40:RIS	1	0.77	-0.10	[-1.72; 1.53]	0.10	[-1.75; 1.95]	-0.76	[-4.15; 2.64]	0.86	[-3.01; 4.72]	0.43	0.6641

LU80:OLA	1	0.18	0.52	[-0.91; 1.95]	-0.03	[-3.37; 3.31]	0.64	[-0.94; 2.22]	-0.67	[-4.36; 3.02]	-0.36	0.7216
LU80:PLA	5	0.92	-1.51	[-2.21; -0.81]	-1.34	[-2.07; -0.61]	-3.41	[-5.89; -0.92]	2.06	[-0.53; 4.65]	1.56	0.1184
LU80:QUE	1	0.82	0.91	[-0.47; 2.28]	0.30	[-1.22; 1.82]	3.72	[0.45; 7.00]	-3.42	[-7.03; 0.18]	-1.86	0.0629
LU80:RIS	1	0.76	-0.26	[-1.87; 1.36]	0.00	[-1.85; 1.85]	-1.09	[-4.42; 2.24]	1.09	[-2.72; 4.90]	0.56	0.5758
OLA:PLA	1	0.64	-2.03	[-3.41; -0.64]	-2.60	[-4.33; -0.87]	-1.02	[-3.31; 1.27]	-1.58	[-4.45; 1.29]	-1.08	0.2799
OLA:QUE	0	0	0.39	[-1.53; 2.30]	.	.	0.39	[-1.53; 2.30]	.	.	.	.
OLA:RIS	0	0	-0.77	[-2.82; 1.27]	.	.	-0.77	[-2.82; 1.27]	.	.	.	.
QUE:PLA	1	0.74	-2.41	[-3.81; -1.01]	-3.20	[-4.83; -1.57]	-0.17	[-2.92; 2.57]	-3.03	[-6.22; 0.16]	-1.86	0.0629
RIS:PLA	1	0.76	-1.25	[-2.86; 0.35]	-0.80	[-2.65; 1.05]	-2.64	[-5.89; 0.60]	1.84	[-1.89; 5.58]	0.97	0.3333
QUE:RIS	0	0	-1.16	[-3.23; 0.91]	.	.	-1.16	[-3.23; 0.91]	.	.	.	.

D. MADRS score

comparison	k	prop	nma	95%-CI	direct	95%-CI	indir.	95%-CI	Diff	95%-CI	z	p-value
HAL:LU120	0	0	-1.54	[-3.93; 0.85]	.	.	-1.54	[-3.93; 0.85]	.	.	.	.
HAL:LU160	0	0	1.23	[-1.32; 3.77]	.	.	1.23	[-1.32; 3.77]	.	.	.	.
HAL:LU20	1	1.00	-1.40	[-4.10; 1.30]	-1.40	[-4.10; 1.30]	.	.	.	.	.	.
HAL:LU40	1	0.70	-1.23	[-3.51; 1.06]	-1.60	[-4.34; 1.14]	-0.36	[-4.52; 3.80]	-1.24	[-6.22; 3.74]	-0.49	0.6265
HAL:LU80	1	0.70	0.32	[-1.96; 2.59]	-0.20	[-2.91; 2.51]	1.54	[-2.63; 5.70]	-1.74	[-6.71; 3.23]	-0.69	0.4933
HAL:OLA	0	0	0.35	[-2.19; 2.89]	.	.	0.35	[-2.19; 2.89]	.	.	.	.
HAL:PLA	1	0.70	-1.66	[-3.92; 0.60]	-0.80	[-3.50; 1.90]	-3.66	[-7.77; 0.46]	2.86	[-2.06; 7.78]	1.14	0.2551
HAL:QUE	0	0	1.13	[-1.42; 3.67]	.	.	1.13	[-1.42; 3.67]	.	.	.	.
LU120:LU160	0	0	2.77	[1.18; 4.36]	.	.	2.77	[1.18; 4.36]	.	.	.	.
LU120:LU20	0	0	0.14	[-2.26; 2.54]	.	.	0.14	[-2.26; 2.54]	.	.	.	.
LU120:LU40	2	0.96	0.31	[-0.72; 1.35]	0.35	[-0.70; 1.41]	-0.68	[-5.88; 4.53]	1.03	[-4.28; 6.34]	0.38	0.7038
LU120:LU80	1	0.62	1.86	[0.73; 2.99]	1.40	[-0.03; 2.83]	2.61	[0.77; 4.45]	-1.21	[-3.54; 1.12]	-1.02	0.3079
LU120:OLA	1	0.76	1.89	[0.52; 3.26]	1.80	[0.23; 3.37]	2.16	[-0.62; 4.95]	-0.36	[-3.56; 2.83]	-0.22	0.8234
LU120:PLA	2	0.88	-0.12	[-1.15; 0.91]	0.13	[-0.97; 1.22]	-2.00	[-5.02; 1.02]	2.13	[-1.09; 5.34]	1.30	0.1949
LU120:QUE	0	0	2.67	[1.08; 4.26]	.	.	2.67	[1.08; 4.26]	.	.	.	.
LU160:LU20	0	0	-2.63	[-5.19; -0.07]	.	.	-2.63	[-5.19; -0.07]	.	.	.	.
LU160:LU40	0	0	-2.45	[-3.99; -0.92]	.	.	-2.45	[-3.99; -0.92]	.	.	.	.
LU160:LU80	1	0.84	-0.91	[-2.22; 0.40]	-0.40	[-1.83; 1.03]	-3.58	[-6.84; -0.31]	3.18	[-0.39; 6.74]	1.75	0.0805
LU160:OLA	0	0	-0.88	[-2.71; 0.94]	.	.	-0.88	[-2.71; 0.94]	.	.	.	.
LU160:PLA	1	0.84	-2.89	[-4.20; -1.58]	-3.40	[-4.83; -1.97]	-0.22	[-3.49; 3.04]	-3.18	[-6.74; 0.39]	-1.75	0.0805
LU160:QUE	1	1.00	-0.10	[-1.53; 1.33]	-0.10	[-1.53; 1.33]	.	.	.	.	.	.
LU20:LU40	1	0.70	0.17	[-2.13; 2.48]	-0.20	[-2.95; 2.55]	1.05	[-3.16; 5.26]	-1.25	[-6.28; 3.78]	-0.49	0.6265
LU20:LU80	1	0.71	1.72	[-0.57; 4.01]	1.20	[-1.53; 3.93]	2.96	[-1.26; 7.17]	-1.76	[-6.78; 3.27]	-0.69	0.4933
LU20:OLA	0	0	1.75	[-0.81; 4.30]	.	.	1.75	[-0.81; 4.30]	.	.	.	.
LU20:PLA	1	0.70	-0.26	[-2.53; 2.01]	0.60	[-2.11; 3.31]	-2.29	[-6.45; 1.88]	2.89	[-2.08; 7.86]	1.14	0.2551
LU20:QUE	0	0	2.53	[-0.03; 5.09]	.	.	2.53	[-0.03; 5.09]	.	.	.	.
LU40:LU80	2	0.69	1.54	[0.49; 2.60]	1.08	[-0.19; 2.36]	2.55	[0.67; 4.43]	-1.46	[-3.73; 0.81]	-1.26	0.2063
LU40:OLA	1	0.83	1.57	[0.27; 2.88]	1.50	[0.07; 2.93]	1.93	[-1.20; 5.06]	-0.43	[-3.87; 3.01]	-0.24	0.8069
LU40:PLA	3	0.91	-0.43	[-1.38; 0.51]	-0.13	[-1.11; 0.86]	-3.64	[-6.82; -0.45]	3.51	[0.17; 6.85]	2.06	0.0392
LU40:QUE	0	0	2.35	[0.82; 3.89]	.	.	2.35	[0.82; 3.89]	.	.	.	.
LU80:OLA	0	0	0.03	[-1.44; 1.50]	.	.	0.03	[-1.44; 1.50]	.	.	.	.
LU80:PLA	4	0.94	-1.98	[-2.83; -1.12]	-1.92	[-2.81; -1.04]	-2.75	[-6.17; 0.67]	0.82	[-2.71; 4.36]	0.46	0.6471
LU80:QUE	1	0.84	0.81	[-0.50; 2.12]	0.30	[-1.13; 1.73]	3.48	[0.21; 6.74]	-3.18	[-6.74; 0.39]	-1.75	0.0805
OLA:PLA	1	0.73	-2.01	[-3.35; -0.67]	-2.20	[-3.77; -0.63]	-1.49	[-4.06; 1.07]	-0.71	[-3.72; 2.30]	-0.46	0.6452
OLA:QUE	0	0	0.78	[-1.04; 2.61]	.	.	0.78	[-1.04; 2.61]	.	.	.	.
QUE:PLA	1	0.84	-2.79	[-4.10; -1.48]	-3.30	[-4.73; -1.87]	-0.12	[-3.39; 3.14]	-3.18	[-6.74; 0.39]	-1.75	0.0805

E. Weight gain

comparison	k	prop	nma	95%-CI	direct	95%-CI	indir.	95%-CI	Diff	95%-CI	z	p-value
HAL:LU120	0	0	-0.14	[-1.09; 0.82]	.	.	-0.14	[-1.09; 0.82]	.	.	.	.
HAL:LU160	0	0	-0.31	[-1.38; 0.76]	.	.	-0.31	[-1.38; 0.76]	.	.	.	.
HAL:LU20	1	0.85	0.09	[-0.77; 0.96]	0.30	[-0.64; 1.24]	-1.13	[-3.41; 1.14]	1.43	[-1.02; 3.89]	1.14	0.2529
HAL:LU40	1	0.61	-0.19	[-1.06; 0.68]	0.10	[-1.02; 1.22]	-0.64	[-2.04; 0.76]	0.74	[-1.05; 2.53]	0.81	0.4176
HAL:LU80	1	0.52	-0.37	[-1.24; 0.50]	-0.80	[-2.01; 0.41]	0.10	[-1.16; 1.35]	-0.90	[-2.64; 0.85]	-1.01	0.3148
HAL:OLA	0	0	-3.26	[-4.44; -2.08]	.	.	-3.26	[-4.44; -2.08]	.	.	.	.
HAL:PLA	1	0.71	0.24	[-0.59; 1.08]	0.00	[-1.00; 1.00]	0.83	[-0.71; 2.38]	-0.83	[-2.67; 1.01]	-0.89	0.3747
HAL:QUE	0	0	-1.81	[-2.90; -0.72]	.	.	-1.81	[-2.90; -0.72]	.	.	.	.
LU120:LU160	0	0	-0.17	[-1.00; 0.66]	.	.	-0.17	[-1.00; 0.66]	.	.	.	.
LU120:LU20	0	0	0.23	[-0.46; 0.92]	.	.	0.23	[-0.46; 0.92]	.	.	.	.
LU120:LU40	2	0.83	-0.05	[-0.54; 0.44]	-0.03	[-0.57; 0.51]	-0.16	[-1.34; 1.03]	0.13	[-1.18; 1.43]	0.19	0.8503
LU120:LU80	0	0	-0.23	[-0.81; 0.34]	.	.	-0.23	[-0.81; 0.34]	.	.	.	.
LU120:OLA	1	0.97	-3.12	[-3.97; -2.27]	-3.10	[-3.96; -2.24]	-3.72	[-8.46; 1.03]	0.62	[-4.21; 5.44]	0.25	0.8021
LU120:PLA	2	0.82	0.38	[-0.11; 0.88]	0.35	[-0.20; 0.90]	0.53	[-0.65; 1.70]	-0.18	[-1.47; 1.12]	-0.27	0.7893
LU120:QUE	0	0	-1.67	[-2.53; -0.81]	.	.	-1.67	[-2.53; -0.81]	.	.	.	.
LU160:LU20	0	0	0.40	[-0.44; 1.24]	.	.	0.40	[-0.44; 1.24]	.	.	.	.
LU160:LU40	0	0	0.12	[-0.63; 0.88]	.	.	0.12	[-0.63; 0.88]	.	.	.	.
LU160:LU80	1	0.85	-0.06	[-0.74; 0.62]	0.00	[-0.74; 0.74]	-0.38	[-2.13; 1.36]	0.38	[-1.51; 2.28]	0.40	0.6925
LU160:OLA	0	0	-2.95	[-4.03; -1.86]	.	.	-2.95	[-4.03; -1.86]	.	.	.	.
LU160:PLA	1	0.86	0.55	[-0.13; 1.23]	0.50	[-0.23; 1.23]	0.90	[-0.94; 2.73]	-0.40	[-2.37; 1.58]	-0.40	0.6925
LU160:QUE	1	1.00	-1.50	[-2.34; -0.66]	-1.50	[-2.34; -0.66]	.	.	.	.	.	.
LU20:LU40	1	0.32	-0.28	[-0.87; 0.31]	-0.20	[-1.25; 0.85]	-0.32	[-1.04; 0.40]	0.12	[-1.15; 1.39]	0.18	0.8542
LU20:LU80	1	0.26	-0.46	[-1.04; 0.12]	-1.10	[-2.25; 0.05]	-0.24	[-0.92; 0.44]	-0.86	[-2.19; 0.47]	-1.27	0.2056
LU20:OLA	0	0	-3.35	[-4.33; -2.37]	.	.	-3.35	[-4.33; -2.37]	.	.	.	.
LU20:PLA	2	0.89	0.15	[-0.35; 0.66]	0.16	[-0.37; 0.70]	0.08	[-1.42; 1.57]	0.09	[-1.50; 1.67]	0.11	0.9139
LU20:QUE	0	0	-1.90	[-2.77; -1.04]	.	.	-1.90	[-2.77; -1.04]	.	.	.	.
LU40:LU80	2	0.68	-0.18	[-0.62; 0.26]	-0.22	[-0.74; 0.31]	-0.10	[-0.87; 0.67]	-0.11	[-1.05; 0.82]	-0.24	0.8110
LU40:OLA	1	0.86	-3.07	[-3.92; -2.21]	-3.10	[-4.02; -2.18]	-2.86	[-5.12; -0.61]	-0.24	[-2.67; 2.20]	-0.19	0.8486
LU40:PLA	4	0.92	0.43	[0.06; 0.81]	0.43	[0.04; 0.82]	0.47	[-0.89; 1.83]	-0.04	[-1.45; 1.38]	-0.05	0.9595
LU40:QUE	0	0	-1.62	[-2.41; -0.83]	.	.	-1.62	[-2.41; -0.83]	.	.	.	.
LU80:OLA	0	0	-2.89	[-3.79; -1.99]	.	.	-2.89	[-3.79; -1.99]	.	.	.	.
LU80:PLA	4	0.94	0.61	[0.27; 0.96]	0.61	[0.26; 0.96]	0.66	[-0.69; 2.00]	-0.05	[-1.44; 1.35]	-0.06	0.9486
LU80:QUE	1	0.86	-1.44	[-2.16; -0.72]	-1.50	[-2.28; -0.72]	-1.08	[-3.00; 0.83]	-0.42	[-2.48; 1.65]	-0.40	0.6925
OLA:PLA	1	0.88	3.50	[2.65; 4.35]	3.50	[2.59; 4.41]	3.50	[1.00; 6.01]	-0.00	[-2.67; 2.66]	-0.00	0.9984
OLA:QUE	0	0	1.45	[0.34; 2.55]	.	.	1.45	[0.34; 2.55]	.	.	.	.
QUE:PLA	1	0.87	2.05	[1.34; 2.77]	2.00	[1.23; 2.77]	2.44	[0.41; 4.46]	-0.44	[-2.60; 1.73]	-0.40	0.6925

F. Somnolence rates

comparison	k	prop	nma	95%-CI	direct	95%-CI	indir.	95%-CI	RoR	95%-CI	z	p-value
HAL:LU120	0	0	0.86	[0.36; 2.03]	.	.	0.86	[0.36; 2.03]	.	.	.	.
HAL:LU160	0	0	0.66	[0.18; 2.40]	.	.	0.66	[0.18; 2.40]	.	.	.	.
HAL:LU20	1	0.75	2.50	[0.94; 6.67]	2.22	[0.72; 6.88]	3.59	[0.50; 25.77]	0.62	[0.06; 5.98]	-0.42	0.6771
HAL:LU40	1	0.54	1.20	[0.52; 2.75]	2.09	[0.68; 6.48]	0.63	[0.19; 2.12]	3.33	[0.63; 17.54]	1.42	0.1555
HAL:LU80	1	0.79	1.24	[0.56; 2.74]	1.11	[0.45; 2.71]	1.86	[0.34; 10.29]	0.60	[0.09; 4.11]	-0.53	0.5994
HAL:OLA	0	0	1.40	[0.50; 3.93]	.	.	1.40	[0.50; 3.93]	.	.	.	.
HAL:PLA	1	0.53	2.76	[1.21; 6.26]	2.25	[0.73; 6.98]	3.45	[1.05; 11.35]	0.65	[0.13; 3.37]	-0.51	0.6095
HAL:QUE	0	0	0.33	[0.10; 1.08]	.	.	0.33	[0.10; 1.08]	.	.	.	.
LU120:LU160	0	0	0.77	[0.25; 2.41]	.	.	0.77	[0.25; 2.41]	.	.	.	.
LU120:LU20	0	0	2.92	[1.19; 7.12]	.	.	2.92	[1.19; 7.12]	.	.	.	.

LU120:LU40	3	0.92	1.40	[0.91; 2.15]	1.43	[0.91; 2.23]	1.10	[0.25; 4.95]	1.29	[0.27; 6.18]	0.32	0.7480
LU120:LU80	1	0.58	1.45	[0.85; 2.44]	1.49	[0.75; 2.96]	1.39	[0.61; 3.13]	1.07	[0.37; 3.11]	0.13	0.8969
LU120:OLA	1	0.90	1.63	[0.83; 3.18]	1.69	[0.83; 3.43]	1.17	[0.14; 9.52]	1.45	[0.16; 13.31]	0.33	0.7417
LU120:PLA	3	0.77	3.21	[1.92; 5.38]	2.93	[1.63; 5.27]	4.41	[1.49; 13.05]	0.67	[0.19; 2.28]	-0.65	0.5171
LU120:QUE	0	0	0.38	[0.14; 1.07]	.	.	0.38	[0.14; 1.07]	.	.	.	.
LU160:LU20	0	0	3.79	[1.01; 14.19]	.	.	3.79	[1.01; 14.19]	.	.	.	.
LU160:LU40	0	0	1.82	[0.59; 5.62]	.	.	1.82	[0.59; 5.62]	.	.	.	.
LU160:LU80	1	0.90	1.88	[0.67; 5.29]	1.65	[0.56; 4.91]	6.32	[0.22; 182.16]	0.26	[0.01; 8.95]	-0.74	0.4568
LU160:OLA	0	0	2.12	[0.59; 7.62]	.	.	2.12	[0.59; 7.62]	.	.	.	.
LU160:PLA	1	0.29	4.18	[1.36; 12.78]	8.07	[1.02; 63.51]	3.18	[0.84; 12.02]	2.54	[0.22; 29.61]	0.74	0.4568
LU160:QUE	1	1.00	0.50	[0.22; 1.11]	0.50	[0.22; 1.11]	.	.	.	.	.	.
LU20:LU40	1	0.42	0.48	[0.20; 1.15]	0.94	[0.25; 3.62]	0.29	[0.09; 0.92]	3.20	[0.55; 18.68]	1.29	0.1960
LU20:LU80	1	0.55	0.50	[0.21; 1.16]	0.50	[0.16; 1.59]	0.49	[0.14; 1.74]	1.02	[0.18; 5.64]	0.02	0.9826
LU20:OLA	0	0	0.56	[0.19; 1.61]	.	.	0.56	[0.19; 1.61]	.	.	.	.
LU20:PLA	2	0.84	1.10	[0.49; 2.46]	0.96	[0.40; 2.31]	2.26	[0.30; 16.87]	0.42	[0.05; 3.80]	-0.77	0.4440
LU20:QUE	0	0	0.13	[0.04; 0.45]	.	.	0.13	[0.04; 0.45]	.	.	.	.
LU40:LU80	3	0.81	1.03	[0.63; 1.69]	1.08	[0.63; 1.88]	0.84	[0.27; 2.60]	1.29	[0.37; 4.52]	0.39	0.6934
LU40:OLA	1	0.80	1.16	[0.58; 2.33]	1.12	[0.51; 2.44]	1.37	[0.29; 6.40]	0.82	[0.15; 4.61]	-0.23	0.8202
LU40:PLA	5	0.87	2.30	[1.39; 3.81]	2.06	[1.20; 3.54]	4.80	[1.19; 19.45]	0.43	[0.10; 1.92]	-1.11	0.2682
LU40:QUE	0	0	0.27	[0.10; 0.76]	.	.	0.27	[0.10; 0.76]	.	.	.	.
LU80:OLA	0	0	1.13	[0.51; 2.47]	.	.	1.13	[0.51; 2.47]	.	.	.	.
LU80:PLA	5	0.80	2.22	[1.33; 3.72]	2.38	[1.34; 4.22]	1.68	[0.53; 5.38]	1.41	[0.39; 5.17]	0.52	0.6004
LU80:QUE	1	0.88	0.26	[0.11; 0.66]	0.30	[0.11; 0.79]	0.10	[0.01; 1.45]	2.91	[0.17; 48.74]	0.74	0.4568
OLA:PLA	1	0.55	1.97	[0.92; 4.22]	2.07	[0.74; 5.79]	1.86	[0.60; 5.77]	1.12	[0.24; 5.16]	0.14	0.8867
OLA:QUE	0	0	0.23	[0.07; 0.77]	.	.	0.23	[0.07; 0.77]	.	.	.	.
QUE:PLA	1	0.25	8.42	[3.08; 23.02]	16.27	[2.19; 120.74]	6.75	[2.11; 21.58]	2.41	[0.24; 24.46]	0.74	0.4568

G. EPS rates

comparison	k	prop	nma	95%-CI	direct	95%-CI	indir.	95%-CI	RoR	95%-CI	z	p-value
HAL:LU120	0	0	2.09	[0.84; 5.21]	.	.	2.09	[0.84; 5.21]	.	.	.	.
HAL:LU160	0	0	2.33	[0.75; 7.22]	.	.	2.33	[0.75; 7.22]	.	.	.	.
HAL:LU20	1	0.59	7.66	[2.49; 23.63]	7.40	[1.72; 31.86]	8.07	[1.38; 47.35]	0.92	[0.09; 9.08]	-0.08	0.9402
HAL:LU40	1	0.61	3.18	[1.36; 7.41]	3.49	[1.18; 10.29]	2.75	[0.71; 10.70]	1.27	[0.22; 7.21]	0.27	0.7877
HAL:LU80	1	0.72	3.10	[1.24; 7.76]	3.70	[1.25; 10.92]	1.96	[0.35; 11.11]	1.88	[0.24; 14.55]	0.61	0.5435
HAL:OLA	0	0	4.66	[1.71; 12.67]	.	.	4.66	[1.71; 12.67]	.	.	.	.
HAL:PLA	1	0.48	7.02	[3.02; 16.32]	5.00	[1.47; 16.97]	9.56	[2.98; 30.69]	0.52	[0.10; 2.83]	-0.75	0.4516
HAL:QUE	0	0	5.27	[1.49; 18.62]	.	.	5.27	[1.49; 18.62]	.	.	.	.
LU120:LU160	0	0	1.11	[0.36; 3.42]	.	.	1.11	[0.36; 3.42]	.	.	.	.
LU120:LU20	0	0	3.66	[1.18; 11.30]	.	.	3.66	[1.18; 11.30]	.	.	.	.
LU120:LU40	3	0.95	1.52	[0.92; 2.51]	1.50	[0.90; 2.51]	1.93	[0.21; 17.41]	0.77	[0.08; 7.40]	-0.22	0.8246
LU120:LU80	1	0.26	1.48	[0.59; 3.71]	1.49	[0.25; 8.91]	1.47	[0.50; 4.31]	1.01	[0.13; 8.14]	0.01	0.9931
LU120:OLA	1	0.95	2.22	[1.20; 4.11]	2.29	[1.22; 4.30]	1.31	[0.09; 19.20]	1.74	[0.11; 27.34]	0.39	0.6933
LU120:PLA	3	0.84	3.35	[1.80; 6.26]	3.09	[1.57; 6.10]	5.19	[1.06; 25.30]	0.60	[0.11; 3.34]	-0.59	0.5561
LU120:QUE	0	0	2.52	[0.72; 8.83]	.	.	2.52	[0.72; 8.83]	.	.	.	.
LU160:LU20	0	0	3.30	[0.83; 13.02]	.	.	3.30	[0.83; 13.02]	.	.	.	.
LU160:LU40	0	0	1.37	[0.46; 4.07]	.	.	1.37	[0.46; 4.07]	.	.	.	.
LU160:LU80	1	0.97	1.33	[0.66; 2.70]	1.18	[0.58; 2.42]	72.30	[1.15; 4542.89]	0.02	[0.00; 1.09]	-1.92	0.0550
LU160:OLA	0	0	2.00	[0.61; 6.60]	.	.	2.00	[0.61; 6.60]	.	.	.	.
LU160:PLA	1	0.28	3.02	[1.03; 8.84]	16.13	[2.14; 121.66]	1.56	[0.44; 5.55]	10.34	[0.95; 112.29]	1.92	0.0550
LU160:QUE	1	1.00	2.27	[0.93; 5.51]	2.27	[0.93; 5.51]	.	.	.	.	.	.
LU20:LU40	1	0.43	0.42	[0.14; 1.25]	0.47	[0.09; 2.54]	0.38	[0.09; 1.61]	1.25	[0.14; 11.55]	0.20	0.8438
LU20:LU80	1	0.52	0.40	[0.12; 1.36]	0.50	[0.09; 2.69]	0.32	[0.06; 1.84]	1.56	[0.14; 17.60]	0.36	0.7206

LU20:OLA	0	0	0.61	[0.18; 2.02]	.	.	0.61	[0.18; 2.02]	.	.	.	.
LU20:PLA	2	0.91	0.92	[0.34; 2.50]	0.81	[0.28; 2.32]	3.21	[0.12; 87.01]	0.25	[0.01; 8.04]	-0.78	0.4347
LU20:QUE	0	0	0.69	[0.16; 3.03]	.	.	0.69	[0.16; 3.03]	.	.	.	.
LU40:LU80	2	0.71	0.97	[0.41; 2.34]	1.63	[0.58; 4.62]	0.28	[0.05; 1.40]	5.92	[0.86; 40.65]	1.81	0.0706
LU40:OLA	1	0.87	1.46	[0.76; 2.83]	1.24	[0.61; 2.53]	4.14	[0.69; 24.83]	0.30	[0.04; 2.06]	-1.22	0.2214
LU40:PLA	4	0.94	2.21	[1.20; 4.07]	2.00	[1.06; 3.76]	9.82	[0.85; 113.94]	0.20	[0.02; 2.56]	-1.23	0.2178
LU40:QUE	0	0	1.66	[0.49; 5.64]	.	.	1.66	[0.49; 5.64]	.	.	.	.
LU80:OLA	1	0.11	1.50	[0.55; 4.09]	5.10	[0.25; 104.69]	1.29	[0.45; 3.74]	3.94	[0.16; 96.87]	0.84	0.4015
LU80:PLA	3	0.76	2.27	[0.95; 5.41]	1.92	[0.71; 5.19]	3.87	[0.65; 22.94]	0.50	[0.06; 3.80]	-0.68	0.4993
LU80:QUE	1	0.98	1.70	[0.69; 4.19]	1.92	[0.77; 4.76]	0.00	[0.00; 2.08]	717.68	[0.87; 592412.85]	1.92	0.0550
OLA:PLA	1	0.75	1.51	[0.71; 3.20]	1.65	[0.69; 3.93]	1.15	[0.26; 5.16]	1.43	[0.25; 8.11]	0.41	0.6841
OLA:QUE	0	0	1.13	[0.30; 4.22]	.	.	1.13	[0.30; 4.22]	.	.	.	.
QUE:PLA	1	0.33	1.33	[0.40; 4.46]	7.12	[0.88; 57.84]	0.58	[0.13; 2.54]	12.33	[0.95; 160.42]	1.92	0.0550

H. Adverse dropout rates

comparison	k	prop	nma	95%-CI	direct	95%-CI	indir.	95%-CI	RoR	95%-CI	z	p-value
HAL:LU120	0	0	1.21	[0.53; 2.79]	.	.	1.21	[0.53; 2.79]	.	.	.	.
HAL:LU160	0	0	2.15	[0.56; 8.20]	.	.	2.15	[0.56; 8.20]	.	.	.	.
HAL:LU20	1	0.45	8.27	[2.13; 32.08]	10.85	[1.44; 81.83]	6.62	[1.06; 41.22]	1.64	[0.11; 24.98]	0.35	0.7228
HAL:LU40	1	0.73	1.52	[0.74; 3.14]	1.28	[0.55; 2.99]	2.44	[0.61; 9.79]	0.52	[0.10; 2.67]	-0.78	0.4379
HAL:LU80	1	0.69	1.58	[0.76; 3.31]	1.55	[0.64; 3.77]	1.66	[0.44; 6.22]	0.93	[0.19; 4.59]	-0.08	0.9334
HAL:OLA	0	0	2.12	[0.75; 6.03]	.	.	2.12	[0.75; 6.03]	.	.	.	.
HAL:PLA	1	0.46	1.94	[0.92; 4.09]	2.75	[0.92; 8.23]	1.44	[0.52; 3.98]	1.91	[0.43; 8.54]	0.85	0.3954
HAL:QUE	0	0	2.13	[0.56; 8.13]	.	.	2.13	[0.56; 8.13]	.	.	.	.
HAL:RIS	0	0	6.25	[0.76; 51.16]	.	.	6.25	[0.76; 51.16]	.	.	.	.
LU120:LU160	0	0	1.77	[0.51; 6.15]	.	.	1.77	[0.51; 6.15]	.	.	.	.
LU120:LU20	0	0	6.82	[1.85; 25.13]	.	.	6.82	[1.85; 25.13]	.	.	.	.
LU120:LU40	3	0.80	1.26	[0.76; 2.09]	1.43	[0.81; 2.51]	0.74	[0.24; 2.34]	1.92	[0.54; 6.91]	1.00	0.3158
LU120:LU80	1	0.35	1.30	[0.73; 2.34]	0.87	[0.32; 2.32]	1.63	[0.79; 3.35]	0.53	[0.16; 1.81]	-1.01	0.3131
LU120:OLA	1	0.90	1.75	[0.79; 3.86]	1.81	[0.79; 4.15]	1.28	[0.10; 16.49]	1.41	[0.10; 20.81]	0.25	0.8004
LU120:PLA	3	0.73	1.60	[0.95; 2.70]	1.75	[0.95; 3.22]	1.27	[0.47; 3.44]	1.38	[0.43; 4.44]	0.53	0.5927
LU120:QUE	0	0	1.76	[0.51; 6.10]	.	.	1.76	[0.51; 6.10]	.	.	.	.
LU120:RIS	0	0	5.15	[0.67; 39.65]	.	.	5.15	[0.67; 39.65]	.	.	.	.
LU160:LU20	0	0	3.85	[0.73; 20.46]	.	.	3.85	[0.73; 20.46]	.	.	.	.
LU160:LU40	0	0	0.71	[0.21; 2.36]	.	.	0.71	[0.21; 2.36]	.	.	.	.
LU160:LU80	1	0.81	0.74	[0.23; 2.36]	0.83	[0.23; 3.00]	0.45	[0.03; 6.52]	1.82	[0.09; 35.27]	0.40	0.6909
LU160:OLA	0	0	0.99	[0.25; 3.98]	.	.	0.99	[0.25; 3.98]	.	.	.	.
LU160:PLA	1	0.81	0.90	[0.28; 2.89]	0.81	[0.22; 2.93]	1.47	[0.10; 21.21]	0.55	[0.03; 10.62]	-0.40	0.6909
LU160:QUE	1	1.00	0.99	[0.25; 3.87]	0.99	[0.25; 3.87]	.	.	.	.	.	.
LU160:RIS	0	0	2.91	[0.29; 28.86]	.	.	2.91	[0.29; 28.86]	.	.	.	.
LU20:LU40	1	0.37	0.18	[0.05; 0.65]	0.12	[0.02; 0.92]	0.24	[0.05; 1.18]	0.49	[0.04; 6.56]	-0.54	0.5901
LU20:LU80	1	0.37	0.19	[0.05; 0.68]	0.14	[0.02; 1.13]	0.23	[0.05; 1.13]	0.63	[0.05; 8.58]	-0.35	0.7262
LU20:OLA	0	0	0.26	[0.06; 1.09]	.	.	0.26	[0.06; 1.09]	.	.	.	.
LU20:PLA	2	0.95	0.23	[0.07; 0.79]	0.27	[0.08; 0.94]	0.02	[0.00; 3.99]	16.16	[0.06; 4456.78]	0.97	0.3319
LU20:QUE	0	0	0.26	[0.05; 1.37]	.	.	0.26	[0.05; 1.37]	.	.	.	.
LU20:RIS	0	0	0.76	[0.07; 7.77]	.	.	0.76	[0.07; 7.77]	.	.	.	.
LU40:LU80	4	0.83	1.04	[0.65; 1.65]	1.15	[0.69; 1.91]	0.64	[0.21; 1.97]	1.80	[0.52; 6.21]	0.93	0.3510
LU40:OLA	1	0.72	1.39	[0.62; 3.12]	1.02	[0.40; 2.64]	3.10	[0.67; 14.33]	0.33	[0.05; 2.00]	-1.21	0.2278
LU40:PLA	6	0.92	1.27	[0.84; 1.94]	1.25	[0.81; 1.94]	1.58	[0.34; 7.36]	0.79	[0.16; 3.93]	-0.28	0.7759
LU40:QUE	0	0	1.40	[0.42; 4.64]	.	.	1.40	[0.42; 4.64]	.	.	.	.
LU40:RIS	1	0.95	4.10	[0.55; 30.32]	4.10	[0.52; 32.04]	4.18	[0.00; 23212.59]	0.98	[0.00; 6944.45]	-0.00	0.9966
LU80:OLA	0	0	1.34	[0.56; 3.18]	.	.	1.34	[0.56; 3.18]	.	.	.	.

LU80:PLA	6	0.86	1.23	[0.78; 1.94]	1.23	[0.75; 2.02]	1.21	[0.36; 4.07]	1.02	[0.27; 3.77]	0.03	0.9794
LU80:QUE	1	0.81	1.35	[0.42; 4.30]	1.20	[0.33; 4.36]	2.19	[0.15; 31.46]	0.55	[0.03; 10.60]	-0.40	0.6909
LU80:RIS	1	0.92	3.95	[0.53; 29.43]	2.98	[0.37; 24.20]	96.54	[0.08; 111616.29]	0.03	[0.00; 48.36]	-0.93	0.3540
OLA:PLA	1	0.80	0.91	[0.41; 2.04]	0.75	[0.31; 1.85]	2.02	[0.33; 12.41]	0.37	[0.05; 2.83]	-0.95	0.3406
OLA:QUE	0	0	1.00	[0.25; 4.04]	.	.	1.00	[0.25; 4.04]	.	.	.	.
OLA:RIS	0	0	2.94	[0.35; 24.87]	.	.	2.94	[0.35; 24.87]	.	.	.	.
QUE:PLA	1	0.81	0.91	[0.29; 2.91]	0.81	[0.22; 2.96]	1.48	[0.10; 21.37]	0.55	[0.03; 10.61]	-0.40	0.6909
RIS:PLA	1	0.95	0.31	[0.04; 2.30]	0.25	[0.03; 1.97]	11.85	[0.00; 63122.28]	0.02	[0.00; 144.48]	-0.86	0.3924
QUE:RIS	0	0	2.93	[0.30; 29.10]	.	.	2.93	[0.30; 29.10]	.	.	.	.

I. All-cause dropout rates

comparison	k	prop	nma	95%-CI	direct	95%-CI	indir.	95%-CI	RoR	95%-CI	z	p-value
HAL:LU120	0	0	1.04	[0.79; 1.37]	.	.	1.04	[0.79; 1.37]	.	.	.	.
HAL:LU160	0	0	1.53	[0.99; 2.35]	.	.	1.53	[0.99; 2.35]	.	.	.	.
HAL:LU20	1	0.93	1.02	[0.79; 1.32]	0.96	[0.74; 1.25]	2.10	[0.83; 5.31]	0.46	[0.18; 1.21]	-1.58	0.1140
HAL:LU40	1	0.72	1.05	[0.83; 1.34]	1.03	[0.78; 1.35]	1.13	[0.72; 1.78]	0.91	[0.53; 1.54]	-0.37	0.7137
HAL:LU80	1	0.72	1.10	[0.87; 1.40]	1.06	[0.80; 1.40]	1.23	[0.78; 1.93]	0.87	[0.51; 1.47]	-0.53	0.5942
HAL:OLA	0	0	1.30	[0.90; 1.87]	.	.	1.30	[0.90; 1.87]	.	.	.	.
HAL:PLA	1	0.64	0.96	[0.76; 1.22]	1.13	[0.85; 1.51]	0.72	[0.49; 1.07]	1.57	[0.97; 2.55]	1.82	0.0691
HAL:QUE	0	0	1.85	[1.16; 2.94]	.	.	1.85	[1.16; 2.94]	.	.	.	.
HAL:RIS	0	0	1.67	[0.98; 2.84]	.	.	1.67	[0.98; 2.84]	.	.	.	.
LU120:LU160	0	0	1.47	[0.98; 2.20]	.	.	1.47	[0.98; 2.20]	.	.	.	.
LU120:LU20	0	0	0.98	[0.76; 1.26]	.	.	0.98	[0.76; 1.26]	.	.	.	.
LU120:LU40	3	0.85	1.01	[0.85; 1.20]	1.01	[0.84; 1.22]	1.00	[0.64; 1.57]	1.01	[0.62; 1.65]	0.05	0.9593
LU120:LU80	1	0.27	1.06	[0.87; 1.28]	1.05	[0.72; 1.52]	1.06	[0.85; 1.33]	0.98	[0.64; 1.52]	-0.07	0.9439
LU120:OLA	1	0.83	1.24	[0.92; 1.67]	1.40	[1.01; 1.95]	0.70	[0.34; 1.43]	2.01	[0.92; 4.40]	1.74	0.0816
LU120:PLA	3	0.88	0.92	[0.78; 1.09]	0.90	[0.75; 1.07]	1.13	[0.70; 1.82]	0.80	[0.48; 1.33]	-0.86	0.3903
LU120:QUE	0	0	1.77	[1.14; 2.75]	.	.	1.77	[1.14; 2.75]	.	.	.	.
LU120:RIS	0	0	1.60	[0.96; 2.66]	.	.	1.60	[0.96; 2.66]	.	.	.	.
LU160:LU20	0	0	0.67	[0.44; 1.01]	.	.	0.67	[0.44; 1.01]	.	.	.	.
LU160:LU40	0	0	0.69	[0.47; 1.01]	.	.	0.69	[0.47; 1.01]	.	.	.	.
LU160:LU80	1	0.78	0.72	[0.50; 1.05]	0.80	[0.52; 1.23]	0.49	[0.22; 1.10]	1.63	[0.66; 4.03]	1.06	0.2898
LU160:OLA	0	0	0.85	[0.53; 1.36]	.	.	0.85	[0.53; 1.36]	.	.	.	.
LU160:PLA	1	0.89	0.63	[0.43; 0.91]	0.59	[0.40; 0.87]	1.13	[0.36; 3.51]	0.52	[0.16; 1.74]	-1.06	0.2898
LU160:QUE	1	1.00	1.21	[0.74; 1.97]	1.21	[0.74; 1.97]	.	.	.	.	.	.
LU160:RIS	0	0	1.09	[0.59; 2.00]	.	.	1.09	[0.59; 2.00]	.	.	.	.
LU20:LU40	1	0.59	1.03	[0.84; 1.27]	1.06	[0.81; 1.40]	0.99	[0.71; 1.37]	1.08	[0.70; 1.65]	0.34	0.7307
LU20:LU80	1	0.60	1.08	[0.87; 1.34]	1.10	[0.84; 1.45]	1.05	[0.75; 1.47]	1.05	[0.68; 1.61]	0.20	0.8391
LU20:OLA	0	0	1.27	[0.90; 1.80]	.	.	1.27	[0.90; 1.80]	.	.	.	.
LU20:PLA	2	0.74	0.94	[0.77; 1.15]	0.99	[0.79; 1.25]	0.82	[0.55; 1.21]	1.22	[0.77; 1.92]	0.85	0.3964
LU20:QUE	0	0	1.81	[1.15; 2.84]	.	.	1.81	[1.15; 2.84]	.	.	.	.
LU20:RIS	0	0	1.63	[0.97; 2.75]	.	.	1.63	[0.97; 2.75]	.	.	.	.
LU40:LU80	4	0.76	1.05	[0.90; 1.21]	1.03	[0.87; 1.21]	1.12	[0.83; 1.51]	0.92	[0.65; 1.30]	-0.49	0.6265
LU40:OLA	1	0.70	1.23	[0.91; 1.65]	1.13	[0.79; 1.61]	1.50	[0.87; 2.59]	0.75	[0.39; 1.44]	-0.86	0.3878
LU40:PLA	6	0.94	0.91	[0.81; 1.04]	0.94	[0.83; 1.07]	0.60	[0.37; 0.98]	1.56	[0.94; 2.59]	1.73	0.0832
LU40:QUE	0	0	1.75	[1.15; 2.67]	.	.	1.75	[1.15; 2.67]	.	.	.	.
LU40:RIS	1	0.85	1.58	[0.97; 2.58]	1.43	[0.84; 2.43]	2.87	[0.80; 10.30]	0.50	[0.12; 1.98]	-0.99	0.3203
LU80:OLA	1	0.06	1.18	[0.86; 1.60]	1.27	[0.36; 4.48]	1.17	[0.85; 1.61]	1.09	[0.30; 3.98]	0.13	0.8962
LU80:PLA	6	0.91	0.87	[0.77; 0.99]	0.88	[0.77; 1.01]	0.82	[0.53; 1.26]	1.07	[0.68; 1.68]	0.30	0.7644
LU80:QUE	1	0.81	1.67	[1.11; 2.53]	1.50	[0.95; 2.38]	2.65	[1.03; 6.84]	0.57	[0.20; 1.62]	-1.06	0.2898
LU80:RIS	1	0.90	1.51	[0.93; 2.47]	1.67	[0.99; 2.79]	0.65	[0.14; 2.98]	2.58	[0.51; 12.98]	1.15	0.2501
OLA:PLA	1	0.72	0.74	[0.55; 1.00]	0.82	[0.58; 1.15]	0.58	[0.33; 1.01]	1.41	[0.73; 2.71]	1.02	0.3077

```
     OLA:QUE 0    0 1.42 [0.86; 2.35]      .           .  1.42 [0.86;  2.35]    .             .     .       .
     OLA:RIS 0    0 1.29 [0.73; 2.26]      .           .  1.29 [0.73;  2.26]    .             .     .       .
     QUE:PLA 1 0.91 0.52 [0.35; 0.79]   0.49 [0.32; 0.75]  1.06 [0.27;  4.15] 0.46 [0.11;  1.93] -1.06  0.2898
     RIS:PLA 1 0.90 0.58 [0.36; 0.94]   0.59 [0.35; 0.98]  0.50 [0.11;  2.27] 1.18 [0.24;  5.82]  0.20  0.8422
     QUE:RIS 0    0 0.90 [0.48; 1.70]      .           .  0.90 [0.48;  1.70]    .             .     .       .

Legend:
 SMD - Standardized mean difference
 RR - Risk ratio
 comparison - Treatment comparison
 k          - Number of studies providing direct evidence
 prop       - Direct evidence proportion
 nma        - Estimated treatment effect (SMD or RR) in network meta-analysis
 direct     - Estimated treatment effect (SMD or RR) derived from direct evidence
 indir.     - Estimated treatment effect (SMD or RR) derived from indirect evidence
 RoR        - Ratio of Ratios (direct versus indirect)
 z          - z-value of test for disagreement (direct versus indirect)
 p-value    - p-value of test for disagreement (direct versus indirect)
```

Stable 5: Rating the confidence of NMAs using CINeMA

		PANSS total score	PANSS positive score	PANSS negative score	MADRS score	Weight gain	Somno-lence rates	EPS rates	Adverse dropout rates	All-cause dropout rates
**Network and analysis**										
Network components	Studies, Interventions, comparisons (LU and PLA only)	10, 10, 25	8, 8, 18	8, 8, 18	5, 9, 21	7, 9, 20	8,9, 21	7, 9, 22	9, 10, 24	10, 20, 50
Node and edge	Node – equal size, Edge width by number of studies	y	y	y	y	y	y	y	y	y
Analysis	Random-effect model	MD	MD	MD	MD	MD	RR	RR	RR	RR
**Issues of concern**										
Within-study bias	Low: Moderate: High (Majority RoB)	6:2:2 (y)	5:2:1(y)	5:2:1(y)	4:0:1 (y)	5:1:1 (y)	6:1:1 (y)	5:0:2 (y)	6:2:1 (y)	6:2:2 (y)
Reporting bias	Suspected all (majority trials supported by pharma)	y	y	y	y	y		y	y	y
Indirectness	Majority	y	y	y	y	y	y	y	y	y
Imprecision	Define clinically important size of effect: MD or RR	MD=15 (a)	MD=3.5 (b)	MD=3.5 (b)	MD=2 (c)	MD=0.9 (d)	RR=1	RR=1	RR=1	RR=1
Heterogeneity	The estimated value of between-study variance for the network meta-analysis	7.487	0.348	0.279	0.033	0.000	0.000	0.017	0.000	0.000
Incoherence	Global test based on a random-effects design-by-treatment interaction model<br>Local tests: Separating indirect from direct evidence	χ2=26.8, df=14, p=0.02	χ2=15.62, df=11, p=0.16	χ2=16.95, df=11, p=0.11	χ2=6.25, df=6, p=0.40	χ2=3.68, df=8, p= 0.88	χ2=6.184, d=11, p=0.86	χ2=9.28, d=9, p=0.41	χ2=9.43, d=13, p=0.74	χ2=13.92, d=14, p=0.46

(a) Hermes EDA, Sokoloff D, Stroup TS, Rosenheck RA. Minimum clinically important difference in the Positive and Negative Syndrome Scale with data from the Clinical Antipsychotic Trials of Intervention Effectiveness (CATIE). J Clin Psychiatry. 2012;73(4):526–32.
(b) The 15-point reduction of 30-item PANSS total scale was used to proportionally set the 3.5-point reduction of 7-item PANSS positive or negative subscale.
(c) Montgomery SA, Möller H-J. Is the significant superiority of escitalopram compared with other antidepressants clinically relevant? Int Clin Psychopharmacol. 2009;24(3):111–8.
(d) Lieberman JA, Stroup TS, McEvoy JP, Swartz MS, Rosenheck RA, Perkins DO, et al. Effectiveness of antipsychotic drugs in patients with chronic schizophrenia. N Engl J Med. 2005;353(12):1209–23.

A: PANSS total score reduction

Network plot

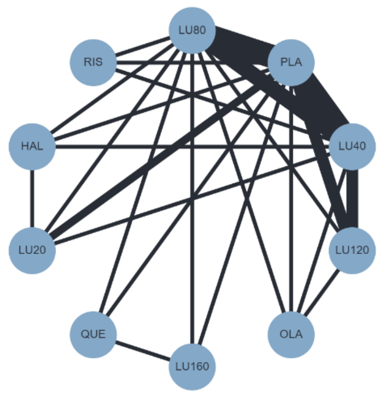

RoB Chart

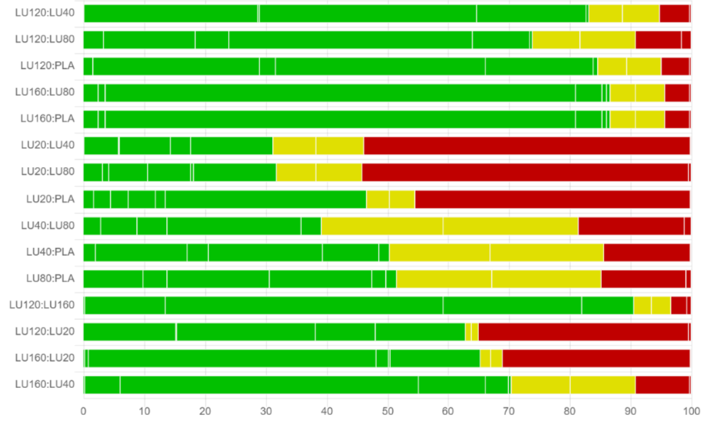

Bar chart showing the study limitations of the NMA treatment effects for PANSS total score reduction. Bars are colored according to the risk of bias level (green: low, yellow: moderate, red: high). Bar length is proportional to the percentage contribution of each direct comparison to the network estimates.

Confidence rating of comparisons

Comparison	Number of studies	Within-study bias	Reporting bias	Indirectness	Imprecision	Heterogeneity	Incoherence	Confidence rating
LU120:LU40	3	No concerns	Suspected	No concerns	No concerns	No concerns	No concerns	Low
LU120:LU80	1	No concerns	Suspected	No concerns	No concerns	No concerns	No concerns	Low
LU120:PLA	3	No concerns	Suspected	No concerns	No concerns	No concerns	No concerns	Low
LU160:LU80	1	No concerns	Suspected	No concerns	No concerns	Some concerns	No concerns	Very low
LU160:PLA	1	No concerns	Suspected	No concerns	No concerns	No concerns	No concerns	Low
LU20:LU40	1	Major concerns	Suspected	No concerns	No concerns	No concerns	No concerns	Very low
LU20:LU80	1	Major concerns	Suspected	No concerns	No concerns	No concerns	No concerns	Very low
LU20:PLA	2	No concerns	Suspected	No concerns	No concerns	No concerns	No concerns	Low
LU40:LU80	4	Some concerns	Suspected	No concerns	No concerns	No concerns	No concerns	Very low
LU40:PLA	6	No concerns	Suspected	No concerns	No concerns	No concerns	Some concerns	Very low
LU80:PLA	6	No concerns	Suspected	No concerns	No concerns	No concerns	No concerns	Low
LU120:LU160	0	No concerns	Suspected	No concerns	No concerns	Some concerns	Major concerns	Very low
LU120:LU20	0	No concerns	Suspected	No concerns	No concerns	No concerns	Major concerns	Very low
LU160:LU20	0	No concerns	Suspected	No concerns	No concerns	No concerns	Major concerns	Very low
LU160:LU40	0	No concerns	Suspected	No concerns	No concerns	Some concerns	Major concerns	Very low

B: PANSS positive score reduction

Network plot

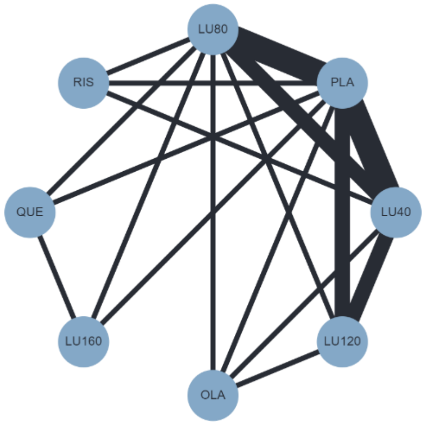

RoB Chart

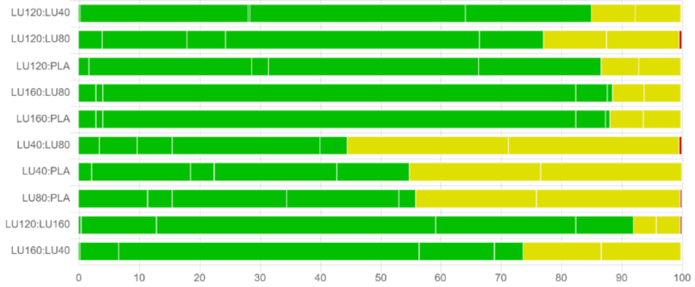

Bar chart showing the study limitations of the NMA treatment effects for PANSS positive score reduction. Bars are colored according to the risk of bias level (green: low, yellow: moderate, red: high). Bar length is proportional to the percentage contribution of each direct comparison to the network estimates.

Confidence rating of comparisons

Comparison	Number of studies	Within-study bias	Reporting bias	Indirectness	Imprecision	Heterogeneity	Incoherence	Confidence rating
LU120:LU40	3	No concerns	Suspected	No concerns	No concerns	No concerns	No concerns	Low
LU120:LU80	1	No concerns	Suspected	No concerns	No concerns	No concerns	No concerns	Low
LU120:PLA	3	No concerns	Suspected	No concerns	No concerns	Some concerns	No concerns	Very low
LU160:LU80	1	No concerns	Suspected	No concerns	No concerns	Some concerns	No concerns	Very low
LU160:PLA	1	No concerns	Suspected	No concerns	No concerns	No concerns	No concerns	Low
LU40:LU80	3	Some concerns	Suspected	No concerns	No concerns	No concerns	No concerns	Very low
LU40:PLA	5	No concerns	Suspected	No concerns	No concerns	No concerns	No concerns	Low
LU80:PLA	5	No concerns	Suspected	No concerns	No concerns	Some concerns	No concerns	Very low
LU120:LU160	0	No concerns	Suspected	No concerns	No concerns	Some concerns	No concerns	Very low
LU160:LU40	0	No concerns	Suspected	No concerns	No concerns	No concerns	No concerns	Low

C: PANSS negative score reduction

Network plot

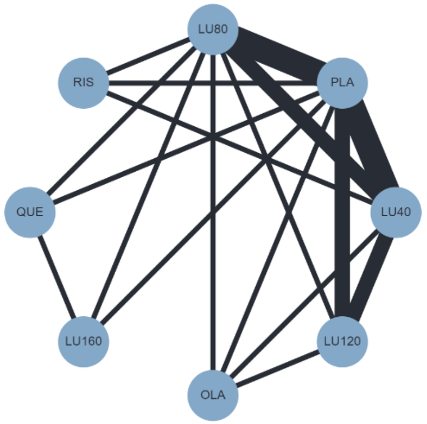

RoB Chart

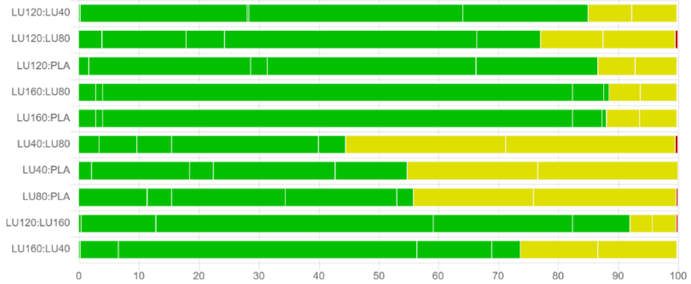

Bar chart showing the study limitations of the NMA treatment effects for PANSS negative score reduction. Bars are colored according to the risk of bias level (green: low, yellow: moderate, red: high). Bar length is proportional to the percentage contribution of each direct comparison to the network estimates.

Confidence rating of comparisons

Comparison	Number of studies	Within-study bias	Reporting bias	Indirectness	Imprecision	Heterogeneity	Incoherence	Confidence rating
LU120:LU40	3	No concerns	Suspected	No concerns	No concerns	No concerns	No concerns	Low
LU120:LU80	1	No concerns	Suspected	No concerns	No concerns	No concerns	No concerns	Low
LU120:PLA	3	No concerns	Suspected	No concerns	No concerns	Some concerns	No concerns	Very low
LU160:LU80	1	No concerns	Suspected	No concerns	No concerns	Some concerns	No concerns	Very low
LU160:PLA	1	No concerns	Suspected	No concerns	No concerns	No concerns	No concerns	Low
LU40:LU80	3	Some concerns	Suspected	No concerns	No concerns	No concerns	No concerns	Very low
LU40:PLA	5	No concerns	Suspected	No concerns	No concerns	No concerns	No concerns	Low
LU80:PLA	5	No concerns	Suspected	No concerns	No concerns	Some concerns	No concerns	Very low
LU120:LU160	0	No concerns	Suspected	No concerns	No concerns	Some concerns	No concerns	Very low
LU160:LU40	0	No concerns	Suspected	No concerns	No concerns	No concerns	No concerns	Low

D: MADRS score reduction

Network plot

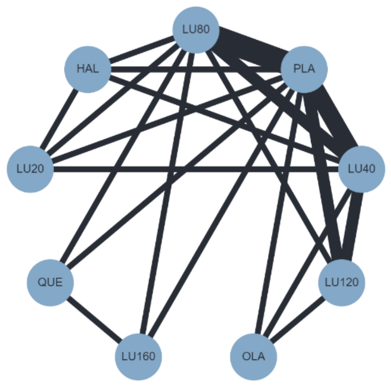

RoB Chart

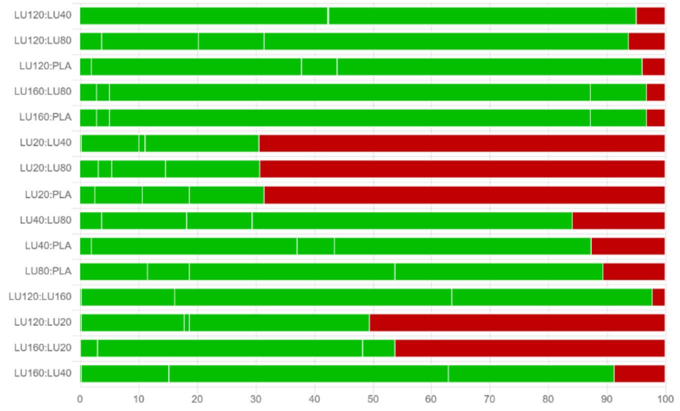

Bar chart showing the study limitations of the NMA treatment effects for MADRS score reduction. Bars are colored according to the risk of bias level (green: low, yellow: moderate, red: high). Bar length is proportional to the percentage contribution of each direct comparison to the network estimates.

Confidence rating of comparisons

Comparison	Number of studies	Within-study bias	Reporting bias	Indirectness	Imprecision	Heterogeneity	Incoherence	Confidence rating
LU120:LU40	2	No concerns	Suspected	No concerns	No concerns	No concerns	No concerns	Low
LU120:LU80	1	No concerns	Suspected	No concerns	No concerns	No concerns	No concerns	Low
LU120:PLA	2	No concerns	Suspected	No concerns	No concerns	No concerns	No concerns	Low
LU160:LU80	1	No concerns	Suspected	No concerns	Some concerns	No concerns	Some concerns	Very low
LU160:PLA	1	No concerns	Suspected	No concerns	No concerns	No concerns	Some concerns	Very low
LU20:LU40	1	Major concerns	Suspected	No concerns	Major concerns	No concerns	No concerns	Very low
LU20:LU80	1	Major concerns	Suspected	No concerns	Some concerns	No concerns	No concerns	Very low
LU20:PLA	1	Major concerns	Suspected	No concerns	Major concerns	No concerns	No concerns	Very low
LU40:LU80	2	No concerns	Suspected	No concerns	No concerns	No concerns	No concerns	Low
LU40:PLA	3	No concerns	Suspected	No concerns	No concerns	No concerns	Some concerns	Very low
LU80:PLA	4	No concerns	Suspected	No concerns	No concerns	No concerns	No concerns	Low
LU120:LU160	0	No concerns	Suspected	No concerns	No concerns	No concerns	No concerns	Low
LU120:LU20	0	Major concerns	Suspected	No concerns	Major concerns	No concerns	No concerns	Very low
LU160:LU20	0	No concerns	Suspected	No concerns	No concerns	Some concerns	No concerns	Very low
LU160:LU40	0	No concerns	Suspected	No concerns	No concerns	No concerns	No concerns	Low

E: Weight gain

Network plot

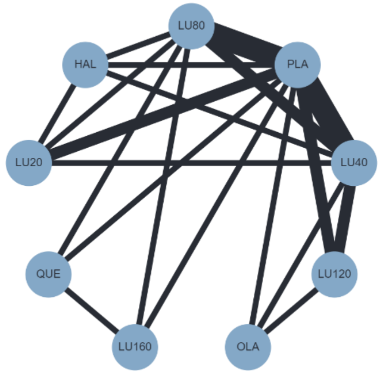

RoB Chart

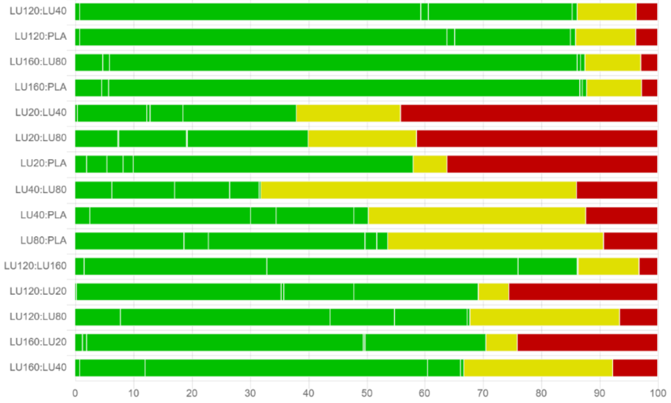

Bar chart showing the study limitations of the NMA treatment effects for weight gain. Bars are colored according to the risk of bias level (green: low, yellow: moderate, red: high). Bar length is proportional to the percentage contribution of each direct comparison to the network estimates.

Confidence rating of comparisons

Comparison	Number of studies	Within-study bias	Reporting bias	Indirectness	Imprecision	Heterogeneity	Incoherence	Confidence rating
LU120:LU40	2	No concerns	Suspected	No concerns	No concerns	No concerns	No concerns	Low
LU120:PLA	2	No concerns	Suspected	No concerns	No concerns	Some concerns	No concerns	Very low
LU160:LU80	1	No concerns	Suspected	No concerns	No concerns	No concerns	No concerns	Low
LU160:PLA	1	No concerns	Suspected	No concerns	Some concerns	No concerns	No concerns	Very low
LU20:LU40	1	Major concerns	Suspected	No concerns	No concerns	Some concerns	No concerns	Very low
LU20:LU80	1	Major concerns	Suspected	No concerns	Some concerns	No concerns	No concerns	Very low
LU20:PLA	2	No concerns	Suspected	No concerns	No concerns	No concerns	No concerns	Low
LU40:LU80	2	Some concerns	Suspected	No concerns	No concerns	No concerns	No concerns	Very low
LU40:PLA	4	No concerns	Suspected	No concerns	No concerns	No concerns	No concerns	Low
LU80:PLA	4	No concerns	Suspected	No concerns	No concerns	No concerns	No concerns	Low
LU120:LU160	0	No concerns	Suspected	No concerns	Some concerns	No concerns	No concerns	Very low
LU120:LU20	0	No concerns	Suspected	No concerns	Some concerns	No concerns	No concerns	Very low
LU120:LU80	0	No concerns	Suspected	No concerns	No concerns	Some concerns	No concerns	Very low
LU160:LU20	0	No concerns	Suspected	No concerns	Some concerns	No concerns	No concerns	Very low
LU160:LU40	0	No concerns	Suspected	No concerns	No concerns	Some concerns	No concerns	Very low

F: Somnolence rates

Network plot

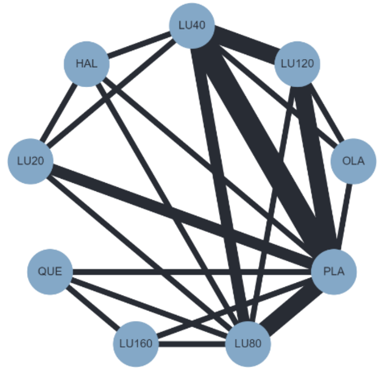

RoB Chart

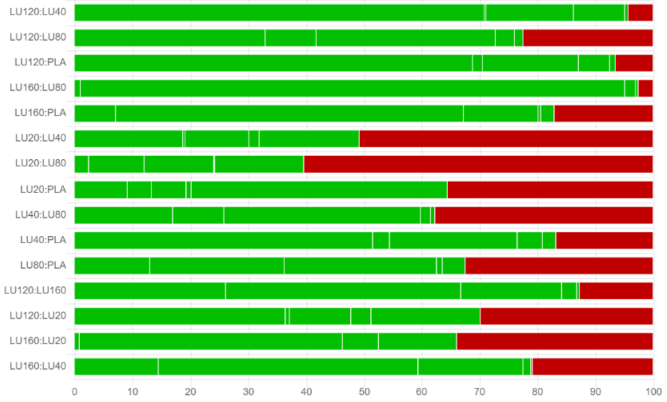

Bar chart showing the study limitations of the NMA treatment effects for somnolence rates. Bars are colored according to the risk of bias level (green: low, yellow: moderate, red: high). Bar length is proportional to the percentage contribution of each direct comparison to the network estimates.

Confidence rating of comparisons

Comparison	Number of studies	Within-study bias	Reporting bias	Indirectness	Imprecision	Heterogeneity	Incoherence	Confidence rating
LU120:LU40	3	No concerns	Suspected	No concerns	Major concerns	No concerns	No concerns	Very low
LU120:LU80	1	No concerns	Suspected	No concerns	Major concerns	No concerns	No concerns	Very low
LU120:PLA	3	No concerns	Suspected	No concerns	No concerns	No concerns	No concerns	Low
LU160:LU80	1	No concerns	Suspected	No concerns	Major concerns	No concerns	Major concerns	Very low
LU160:PLA	1	No concerns	Suspected	No concerns	Major concerns	No concerns	Major concerns	Very low
LU20:LU40	1	Major concerns	Suspected	No concerns	Major concerns	No concerns	No concerns	Very low
LU20:LU80	1	Major concerns	Suspected	No concerns	Major concerns	No concerns	No concerns	Very low
LU20:PLA	2	No concerns	Suspected	No concerns	Major concerns	No concerns	No concerns	Very low
LU40:LU80	2	No concerns	Suspected	No concerns	Major concerns	No concerns	No concerns	Very low
LU40:PLA	4	No concerns	Suspected	No concerns	No concerns	Major concerns	No concerns	Very low
LU80:PLA	3	No concerns	Suspected	No concerns	Major concerns	No concerns	No concerns	Very low
LU120:LU160	0	No concerns	Suspected	No concerns	Major concerns	No concerns	No concerns	Very low
LU120:LU20	0	No concerns	Suspected	No concerns	No concerns	Major concerns	No concerns	Very low
LU160:LU20	0	No concerns	Suspected	No concerns	Major concerns	No concerns	No concerns	Very low
LU160:LU40	0	No concerns	Suspected	No concerns	Major concerns	No concerns	No concerns	Very low

G: EPS rates

Network plot

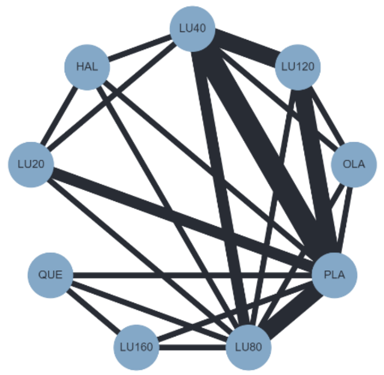

RoB Chart

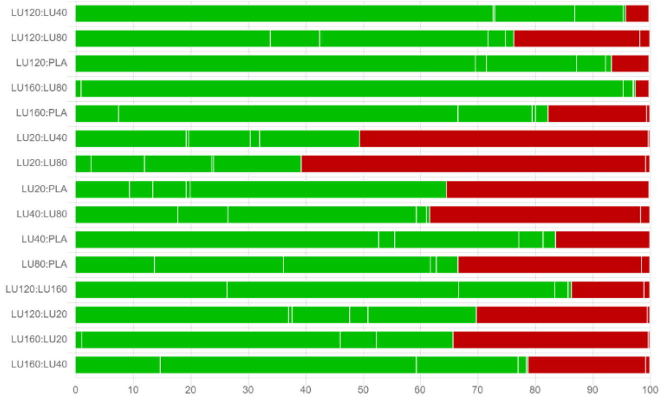

Bar chart showing the study limitations of the NMA treatment effects for EPS rates. Bars are colored according to the risk of bias level (green: low, yellow: moderate, red: high). Bar length is proportional to the percentage contribution of each direct comparison to the network estimates.

Confidence rating of comparisons

Comparison	Number of studies	Within-study bias	Reporting bias	Indirectness	Imprecision	Heterogeneity	Incoherence	Confidence rating
LU120:LU40	3	No concerns	Suspected	No concerns	Major concerns	No concerns	No concerns	Very low
LU120:LU80	1	No concerns	Suspected	No concerns	Major concerns	No concerns	No concerns	Very low
LU120:PLA	3	No concerns	Suspected	No concerns	No concerns	No concerns	No concerns	Low
LU160:LU80	1	No concerns	Suspected	No concerns	Major concerns	No concerns	Major concerns	Very low
LU160:PLA	1	No concerns	Suspected	No concerns	No concerns	Major concerns	Major concerns	Very low
LU20:LU40	1	Major concerns	Suspected	No concerns	Major concerns	No concerns	No concerns	Very low
LU20:LU80	1	Major concerns	Suspected	No concerns	Major concerns	No concerns	No concerns	Very low
LU20:PLA	2	No concerns	Suspected	No concerns	Major concerns	No concerns	No concerns	Very low
LU40:LU80	2	No concerns	Suspected	No concerns	Major concerns	No concerns	No concerns	Very low
LU40:PLA	4	No concerns	Suspected	No concerns	No concerns	No concerns	No concerns	Low
LU80:PLA	3	No concerns	Suspected	No concerns	Major concerns	No concerns	No concerns	Very low
LU120:LU160	0	No concerns	Suspected	No concerns	Major concerns	No concerns	No concerns	Very low
LU120:LU20	0	No concerns	Suspected	No concerns	No concerns	Major concerns	No concerns	Very low
LU160:LU20	0	No concerns	Suspected	No concerns	Major concerns	No concerns	No concerns	Very low
LU160:LU40	0	No concerns	Suspected	No concerns	Major concerns	No concerns	No concerns	Very low

H: Adverse dropout rates

Network plot	RoB Chart	
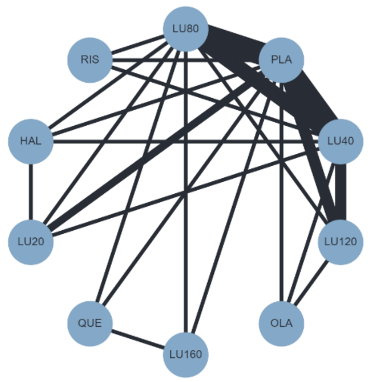	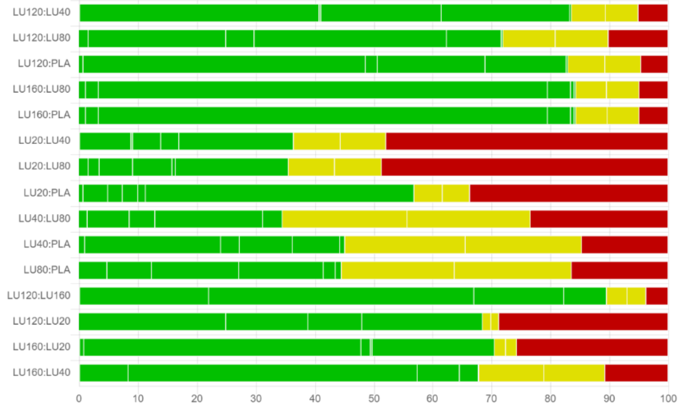	Bar chart showing the study limitations of the NMA treatment effects for adverse dropout rates. Bars are colored according to the risk of bias level (green: low, yellow: moderate, red: high). Bar length is proportional to the percentage contribution of each direct comparison to the network estimates.

Confidence rating of comparisons

Comparison	Number of studies	Within-study bias	Reporting bias	Indirectness	Imprecision	Heterogeneity	Incoherence	Confidence rating
LU120:LU40	3	No concerns	Suspected	No concerns	Major concerns	No concerns	No concerns	Very low
LU120:LU80	1	No concerns	Suspected	No concerns	Major concerns	No concerns	No concerns	Very low
LU120:PLA	3	No concerns	Suspected	No concerns	Major concerns	No concerns	No concerns	Very low
LU160:LU80	1	No concerns	Suspected	No concerns	Major concerns	No concerns	No concerns	Very low
LU160:PLA	1	No concerns	Suspected	No concerns	Major concerns	No concerns	No concerns	Very low
LU20:LU40	1	Major concerns	Suspected	No concerns	No concerns	No concerns	No concerns	Very low
LU20:LU80	1	Major concerns	Suspected	No concerns	No concerns	No concerns	No concerns	Very low
LU20:PLA	2	No concerns	Suspected	No concerns	No concerns	No concerns	No concerns	Low
LU40:LU80	4	Some concerns	Suspected	No concerns	Major concerns	No concerns	No concerns	Very low
LU40:PLA	6	No concerns	Suspected	No concerns	Major concerns	No concerns	No concerns	Very low
LU80:PLA	6	No concerns	Suspected	No concerns	Major concerns	No concerns	No concerns	Very low
LU120:LU160	0	No concerns	Suspected	No concerns	Major concerns	No concerns	No concerns	Very low
LU120:LU20	0	No concerns	Suspected	No concerns	No concerns	No concerns	No concerns	Low
LU160:LU20	0	No concerns	Suspected	No concerns	Major concerns	No concerns	No concerns	Very low
LU160:LU40	0	No concerns	Suspected	No concerns	Major concerns	No concerns	No concerns	Very low

I: All-cause dropout rates

Network plot

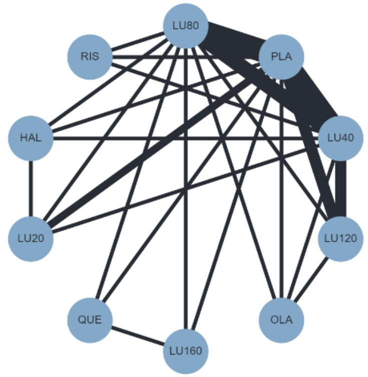

RoB Chart

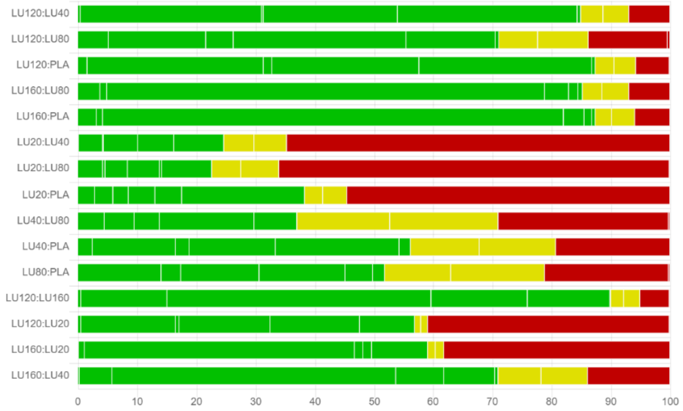

Bar chart showing the study limitations of the NMA treatment effects for all-cause dropout rates. Bars are colored according to the risk of bias level (green: low, yellow: moderate, red: high). Bar length is proportional to the percentage contribution of each direct comparison to the network estimates.

Confidence rating of comparisons

Comparison	Number of studies	Within-study bias	Reporting bias	Indirectness	Imprecision	Heterogeneity	Incoherence	Confidence rating
LU120:LU40	3	No concerns	Suspected	No concerns	Major concerns	No concerns	No concerns	Very low
LU120:LU80	1	No concerns	Suspected	No concerns	Major concerns	No concerns	No concerns	Very low
LU120:PLA	3	No concerns	Suspected	No concerns	Major concerns	No concerns	No concerns	Very low
LU160:LU80	1	No concerns	Suspected	No concerns	Major concerns	No concerns	No concerns	Very low
LU160:PLA	1	No concerns	Suspected	No concerns	No concerns	No concerns	No concerns	Low
LU20:LU40	1	Major concerns	Suspected	No concerns	Major concerns	No concerns	No concerns	Very low
LU20:LU80	1	Major concerns	Suspected	No concerns	Major concerns	No concerns	No concerns	Very low
LU20:PLA	2	Major concerns	Suspected	No concerns	Major concerns	No concerns	No concerns	Very low
LU40:LU80	4	No concerns	Suspected	No concerns	Major concerns	No concerns	No concerns	Very low
LU40:PLA	6	No concerns	Suspected	No concerns	Major concerns	No concerns	Major concerns	Very low
LU80:PLA	6	No concerns	Suspected	No concerns	No concerns	Major concerns	No concerns	Very low
LU120:LU160	0	No concerns	Suspected	No concerns	Major concerns	No concerns	No concerns	Very low
LU120:LU20	0	No concerns	Suspected	No concerns	Major concerns	No concerns	No concerns	Very low
LU160:LU20	0	No concerns	Suspected	No concerns	Major concerns	No concerns	No concerns	Very low
LU160:LU40	0	No concerns	Suspected	No concerns	Major concerns	No concerns	No concerns	Very low

SFigure 4: Funnel plots for assessing publication bias

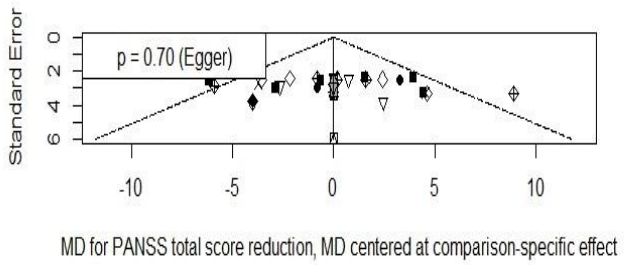

A: PANSS total score reduction

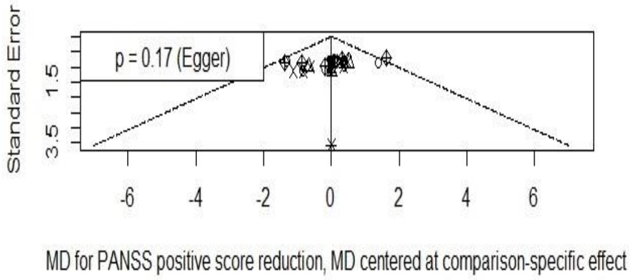

B: PANSS positive score reduction

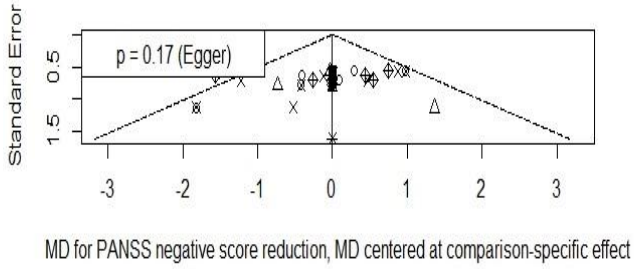

C: PANSS negative score reduction

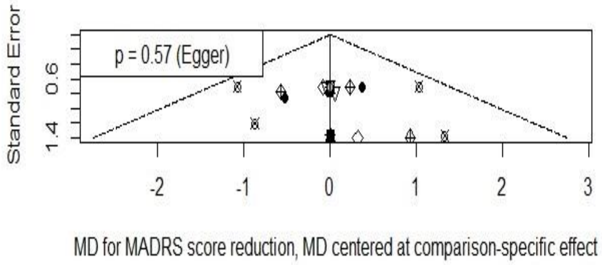

D: MADRS score reduction

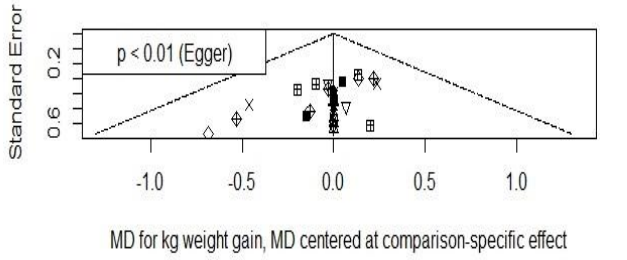

E: Weight gain

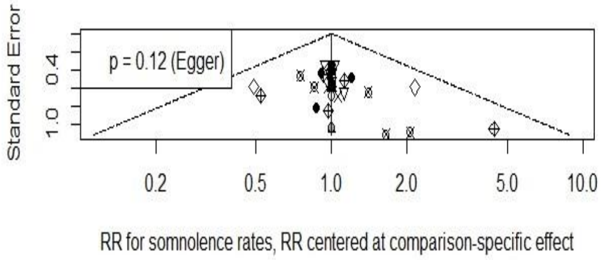

F: Somnolence rates

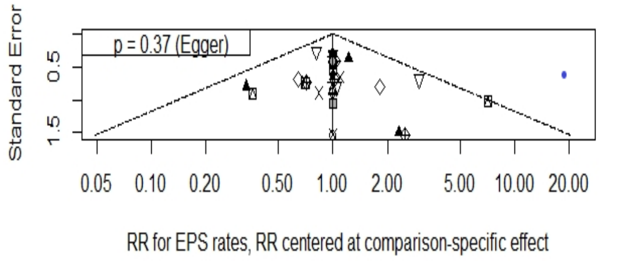

G: EPS rates

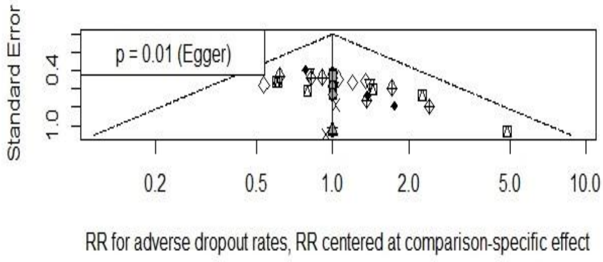

H: Adverse dropout rates

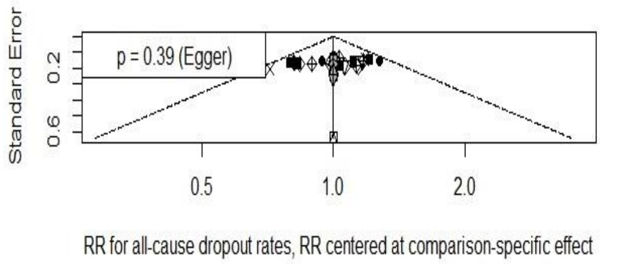

I: All-cause dropout rates

Table S6: Confidence rating and effect estimates: comparisons among lurasidone at different doses and placebo for acute schizophrenia

Note: The confidence rating tables of primary outcomes (i.e. PANSS total score reduction and all-cause dropout rates) are parts of the main manuscript.

A: PANSS positive score reduction

Treatment comparisons	Direct estimate: MD (95% CI)	Indirect estimate: MD (95% CI)	NMA estimate: MD (95% CI)	Confidence rating
LU160:PLA	-5.30 [-7.33; -3.27]	-2.06 [-6.04; 1.92]	**-4.63 [-6.44; -2.83]**	Low
LU120:PLA	-2.46 [-3.75; -1.17]	-1.22 [-4.09; 1.65]	**-2.25 [-3.43; -1.07]**	Very low
LU80:PLA	-2.42 [-3.30; -1.53]	-3.15 [-6.43; 0.12]	**-2.47 [-3.32; -1.61]**	Very low
LU40:PLA	-1.68 [-2.61; -0.75]	-3.73 [-7.42; -0.04]	**-1.80 [-2.71; -0.90]**	Low
LU160:LU80	-1.50 [-3.53; 0.53]	-4.74 [-8.72; -0.76]	**-2.17 [-3.97; -0.36]**	Very low
LU160:LU40	-	-2.83 [-4.78; -0.88]	**-2.83 [-4.78; -0.88]**	Low
LU120:LU160	-	2.38 [0.28; 4.48]	**2.38 [0.28; 4.48]**	Very low
LU120:LU80	1.10 [-0.93; 3.13]	-0.38 [-2.05; 1.28]	0.22 [-1.07; 1.50]	Low
LU120:LU40	-0.47 [-1.77; 0.82]	-0.30 [-3.43; 2.83]	-0.45 [-1.64; 0.75]	Low
LU40:LU80	0.72 [-0.40; 1.84]	0.46 [-1.68; 2.60]	0.66 [-0.33; 1.65]	Very low

B: PANSS negative score reduction

Treatment comparisons	Direct estimate: MD (95% CI)	Indirect estimate: MD (95% CI)	NMA estimate: MD (95% CI)	Confidence rating
LU160:PLA	-3.30 [-4.93; -1.67]	-0.27 [-3.02; 2.47]	**-2.51 [-3.91; -1.11]**	Low
LU120:PLA	-1.18 [-2.24; -0.12]	-1.66 [-4.06; 0.75]	**-1.26 [-2.23; -0.29]**	Very low
LU80:PLA	-1.34 [-2.07; -0.61]	-3.41 [-5.89; -0.92]	**-1.51 [-2.21; -0.81]**	Very low
LU40:PLA	-1.18 [-1.96; -0.40]	-3.59 [-6.43; -0.75]	**-1.35 [-2.10; -0.60]**	Low
LU160:LU80	-0.40 [-1.92; 1.12]	-3.82 [-7.10; -0.55]	-1.01 [-2.38; 0.37]	Very low
LU160:LU40	-	-1.17 [-2.68; 0.35]	-1.17 [-2.68; 0.35]	Low
LU120:LU160	-	1.25 [-0.39; 2.90]	1.25 [-0.39; 2.90]	Very low
LU120:LU80	0.40 [-1.12; 1.92]	0.11 [-1.32; 1.55]	0.25 [-0.79; 1.29]	Low
LU120:LU40	0.07 [-0.99; 1.13]	0.22 [-2.48; 2.92]	0.09 [-0.90; 1.07]	Low
LU40:LU80	0.01 [-0.92; 0.94]	0.66 [-1.03; 2.34]	0.16 [-0.65; 0.97]	Very low

C: MADRS score reduction

Treatment comparisons	Direct estimate: MD (95% CI)	Indirect estimate: MD (95% CI)	NMA estimate: MD (95% CI)	Confidence rating
LU160:PLA	-3.40 [-4.83; -1.97]	-0.22 [-3.49; 3.04]	**-2.89 [-4.20; -1.58]**	Very low
LU120:PLA	0.13 [-0.97; 1.22]	-2.00 [-5.02; 1.02]	-0.12 [-1.15; 0.91]	Low
LU80:PLA	-1.92 [-2.81; -1.04]	-2.75 [-6.17; 0.67]	**-1.98 [-2.83; -1.12]**	Low
LU40:PLA	-0.13 [-1.11; 0.86]	-3.64 [-6.82; -0.45]	-0.43 [-1.38; 0.51]	Very low
LU20:PLA	0.60 [-2.11; 3.31]	-2.29 [-6.45; 1.88]	-0.26 [-2.53; 2.01]	Very low
LU160:LU80	-0.40 [-1.83; 1.03]	-3.58 [-6.84; -0.31]	-0.91 [-2.22; 0.40]	Very low
LU160:LU40	-	-2.45 [-3.99; -0.92]	**-2.45 [-3.99; -0.92]**	Low
LU160:LU20	-	-2.63 [-5.19; -0.07]	**-2.63 [-5.19; -0.07]**	Very low
LU120:LU160	-	2.77 [1.18; 4.36]	**2.77 [1.18; 4.36]**	Low
LU120:LU80	1.40 [-0.03; 2.83]	2.61 [0.77; 4.45]	**1.86 [0.73; 2.99]**	Low
LU120:LU40	0.35 [-0.70; 1.41]	-0.68 [-5.88; 4.53]	0.31 [-0.72; 1.35]	Low
LU120:LU20	-	0.14 [-2.26; 2.54]	0.14 [-2.26; 2.54]	Very low
LU40:LU80	1.08 [-0.19; 2.36]	2.55 [0.67; 4.43]	**1.54 [0.49; 2.60]**	Low
LU20:LU80	1.20 [-1.53; 3.93]	2.96 [-1.26; 7.17]	1.72 [-0.57; 4.01]	Very low
LU20:LU40	-0.20 [-2.95; 2.55]	1.05 [-3.16; 5.26]	0.17 [-2.13; 2.48]	Very low

D: Weight gain.

Treatment comparisons	Direct estimate: MD (95% CI)	Indirect estimate: MD (95% CI)	NMA estimate: MD (95% CI)	Confidence rating
LU160:PLA	0.50 [-0.23; 1.23]	0.90 [-0.94; 2.73]	0.55 [-0.13; 1.23]	Very low
LU120:PLA	0.35 [-0.20; 0.90]	0.53 [-0.65; 1.70]	0.38 [-0.11; 0.88]	Very low
LU80:PLA	0.61 [0.26; 0.96]	0.66 [-0.69; 2.00]	**0.61 [0.27; 0.96]**	Low
LU40:PLA	0.43 [0.04; 0.82]	0.47 [-0.89; 1.83]	**0.43 [0.06; 0.81]**	Low
LU20:PLA	0.16 [-0.37; 0.70]	0.08 [-1.42; 1.57]	0.15 [-0.35; 0.66]	Low
LU160:LU80	0.00 [-0.74; 0.74]	-0.38 [-2.13; 1.36]	-0.06 [-0.74; 0.62]	Low
LU160:LU40	-	0.12 [-0.63; 0.88]	0.12 [-0.63; 0.88]	Very low
LU160:LU20	-	0.40 [-0.44; 1.24]	0.40 [-0.44; 1.24]	Very low
LU120:LU160	-	-0.17 [-1.00; 0.66]	-0.17 [-1.00; 0.66]	Very low
LU120:LU80	-	-0.23 [-0.81; 0.34]	-0.23 [-0.81; 0.34]	Very low
LU120:LU40	-0.03 [-0.57; 0.51]	-0.16 [-1.34; 1.03]	-0.05 [-0.54; 0.44]	Low
LU120:LU20	-	0.23 [-0.46; 0.92]	0.23 [-0.46; 0.92]	Very low
LU40:LU80	-0.22 [-0.74; 0.31]	-0.10 [-0.87; 0.67]	-0.18 [-0.62; 0.26]	Very low

E: Somnolence rates

Treatment comparisons	Direct estimate: RR (95% CI)	Indirect estimate: RR (95% CI)	NMA estimate: RR (95% CI)	Confidence rating
LU160:PLA	8.07 [1.02; 63.51]	3.18 [0.84; 12.02]	**4.18 [1.36; 12.78]**	Very low
LU120:PLA	2.93 [1.63; 5.27]	4.41 [1.49; 13.05]	**3.21 [1.92; 5.38]**	Low
LU80:PLA	2.38 [1.34; 4.22]	1.68 [0.53; 5.38]	**2.22 [1.33; 3.72]**	Very low
LU40:PLA	2.06 [1.20; 3.54]	4.80 [1.19; 19.45]	**2.30 [1.39; 3.81]**	Very low
LU20:PLA	0.96 [0.40; 2.31]	2.26 [0.30; 16.87]	1.10 [0.49; 2.46]	Very low
LU160:LU80	1.65 [0.56; 4.91]	6.32 [0.22; 182.16]	1.88 [0.67; 5.29]	Very low
LU160:LU40		1.82 [0.59; 5.62]	1.82 [0.59; 5.62]	Very low
LU160:LU20		3.79 [1.01; 14.19]	**3.79 [1.01; 14.19]**	Very low
LU120:LU160		0.77 [0.25; 2.41]	0.77 [0.25; 2.41]	Very low
LU120:LU80	1.49 [0.75; 2.96]	1.39 [0.61; 3.13]	1.45 [0.85; 2.44]	Very low
LU120:LU40	1.43 [0.91; 2.23]	1.10 [0.25; 4.95]	1.40 [0.91; 2.15]	Very low
LU120:LU20		2.92 [1.19; 7.12]	**2.92 [1.19; 7.12]**	Very low
LU40:LU80	1.08 [0.63; 1.88]	0.84 [0.27; 2.60]	1.03 [0.63; 1.69]	Very low
LU20:LU80	0.50 [0.16; 1.59]	0.49 [0.14; 1.74]	0.50 [0.21; 1.16]	Very low
LU20:LU40	0.94 [0.25; 3.62]	0.29 [0.09; 0.92]	0.48 [0.20; 1.15]	Very low

F: EPS rates

Treatment comparisons	Direct estimate: RR (95% CI)	Indirect estimate: RR (95% CI)	NMA estimate: RR (95% CI)	Confidence rating
LU160:PLA	16.13 [2.14; 121.66]	1.56 [0.44; 5.55]	**3.02 [1.03; 8.84]**	Very low
LU120:PLA	3.09 [1.57; 6.10]	5.19 [1.06; 25.30]	**3.35 [1.80; 6.26]**	Low
LU80:PLA	1.92 [0.71; 5.19]	3.87 [0.65; 22.94]	2.27 [0.95;5.41]	Very low
LU40:PLA	2.00 [1.06; 3.76]	9.82 [0.85; 113.94]	**2.21 [1.20; 4.07]**	Very low
LU20:PLA	0.81 [0.28; 2.32]	3.21 [0.12; 87.01]	0.92 [0.34; 2.50]	Very low
LU160:LU80	1.18 [0.58; 2.42]	72.30 [1.15; 4542.89]	1.33 [0.66; 2.70]	Very low
LU160:LU40	-	1.37 [0.46; 4.07]	1.37 [0.46; 4.07]	Very low
LU160:LU20	-	3.30 [0.83; 13.02]	3.30 [0.83; 13.02]	Very low
LU120:LU160	-	1.11 [0.36; 3.42]	1.11 [0.36; 3.42]	Very low
LU120:LU80	1.49 [0.25; 8.91]	1.47 [0.50; 4.31]	1.48 [0.59; 3.71]	Very low
LU120:LU40	1.50 [0.90; 2.51]	1.93 [0.21; 17.41]	1.52 [0.92; 2.51]	Very low
LU120:LU20	-	3.66 [1.18; 11.30]	**3.66 [1.18; 11.30]**	Very low
LU40:LU80	1.63 [0.58; 4.62]	0.28 [0.05; 1.40]	0.97 [0.41; 2.34]	Very low
LU20:LU80	0.50 [0.09; 2.69]	0.32 [0.06; 1.84]	0.40 [0.12; 1.36]	Very low
LU20:LU40	0.47 [0.09; 2.54]	0.38 [0.09; 1.61]	0.42 [0.14; 1.25]	Very low

G: Adverse dropout rates

Treatment comparisons	Direct estimate: RR (95% CI)	Indirect estimate: RR (95% CI)	NMA estimate: RR (95% CI)	Confidence rating
LU160:PLA	0.81 [0.22; 2.93]	1.47 [0.10; 21.21]	0.90 [0.28; 2.89]	Very low
LU120:PLA	1.75 [0.95; 3.22]	1.27 [0.47; 3.44]	1.60 [0.95; 2.70]	Very low
LU80:PLA	1.23 [0.75; 2.02]	1.21 [0.36; 4.07]	1.23 [0.78; 1.94]	Very low
LU40:PLA	1.25 [0.81; 1.94]	1.58 [0.34; 7.36]	1.27 [0.84; 1.94]	Very low
LU20:PLA	0.27 [0.08; 0.94]	0.02 [0.00; 3.99]	**0.23 [0.07; 0.79]**	Low
LU160:LU80	0.83 [0.23; 3.00]	0.45 [0.03; 6.52]	0.74 [0.23; 2.36]	Very low
LU160:LU40	-	0.71 [0.21; 2.36]	0.71 [0.21; 2.36]	Very low
LU160:LU20	-	3.85 [0.73; 20.46]	3.85 [0.73; 20.46]	Very low
LU120:LU160	-	1.77 [0.51; 6.15]	1.77 [0.51; 6.15]	Very low
LU120:LU80	0.87 [0.32; 2.32]	1.63 [0.79; 3.35]	1.30 [0.73; 2.34]	Very low
LU120:LU40	1.43 [0.81; 2.51]	0.74 [0.24; 2.34]	1.26 [0.76; 2.09]	Very low
LU120:LU20	-	6.82 [1.85; 25.13]	**6.82 [1.85; 25.13]**	Low
LU40:LU80	1.15 [0.69; 1.91]	0.64 [0.21; 1.97]	1.04 [0.65; 1.65]	Very low
LU20:LU80	0.14 [0.02; 1.13]	0.23 [0.05; 1.13]	0.19 [0.05; 0.68]	Very low
LU20:LU40	0.12 [0.02; 0.92]	0.24 [0.05; 1.18]	0.18 [0.05; 0.65]	Very low

PLA: Placebo; LU20, LU40, LU80, LU120, LU160: Lurasidone 20, 40, 80, 120, 160 mg/day, respectively
